# Characterization of Human Thymic Exosomes

**DOI:** 10.1371/journal.pone.0067554

**Published:** 2013-07-02

**Authors:** Gabriel Skogberg, Judith Gudmundsdottir, Sjoerd van der Post, Kerstin Sandström, Sören Bruhn, Mikael Benson, Lucia Mincheva-Nilsson, Vladimir Baranov, Esbjörn Telemo, Olov Ekwall

**Affiliations:** 1 Dept of Rheumatology and Inflammation Research at the Institute of Medicine, University of Gothenburg, Gothenburg, Sweden; 2 Dept of Pediatrics at the Institute of Clinical Sciences, University of Gothenburg, Gothenburg, Sweden; 3 The Proteomics Core Facility, University of Gothenburg, Gothenburg, Sweden; 4 Department of Pediatric Anesthesia and Intensive Care at the Sahlgrenska Academy, University of Gothenburg, Gothenburg, Sweden; 5 The Center for Invidualized Medication, Linköping University, Linköping, Sweden; 6 Department of Pediatrics, Linköping University, Linköping, Sweden; 7 Department of Clinical Microbiology Division of Clinical Immunology, Umeå University, Umeå, Sweden; University of California, San Francisco, United States of America

## Abstract

Exosomes are nanosized membrane-bound vesicles that are released by various cell types and are capable of carrying proteins, lipids and RNAs which can be delivered to recipient cells. Exosomes play a role in intercellular communication and have been described to mediate immunologic information. In this article we report the first isolation and characterization of exosomes from human thymic tissue. Using electron microscopy, particle size determination, density gradient measurement, flow cytometry, proteomic analysis and microRNA profiling we describe the morphology, size, density, protein composition and microRNA content of human thymic exosomes. The thymic exosomes share characteristics with previously described exosomes such as antigen presentation molecules, but they also exhibit thymus specific features regarding surface markers, protein content and microRNA profile. Interestingly, thymic exosomes carry proteins that have a tissue restricted expression in the periphery which may suggest a role in T cell selection and the induction of central tolerance. We speculate that thymic exosomes may provide the means for intercellular information exchange necessary for negative selection and regulatory T cell formation of the developing thymocytes within the human thymic medulla.

## Introduction

Extracellular vesicles (EVs) are membrane bound vesicles shed from various types of cells into the extracellular space. Exosomes, ectosomes and apoptotic bodies (ABs) are all examples of EV subgroups [1]. Exosomes are defined as 40–100 nm vesicles with a density ranging from 1.10–1.21 g/ml that carry proteins such as tumor susceptibility gene 101 (TSG101), which indicate an endocytic origin [1]. Exosomes are formed from inward budding of late endosomes and are released at the cell surface when multivesicular endosomes fuse with the outer cell membrane. Several recent studies have focused on a diverse set of exosomal functions such as antigen presentation and microRNA (miRNA) transfer between cells [2], induction of transplant tolerance [3], tolerance to fed antigens [4], tumor immunosuppression [5], transporting vesicles used by retroviruses [6] and prions [7], [8] and their ability to cross the blood brain barrier [9].

In mice, thymic exosome-like particles (ELPs) have been demonstrated to express membrane bound transforming growth factor beta (TGF-β) and MHC class II [10]. These thymic ELPs seem to posses the ability to induce the formation of natural regulatory T cells (nTregs) from thymocytes. Interestingly, intercellular transfer of thymic stromal material to thymic dendritic cells (DCs) has also been observed in mice [11]. To our knowledge, there is no previous report on the existence, characterization or function of exosomes from the human thymus.

In the thymus progenitor T cells go through sequential selection and maturation steps that determine the fate of the individual thymocyte. This process is orchestrated by surrounding cells and leads to the final export of mature CD4 and CD8 single positive T cells to the periphery. During this process nTregs, capable of regulating other T cells, are also formed. The intercellular communication, which is pivotal for this process is classically based on direct cell-cell contact, and the production of cytokines and chemokines. However, the precise mechanisms that regulate thymic T cell maturation, are still not completely clear. One crucial event in this process is the presentation of tissue restricted antigens (TRAs) to maturing thymocytes. The TRAs mirror parts of the self-antigen repertoire and are presented to maturing thymocytes during the selection process. Medullary thymic epithelial cells (mTECs) express TRAs [12] under the influence of the autoimmune regulator protein [13]. However, there is an ongoing discussion as to what extent mTECs are able to present antigens directly to thymocytes or if DCs are needed as a route to aid the presentation of mTEC derived antigens to thymocytes [14], [15], [16]. Also, adding to the complexity of antigen presence in the thymic medulla, not every mTEC express every TRA at a given timepoint [12], [17] which lowers the probability that all TRAs are to be presented by mTECs directly to the thymocytes. This could in part be compensated by the long, up to two weeks time [18], that thymocytes reside in the thymic medulla. However, recent work suggests that the time in the medulla is as short as 4–5 days [19]. If the DCs are important in the presentation of mTEC generated antigens to the thymocytes [14], the question arises regarding how these antigens are transferred from mTECs to DCs within the thymic medulla. Several proposed mechanisms have been discussed, among them; gap junctions [20], DC uptake and presentation of mTEC ABs, DC uptake and presentation of mTEC exosomes, DC nibbling on mTECs (trogocytosis) and the formation of membrane nanotubes [21] between mTECs and dendritic cells.

The same mechanisms are likely to be involved in the generation of nTregs in the thymus. It has been shown that exosomes, displaying TGF-β on their surface, can induce the development of Tregs in the periphery [22], [23]. This supports the hypothesis that thymic exosomes, co-expressing TGF-β and antigen loaded MHCII complexes, could have the capability to directly induce the development of nTregs from thymocytes. Exosomes may also carry miRNA that aids to stabilize the Treg phenotype [24].

Exosomes represent a recently discovered mode of intercellular communication with a potential role in the thymus. In this study we set out to isolate and characterize exosomes from human thymic tissue. Here, we report the existence of human thymic exosomes that carry immunologically relevant surface markers as well as TRAs indicating a role in thymic intercellular communication.

## Results

### Isolated Thymic EVs Display the Morphology, Size and Density of Exosomes

**Figure 1:**
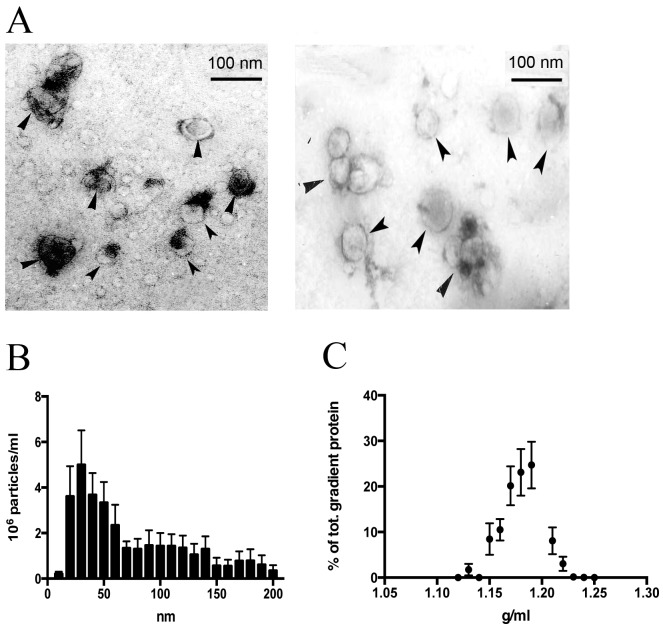
Morphological characteristics of human thymic EVs. (A) EVs from human thymic cultures visualized with electron microscopy. Arrow heads point toward EVs with a typical exosomal cup-shaped morphology and a size range of 50–100 nm. Samples from 3 individuals were analyzed with electron microscopy. (B) Size distribution of isolated EVs observed in a NanoSight LM10. The data was analyzed with Nanoparticle tracking analysis software, with a minimum expected particle size setting of 30 nm and the number of tracks analyzed for each sample exceeding 200. In agreement with exosome characteristics, most of the isolated EVs have a size of less than 100 nm. Data is presented as mean ± SEM as a result of 5 analyzed samples. (C) Density profile of isolated EVs. EVs were layered on top of a D_2_O/sucrose gradient and centrifuged at 100,000 g for 14 hours. Fractions were collected and their density and protein concentration was measured. The density of the isolated EVs peaked at 1.18–1.19 g/ml, which is within the density range of exosomes. Data is presented as mean ± SEM as a result of 5 density gradients.

**Figure 2:**
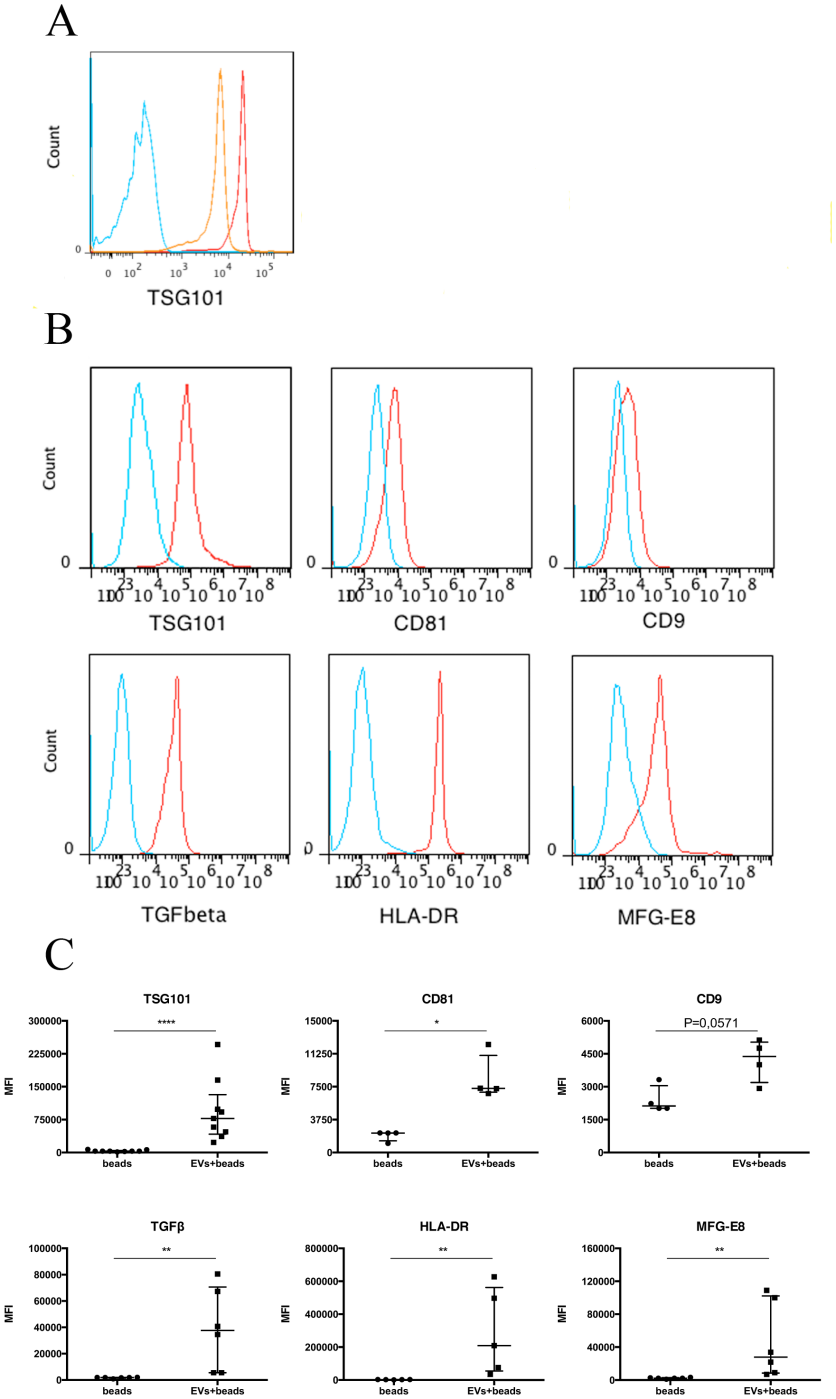
Human thymic EV surface markers analyzed by flow cytometry. (A) Histograms represent density gradient samples red (1.19 g/ml) and orange (1.15 g/ml) stained for the exosomal marker TSG101 (EVs+beads+antibodies) and blue represent negative control (beads+antibodies). (B) Red histograms represent samples (EVs+beads+antibodies) and blue represent negative controls (beads+antibodies). Hence, the isolated EVs were positive for the markers shown in the figure. A gate was created covering the latex bead population and from the bead population the histogram was created. Representative data in terms of median fluorescence intensity is shown (MFI_TSG101_ = 77 600, MFI_CD81_ = 7287, MFI_CD9_ = 4000, MFI_TGF-β_ = 34 500, MFI_HLA-DR_ = 209 000, MFI_MFG-E8_ = 33 800). (C) Experimental replicates of the flow cytometry analysis evaluated statistically with the Mann-Whitney test. P_TSG101_<0.0001, P_CD81_ = 0.0286, P_CD9_ = 0.0571, P_TGF-β_ = 0.0022, P_HLA-DR_ = 0.0079, P_MFG-E8_ = 0.0022. Data is presented as median with interquartile range.

Isolated EVs were visualized by electron microscopy to observe their size and shape. In our preparations 30–100 nm vesicles were visible in electron microscopy (Figure 1A). The EVs had typical exosomal morphology with a cup-shaped appearance in fixated samples. Size determination of the EVs performed with the NanoSight system showed a size distribution where a vast majority of the EVs were below 100 nm. (Figure 1B). This matches the size that was revealed in the electron microscope. In the NanoSight measurements, as with the electron microscopy, particles smaller than 30 nm were also observed. The density profile determined with a D_2_O/sucrose gradient is presented in figure 1C. The thymic EVs spans from 1.13 to 1.22 g/ml with the highest peak at 1.19 g/ml, which is consistent with a typical exosome density [25]. Analysis of the expression of TSG101 in fractions using flow cytometry showed the highest peak at 1.19 g/ml (Figure 2A).

### Flow Cytometry Reveals Exosomal and Immunological Markers on Thymic EVs

Surface markers were detected by flow cytometry staining for typical exosomal markers as well as other selected markers associated with immune regulation. The thymic EVs carry TSG101, CD81, HLA-DR, milk fat globulin (MFG)-E8 and CD9 to a variable extent, which all are typical markers of exosomes also from other sources. In addition the thymic EVs also carry the cytokine TGF-β (Figure 2B). Sample replicates and statistical analyses of the groups are presented in Figure 2C. Two of four samples were positive for CD54/ICAM-1 and integrin alpha(v)beta8 (α_v_β_8_) and all samples were negative for CD3, CD4, CD8 and epithelial cell adhesion molecule (EpCAM) (not shown) while CD63 staining resulted in a very subtle shift in the flow cytometry analysis which indicates very low expression (Supplemental Figure S1).

### Proteomic Analysis Identifies TRAs in Thymic EVs

**Figure 3:**
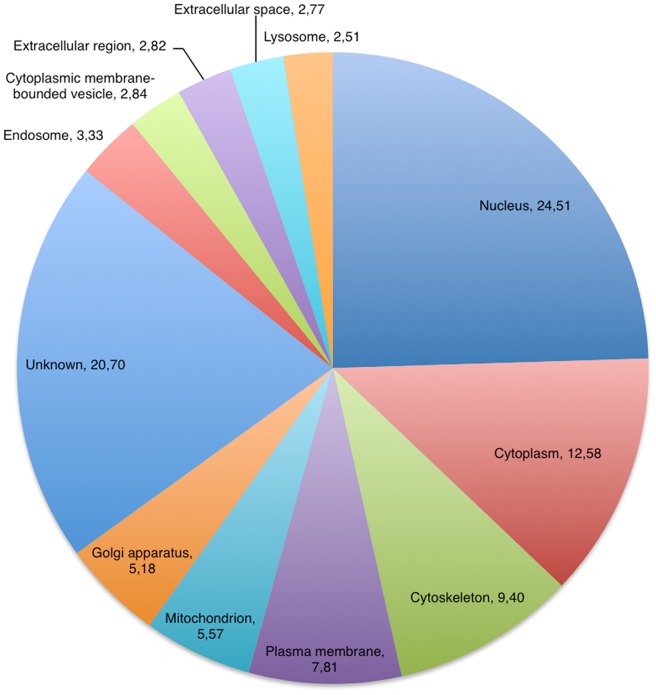
Cellular localization of EV proteins. Protein annotation was retrieved from UniProt and subcellular distribution was assigned based on gene ontology cellular component reduced to generic terms to give a broad overview of the localization. The pie chart is constructed from the shared proteins of two analyzed EV samples.

The proteomic analysis of two individual thymic EV preparations identified a total of 1853 proteins of which 1168 were shared whereas 135 and 550 proteins were unique for each individual sample. A complete list of all identified proteins is reported in supplementary results (Supplemental Table S1), and the distribution of the subcellular localization associated with the proteins is presented in figure 3. Among the identified proteins found was the programmed cell death 6-interacting protein known as ALIX that is frequently found in proteomic studies of exosomes [25]. The presence of lysosomal-associated membrane protein 2 suggests a late endosomal origin of the EVs. The epithelial marker EpCAM and DEC205, which stain both DCs and thymic epithelia [26], were both present indicating a possible epithelial origin of EVs. In addition to MHC class I and II the MHC class I-like molecules CD1a and b were also present on the EVs which are known to present self glycolipid and lipid antigens as well as microbial antigens to a subgroup of CD1d restricted T cells [27]. Several rab proteins, a protein group connected to exosome docking and fusion were also found in the thymic EVs [28].

**Figure 4:**
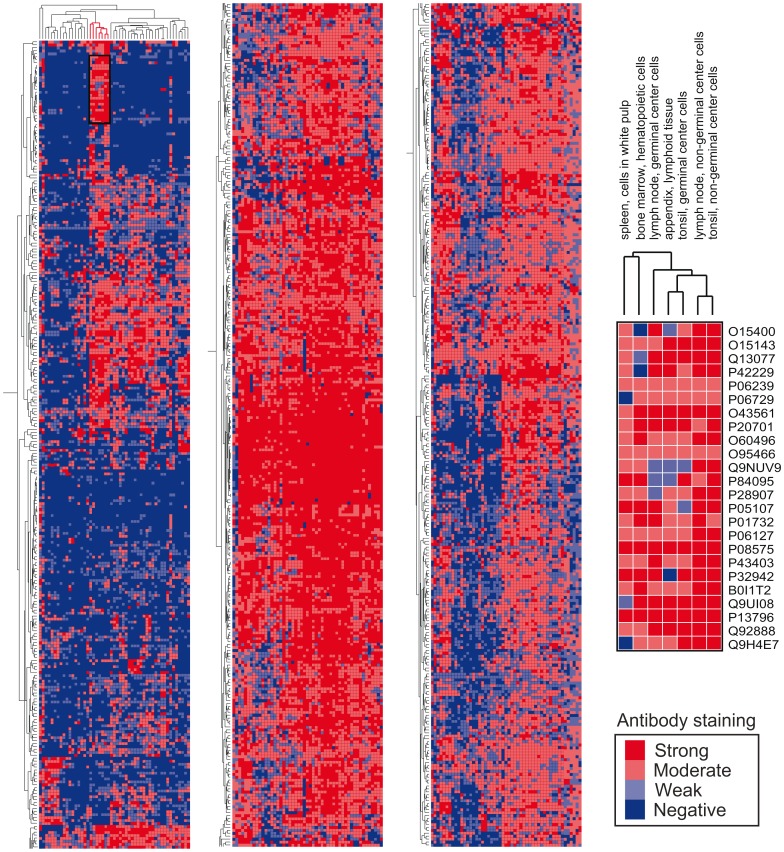
Heat-map illustrating a hierarchical cluster analysis of tissue expression of proteins identified in thymic EVs. The heat map is constructed from the proteins shared between two analyzed EV samples (proteins not yet investigated in the HPA database are not included). Proteins and tissues are hierarchally clustered according to biological function on the y- and x-axis, respectively. The expression level is graded 0–3 and illustrated by colour shift from blue to red. Encircled and enlarged is a cluster of proteins with an expression pattern in the HPA concentrated to immune-tissues. Also note the high frequency of TRAs, only expressed in a few tissues, in the left panel.

Tissue restricted expression of the identified proteins was determined using the immunohistochemistry data available in the Human Protein Atlas (HPA) [29] database, 855 of the 1168 shared proteins and 426 of the 685 sample unique proteins were represented in the database. By use of hierarchical clustering the 855 shared proteins are visualized using a heatmap (Figure 4). The heat map reveals a group of proteins that are tissue restricted, ie they are expressed only in a few tissues and therefore are TRAs. Two TRA examples are the enzyme 2′,3′-cyclic nucleotide 3′ phosphodiesterase which is normally only found in the brain, mainly glial cells, and reticulon 3 which in the HPA selectively stain neuronal and glial cells. Further TRA examples found are the muscle cell expressed tropomyosin 3, the GNAS protein which in the HPA stain cells in the gastro-intestinal tract and pancreas and rootletin which stain ciliated cells in the respiratory system and the fallopian tube. A list of all the proteins included in figure 4 and a list of the sample unique proteins represented in the HPA database are found in supplemental results (Supplemental Tables S2 and S3).

### Thymic EV miRNA Profile Suggest a Non-T Cell Origin

The four individual samples of thymic EVs that were subject to miRNA analysis shared 83 miRNAs out of the arrays 887 probes in total. A complete list of these shared miRNAs is reported in the supplementary results (Supplemental Table S4).

The miRNA content of human thymic EVs was compared to previously published data of exosome miRNAs descending from Jurkat T cells and Raji B cells [30].

**Figure 5:**
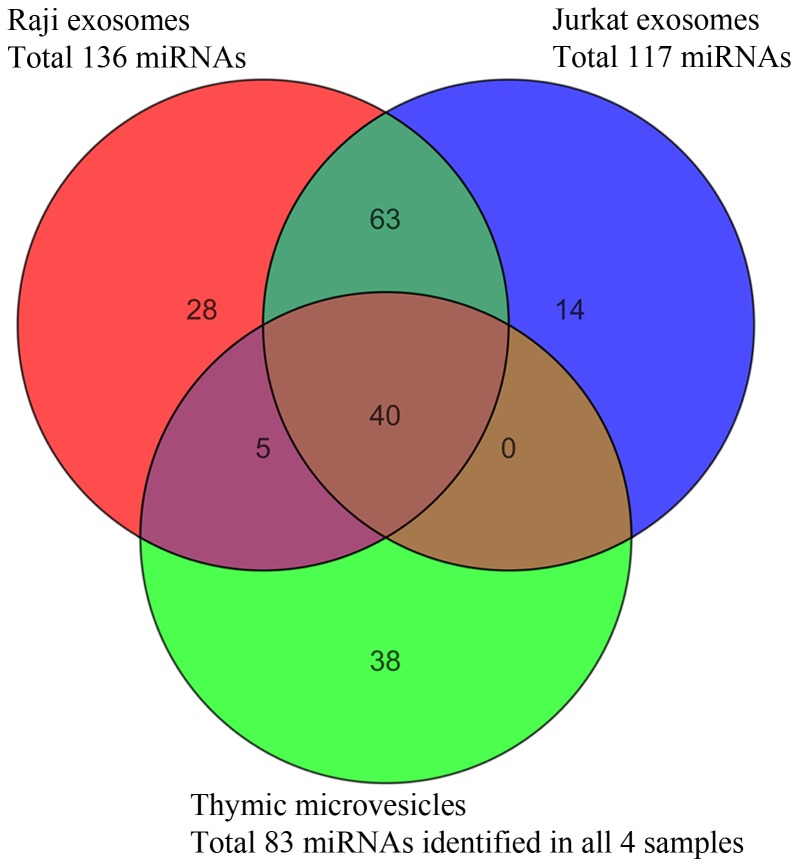
miRNAs from exosomes of different origin compared in a Venn-diagram. Green circle: shared miRNAs isolated from human thymic EVs (n = 4). Red circle: miRNAs isolated from exosomes descending from Raji B cells. Blue circle: miRNAs isolated from exosomes descending from Jurkat T cells. 40 miRNAs were shared between all three sources while 38 miRNAs were exclusively found in EVs from human thymic tissue.

For Raji cells 136 probes out of 817 probes on the array were detected, and for Jurkat cells 117 out of 815 probes. A comparison showed that 40 miRNAs were shared between all three sources (Figure 5). Furthermore we found 38 miRNAs that were exclusively expressed in EVs that derived from human thymus. In Raji-cells we found 28 and in Jurkat-cells 14 miRNAs that were exclusively expressed in exosomes from these cell-types. 5 miRNAs were exclusively in common for Raji B cells exosomes and thymic EVs whereas no detected miRNA was exclusive for Jurkat T cell exosomes and thymic EVs.

## Discussion

In this article we have described the first isolation and characterization of human thymic EVs. In terms of morphology, size, density, surface marker expression, protein content and miRNA profile the identified EVs match the characteristics of exosomes [1].

From 1 g of thymic tissue we typically isolated approximately 1 mg of exosomes. This is a relatively high yield compared to e.g. placental tissue which, using a similar isolation technique, yields approximately 25 µg of exosomes from 1 g of tissue [31]. The high yield of exosomes retrieved from thymic tissue might reflect an extensive intercellular communication within the thymus mediated by exosomes and/or an accumulation of exosomes with a non-thymic origin in the thymus. However, the likelihood of a thymic origin of the described exosomes is supported by the isolation method in which a major fraction of the exosomes is produced *ex vivo* by cultured thymic explants. When we isolate exosomes directly from thymic tissue without culturing the yield is approximately halved. In addition there was a nearmost complete lack of CD63 expression on the thymic exosomes, a marker that is typically found on non-thymic exosomes [32], [33].

In an electron microscopy fixated exosomes typically display a characteristic cup shape morphology and appear to be less than 100 nm in size. These features were also seen in the ultra structural examination of the thymic exosomes (Figure 1A). In the NanoSight size distribution plot most of the isolated thymic EVs fall into the presumed size range of exosomes since they span from 30 to 100 nm (Figure 1B). The larger particles observed in this analysis may be explained by a true variation in EV size or an aggregation of the EVs. Probably both these explanations contribute since exosomes are prone to form aggregates and the isolation technique used does not completely exclude the contamination of larger EVs.

In the flow cytometry analysis the thymic exosomes carry several markers such as TSG101, HLA-DR, CD81 and CD9 that are frequently known to be present on exosomes from other sources (Figure 2). In addition the proteomic analysis reveals the presence of lysosomal-associated membrane protein 2, which supports the classification of these thymic EVs as exosomes with a multivesicular endosome origin. The expression of HLA-DR is particularly important since it indicates that these exosomes are capable of presenting antigens or deliver MHCII-peptide complexes to cells e.g DCs. We also detected TGF-ß on the thymic exosomes, which when co-expressed with HLA-DR possibly could aid in the formation of nTregs. This mechanism has been described for mouse thymic ELPs [10]. Interestingly, the integrin α_v_β_8_ was found on the exosomes in two out of four samples tested, which could be of importance since α_v_β_8_ has been implemented in the activation of TGF-β [34]. The consistent expression of MFG-E8 on the surface of the thymic exosomes facilitates their uptake by DCs [35] and suggests that indirect presentation of exosome loaded antigens via DCs is important. The occasional expression of ICAM-1 on the thymic exosomes could indicate that a fraction of them descend from thymic DCs. ICAM-1 is expressed on DC exosomes and is important for efficient priming of T cells [36]. Further, it has also been suggested that ICAM-1 is taking part in the uptake of exosomes by DCs [37] which strengthens the idea that at least a subpopulation of thymic exosomes are homing to DCs.

When interpreting flow cytometry results it is of importance to bear in mind that one individual bead carries many exosomes (in theory, a fully occupied 4 µm bead has enough surface to bind approximately 6500 50 nm sized exosomes) with potentially different origins. One consequence of this is that a weak positive signal can be the result of either a low expression in a homogeneous population of exosomes or from a high expression found on a subpopulation of exosomes. The isolated thymic exosomes are probably a mixture of exosomes emanating from different types of thymic cells such as thymocytes, cortical TECs, mTECs, and DCs but possibly also peripheral exosomes derived from the circulation. We have so far been unable to identify a specific cellular origin using cell surface markers present on thymic cells. The lack of specific markers for thymic cell populations can be expected since the markers generally found on exosomes rarely reflect the cell surface markers of the exosome-producing cell. However, T cell derived exosomes generally carry CD3 [38], a marker that was not detected in flow cytometry of thymic exosomes in the present study. This indicates that the thymic EVs are of non-thymocyte origin and rather emanates from thymic epithelial, thymic DC or from cells of a non-thymic origin.

Thymic Evs seemed to lack CD63 expression, which may be a characteristic of human thymic exosomes that distinguishes them from exosomes of many other sources. This lack of CD63 could be necessary in the thymic milieu to enable the production of high numbers of exosomes from a relatively small population of epithelial cells. Cells lacking CD63 has previously been shown to release higher amounts of exosomes without disabling their functional capacity of the released exosomes [39].

CD3 and EpCAM were detected in the proteomic analysis but not in the flow cytometry analysis, which suggests that the contamination of other vesicles such as ABs is low. This notion is further strengthened by the size distribution plot in which a vast majority of the EVs are under 100 nm in size (Figure 1B) and by the density range. It has previously been shown that apoptotic cells in addition to larger ABs release small apoptotic vesicles but these have a density in the range of 1.24–1.28 g/ml [25] in contrast to the vesicles isolated in this work (Figure 1C). In addition it is likely that most ABs are eliminated by the 0.2 micron filtering and the 10 000 g centrifugation step.

One highly interesting observation regarding the proteomic content of the thymic exosomes is the presence of proteins that, are expressed only in a few tissues throughout the body according to the HPA (Figure 4). Since the HPA at the moment only covers about half of the human proteome, it is possible that human thymic exosomes carry an even higher number of proteins that are tissue restricted than those found in our analysis. We also found a TRA group within the list of proteins that are not shared between the two samples examined, that is, each protein is found in one or the other sample. The sample uniqueness of these TRAs could both be due to an inter-individual TRA expression or a time dependent expression of the TRAs since these samples only represent the exosomal proteomic profile at a specific time point. The function of these TRAs in the thymic exosomes is unknown, but they may be presented to developing thymocytes in the negative selection process. The possible immunological relevance of the thymic exosomes is strengthened by the identification of a cluster of proteins with an expression pattern in the HPA concentrated to immunologically relevant tissues (Figure 4).

A peculiar trait of thymic epithelia is its starvation-independent macroautophagic activity that enables the delivery of endogenous proteins/peptides to MHC class II molecules [40], [41]. Notable is that the autophagy related protein 7 is found in our proteomic analysis. Autophagy related protein 7 is absent in the Vesiclepedia database [1], [42], which covers a majority of proteins identified in exosomes. Hence, a link within the thymus between autophagosomes and the late endosomes opens the possibility for autophaged thymic proteins to be loaded on MHC II and transported out of the cell on exosomes.

The intracellular localization of thymic exosomal proteins is roughly in agreement with what has been reported for human exosomes from other sources [43], [44] (Figure 3).

Based on the Venn diagram of the miRNA content (Figure 5) it is likely that the thymic exosomes are heterogeneous in the sense that they originate from different thymic cell populations, since miRNAs are common with both Jurkat T cells and Raji B cells. It is interesting that the miRNA profile of the thymic exosomes actually displays a higher degree of similarity with exosomes from Raji B cells than with exosomes from Jurkat T cells, despite the fact that the most common cell type in the thymus is developing thymocytes. This may reflect that a substantial portion of thymic exosomes actually does not descend from thymocytes, but rather from epithelial cells and DCs. Based on previous observations of exosomes released from various epithelial cells [45], [46], and the observation that thymic epithelial cells are rich in multivesicular compartments [47], it is not unlikely that TECs are potent exosome producers. Finally, our lab has observed nanosized particles produced by cultured thymic epithelial primary cells in ongoing study (not shown).

To conclude, we report the first isolation and characterization of human thymic exosomes and speculate that they may have a role as messengers in the education of thymocytes. The exosomes may be of importance for the spreading of self antigens and miRNAs from thymic epithelia to professional antigen presenting cells in the process of negative selection and/or to promote the formation of nTregs.

## Methods

### Ethics Statement

The collection of human samples was approved by the regional ethics board of Gothenburg (no. 477-05, 2006-12-18) and all parents gave written informed consent for participation in the study. The study was performed in accordance with the declaration of Helsinki.

### Collection of Human Thymic Tissue

Human thymuses were collected during cardiac surgery from children 0–6 months of age (n = 10) at Sahlgrenska University Hospital in Gothenburg, Sweden.

### EV Isolation

EVs were isolated as previously described by Thery et al [48]. Briefly, 1–2 g of thymic tissue from each patient was fragmented (cut into small pieces) and incubated in RPMI1640 (Invitrogen, Paisley, Scotland) with 5% exosome depleted FBS (Sigma-Aldrich, St. Louis, MO, United States), 2 mM L-glutamine (Invitrogen) and penicillin/streptomycin (Sigma-Aldrich) for 8 hours. Cultures were centrifuged 10 min at 850 g. Supernatants were collected and centrifuged for 15 min at 3000 g. Further, the supernatants were spun for 30 minutes at 10 000 g followed by filtration through 0.2 µm filter. Finally the supernatants were ultracentrifuged for 70 minutes at 100,000 g to pellet the EVs. The pellets were washed in PBS and repelleted by an additional 100,000 g centrifugation.

### Electron Microscopy of the Isolated EVs

Drops of 15 µl of the isolated EVs in PBS were placed on 2% agarose to concentrate the content. Formvar/carbon-coated nickel grids were placed on top of the EV - containing drops and allowed to stand for 5–10 min to absorb the EVs on the grids and get rid of excess fluid. The grids with adherent EVs were then washed by transferring them several times to 50 µl drops of PBS for 10 min. Thereafter, the EVs on the grids were fixed in 2% paraformaldehyde in PBS for 10 min. Negative contrast staining was performed by incubating the grids with 25 µl drops of 1.9% methylcellulose (Sigma-Aldrich) containing 0.3% uranyl acetate (Ted Pella Inc., Redding, CA, United States) for 10 min on ice. Excess fluid was removed and the grids were allowed to dry before examination in a Zeiss EM 900 electron microscope (Carl Zeiss, Oberkochen, Germany).

### Size Distribution Measurement of EVs

Size distribution was estimated by the Brownian motion of the particles in a NanoSight LM10 instrument with the Nanoparticle Tracking Analysis software (NanoSight, Amesbury, UK). Samples were diluted with PBS in the optical chamber to reach a suitable concentration for the analysis. Particle concentration was evaluated in intervals of 10 nm.

### EV Density in D_2_O/sucrose Gradient

EVs were layered on top of a sucrose gradient (D_2_O/sucrose) (both from Sigma-Aldrich) with a density ranging from 1.12–1.25 and centrifuged at 100,000 g for 14 hours. Fractions (1 ml each) were collected from which the sucrose content was measured with a refractometer (VMR International, Stockholm, Sweden) giving the relative density (the relative density was also validated by weighing the fractions). The protein concentration in each fraction was measured with the Bradford protein concentration assay according to manufacturers instructions (Bio-rad Laboratories, Hercules, CA, United States).

### Flow Cytometry Analysis of EV Surface Markers

EVs were coupled to 4 µm latex beads (Invitrogen) over night at 4°C during gentle agitation. Based on the Bradford protein concentration assay, 5 µg of EVs were used together with 0.125 µl of latex beads per staining. After incubation the unspecific antibody binding to the latex beads was blocked with 0.5% BSA (Sigma-Aldrich) followed by Fc-blocking (Biolegend, San Diego, CA, United States). The beads were then stained with primary antibodies to TSG101 (Abnova, Jhongli City, Taiwan), CD9, CD81, CD63 (Becton Dickinson, Franklin Lakes, NJ, United States.), TGF-β, HLA-DR, MFG-E8, α_v_β_8_ (all R&D Systems, Minneapolis, MN, United States), ICAM-1 (eBioscience, San Diego, CA, United States), CD3, CD4, CD8 (BD Biosciences, San Jose, CA, United States) and EpCAM (Abcam, Cambridge, UK), followed by a FITC labeled secondary antibody (Sigma-Aldrich). The samples were analyzed on an Eclipse flow cytometer (iCyt, Champaign, IL, United States) using FlowJo 7.6.1 software and evaluated using median fluorescence intensity.

### Protein Identification by Tandem Masspectrometry

50 µg of the EV sample was separated by one-dimensional SDS-PAGE (4–12% Bis-Tris Novex mini-gel, Invitrogen) and visualized by Coomassie staining (Novex, Invitrogen). The complete gel lanes were excised and divided into equal slices and subjected to in-gel protein digestion with trypsin overnight at 37°C [49]. Peptides were extracted with 50% acetonitrile in 1% formic acid and the supernatant was lyophilized in a vacuum centrifuge and reconstituted in 0.2% formic acid. Two-microliter sample injections were made with an HTC-PAL autosampler (CTC Analytics AG, Zwingen, Switzerland) connected to an Agilent 1200 binary pump (Agilent Technologies, Palo Alto, CA, USA). The peptides were trapped on a precolumn (45 x 0.075 mm i.d.) and separated on a 200 x 0.050 mm column packed with 3 µm Reprosil-Pur C_18_-AQ particles (Dr. Maisch, Ammerbuch, Germany). The flow through of the analytical column was passively split to approximately 100 nl/min. A 40 min gradient 5–35% acetonitrile in 0.2% formic acid was applied for peptide separation. The LTQ-Orbitrap was operated in a data-dependent mode automatically switching between MS and MS/MS mode. Full MS scans were acquired in the orbitrap (from *m/z* 400 to 2000) with a resolution of 60.000 at *m/z* 400. The top six most intense double or triple protonated ions were selected for fragmentation in the linear ion trap using collision induced dissociation fragmentation. All tandem mass spectra were searched using MASCOT (v.2.3, Matrix Science, London, UK) against the SwissProt database (release 2011_04) concatenated with a reversed version of all entries. The search parameters were set to: species Human, MS accuracy 5 ppm, MS/MS accuracy 0.5 Da, enzyme trypsin allowing one missed cleavage, fixed modification of propionamide on cysteine and variable modifications of oxidized methionine and acetylation protein N-terminal. The false discovery rate threshold for protein identification was set to <1% at both peptide and protein level, corresponding to a minimum peptide score of 19. Protein identifications are based on a minimum of one unique peptide. Protein annotation was retrieved from UniProt [50] and subcellular distribution was assigned based on gene ontology cellular component reduced to generic terms to give a broad overview of the localization. Tissue specific expression for the identified proteins in two different patient samples was extracted from the HPA when available, converted to numerical values (0–3 for none, low, medium and high expression respectively) and evaluated by hierarchical cluster analysis, using “Euclidean distance” as similarity metric combined with complete linkage clustering. The proteomic data has been submitted to the Vesiclepedia database, http://www.microvesicles.org/(accession number: Vesiclepedia_350).

### Analysis of EV miRNA Content

The miRNA-expression analysis of human EVs was performed on samples from four individuals. 60 ng of total RNA was isolated with Qiagen miRNA mini kit, (Qiagen, Hilden, Germany) each sample was then dephosphorylated and labeled with the miRNA complete labeling kit, (Agilent) all according to the manufacturers instructions. The labeled RNA was desalted with MicroBioSpin 6 Columns (Bio-Rad) and dried in a vacuum concentrator for 90 minutes at 55°C. The dried samples were resuspended in 18 µl nuclease-free water, incubated for 5 minutes at 100°C and then transferred to ice water bath for 5 minutes. Each array (Agilent human miRNA microarray release 14.0, 8×15K), representing 894 human miRNAs was loaded with a sample volume of 45 µl and hybridized in an oven for 20 hours at 55°C with 20 rpm. After hybridization, the microarray-slides were washed, scanned (Agilent, G2505C) and extracted (Feature Extraction 10.7.3.1) according to the manufacturers instructions. The raw data was analyzed with GeneSpring 11.5.1, compromised and undetected miRNA probes were excluded. In the same way exosome derived miRNA from Jurkat [30] and Raji-cells [30] was analyzed and compared with miRNA from thymic EVs.

### Statistical Analysis

Continous variables are presented with mean ± SEM. Statistical evaluation was performed, using Prism version 6.0b (GraphPad Software), with two tailed Mann-Whitney test to calculate a P-value which was considered significant if less than 0.05 and higly significant if less than 0.01.

## Supporting Information

Figure S1 **Flow cytometry staining for CD63.** Red: (EVs+beads+antiCD63), blue: negative control (beads+antiCD63).
(TIF)

Table S1 **All proteins found in the two thymic exosomal samples.**
(PDF)

Table S2 **Shared proteins between the two individual exosomal samples.**
(PDF)

Table S3 **Proteins found in one or the other of the two induvidual exosomal samples.**
(PDF)

Table S4 **miRNAs in human thymic exosomes.**
(PDF)

## References

[1] Kalra H, Simpson RJ, Ji H, Aikawa E, Altevogt P, et al (2012) Vesiclepedia: a compendium for extracellular vesicles with continuous community annotation. PloS Biol 10: e1001450. 23271954 10.1371/journal.pbio.1001450 PMC3525526

[2] Valadi H, Ekstrom K, Bossios A, Sjostrand M, Lee JJ, et al (2007) Exosome-mediated transfer of mRNAs and microRNAs is a novel mechanism of genetic exchange between cells. Nat Cell Biol 9: 654–659. 17486113 10.1038/ncb1596

[3] Peche H, Renaudin K, Beriou G, Merieau E, Amigorena S, et al (2006) Induction of tolerance by exosomes and short-term immunosuppression in a fully MHC-mismatched rat cardiac allograft model. Am J Transplant 6: 1541–1550. 16827854 10.1111/j.1600-6143.2006.01344.x

[4] Karlsson M, Lundin S, Dahlgren U, Kahu H, Pettersson I, et al (2001) "Tolerosomes" are produced by intestinal epithelial cells. Eur J Immunol 31: 2892–2900. 11592064 10.1002/1521-4141(2001010)31:10<2892::aid-immu2892>3.0.co;2-i

[5] Yang C, Kim SH, Bianco NR, Robbins PD (2011) Tumor-derived exosomes confer antigen-specific immunosuppression in a murine delayed-type hypersensitivity model. PLoS One 6: e22517. 21829629 10.1371/journal.pone.0022517 PMC3149056

[6] Gould SJ, Booth AM, Hildreth JE (2003) The Trojan exosome hypothesis. Proc Natl Acad Sci USA 100: 10592–10597. 12947040 10.1073/pnas.1831413100 PMC196848

[7] Fevrier B, Vilette D, Archer F, Loew D, Faigle W, et al (2004) Cells release prions in association with exosomes. Proc Natl Acad Sci USA 101: 9683–9688. 15210972 10.1073/pnas.0308413101 PMC470735

[8] Kujala P, Raymond CR, Romeijn M, Godsave SF, van Kasteren SI, et al (2011) Prion uptake in the gut: identification of the first uptake and replication sites. PLoS Pathog 7: e1002449. 22216002 10.1371/journal.ppat.1002449 PMC3245311

[9] Alvarez-Erviti L, Seow Y, Yin H, Betts C, Lakhal S, et al (2011) Delivery of siRNA to the mouse brain by systemic injection of targeted exosomes. Nat Biotechnol 29: 341–345. 21423189 10.1038/nbt.1807

[10] Wang GJ, Liu Y, Qin A, Shah SV, Deng ZB, et al (2008) Thymus exosomes-like particles induce regulatory T cells. J Immunol 181: 5242–5248. 18832678 10.4049/jimmunol.181.8.5242 PMC4319673

[11] Humblet C, Rudensky AY, Kyewski B (1994) Presentation and intercellular transfer of self antigen within the thymic microenvironment: expression of the E alpha peptide-I-Ab complex by isolated thymic stromal cells. Int Immunol 6: 1949–1958. 7696212 10.1093/intimm/6.12.1949

[12] Derbinski J, Schulte A, Kyewski B, Klein L (2001) Promiscuous gene expression in medullary thymic epithelial cells mirrors the peripheral self. Nat Immunol 2: 1032–1039. 11600886 10.1038/ni723

[13] Anderson MS, Venanzi ES, Klein L, Chen Z, Berzins SP, et al (2002) Projection of an immunological self shadow within the thymus by the aire protein. Science 298: 1395–1401. 12376594 10.1126/science.1075958

[14] Gallegos AM, Bevan MJ (2004) Central tolerance to tissue-specific antigens mediated by direct and indirect antigen presentation. J Exp Med 200: 1039–1049. 15492126 10.1084/jem.20041457 PMC2211843

[15] Koble C, Kyewski B (2009) The thymic medulla: a unique microenvironment for intercellular self-antigen transfer. J Exp Med 206: 1505–1513. 19564355 10.1084/jem.20082449 PMC2715082

[16] Hubert FX, Kinkel SA, Davey GM, Phipson B, Mueller SN, et al (2011) Aire regulates the transfer of antigen from mTECs to dendritic cells for induction of thymic tolerance. Blood 118: 2462–2472. 21505196 10.1182/blood-2010-06-286393

[17] Derbinski J, Pinto S, Rosch S, Hexel K, Kyewski B (2008) Promiscuous gene expression patterns in single medullary thymic epithelial cells argue for a stochastic mechanism. Proc Natl Acad Sci USA 105: 657–662. 18180458 10.1073/pnas.0707486105 PMC2206592

[18] Scollay R, Godfrey DI (1995) Thymic emigration: conveyor belts or lucky dips? Immunol Today 16: 268–273. 7662096 10.1016/0167-5699(95)80179-0

[19] McCaughtry TM, Wilken MS, Hogquist KA (2007) Thymic emigration revisited. J Exp Med 204: 2513–2520. 17908937 10.1084/jem.20070601 PMC2118501

[20] Neijssen J, Herberts C, Drijfhout JW, Reits E, Janssen L, et al (2005) Cross-presentation by intercellular peptide transfer through gap junctions. Nature 434: 83–88. 15744304 10.1038/nature03290

[21] Millet V, Naquet P, Guinamard RR (2008) Intercellular MHC transfer between thymic epithelial and dendritic cells. Eur J Immunol 38: 1257–1263. 18412162 10.1002/eji.200737982

[22] Szajnik M, Czystowska M, Szczepanski MJ, Mandapathil M, Whiteside TL (2010) Tumor-derived microvesicles induce, expand and up-regulate biological activities of human regulatory T cells (Treg). PLoS One 5: e11469. 20661468 10.1371/journal.pone.0011469 PMC2908536

[23] Clayton A, Mitchell JP, Court J, Mason MD, Tabi Z (2007) Human tumor-derived exosomes selectively impair lymphocyte responses to interleukin-2. Cancer Res 67: 7458–7466. 17671216 10.1158/0008-5472.CAN-06-3456

[24] Rouas R, Fayyad-Kazan H, El Zein N, Lewalle P, Rothe F, et al (2009) Human natural Treg microRNA signature: role of microRNA-31 and microRNA-21 in FOXP3 expression. Eur J Immunol 39: 1608–1618. 19408243 10.1002/eji.200838509

[25] Thery C, Boussac M, Veron P, Ricciardi-Castagnoli P, Raposo G, et al (2001) Proteomic analysis of dendritic cell-derived exosomes: a secreted subcellular compartment distinct from apoptotic vesicles. J Immunol 166: 7309–7318. 11390481 10.4049/jimmunol.166.12.7309

[26] Jiang W, Swiggard WJ, Heufler C, Peng M, Mirza A, et al (1995) The receptor DEC-205 expressed by dendritic cells and thymic epithelial cells is involved in antigen processing. Nature 375: 151–155. 7753172 10.1038/375151a0

[27] Barral DC, Brenner MB (2007) CD1 antigen presentation: how it works. Nat Rev Immunol 7: 929–941. 18037897 10.1038/nri2191

[28] Simpson RJ, Jensen SS, Lim JW (2008) Proteomic profiling of exosomes: current perspectives. Proteomics 8: 4083–4099. 18780348 10.1002/pmic.200800109

[29] Uhlen M, Oksvold P, Fagerberg L, Lundberg E, Jonasson K, et al (2010) Towards a knowledge-based Human Protein Atlas. Nat Biotechnol 28: 1248–1250. 21139605 10.1038/nbt1210-1248

[30] Mittelbrunn M, Gutierrez-Vazquez C, Villarroya-Beltri C, Gonzalez S, Sanchez-Cabo F, et al (2011) Unidirectional transfer of microRNA-loaded exosomes from T cells to antigen-presenting cells. Nat Commun 2: 282. 21505438 10.1038/ncomms1285 PMC3104548

[31] Hedlund M, Stenqvist AC, Nagaeva O, Kjellberg L, Wulff M, et al (2009) Human placenta expresses and secretes NKG2D ligands via exosomes that down-modulate the cognate receptor expression: evidence for immunosuppressive function. J Immunol 183: 340–351. 19542445 10.4049/jimmunol.0803477

[32] Escola JM, Kleijmeer MJ, Stoorvogel W, Griffith JM, Yoshie O, et al (1998) Selective enrichment of tetraspan proteins on the internal vesicles of multivesicular endosomes and on exosomes secreted by human B-lymphocytes. J Biol Chem 273: 20121–20127. 9685355 10.1074/jbc.273.32.20121

[33] Admyre C, Grunewald J, Thyberg J, Gripenback S, Tornling G, et al (2003) Exosomes with major histocompatibility complex class II and co-stimulatory molecules are present in human BAL fluid. Eur Respir J 22: 578–583. 14582906 10.1183/09031936.03.00041703

[34] Aluwihare P, Mu Z, Zhao Z, Yu D, Weinreb PH, et al (2009) Mice that lack activity of alphavbeta6- and alphavbeta8-integrins reproduce the abnormalities of Tgfb1- and Tgfb3-null mice. J Cell Sci 122: 227–232. 19118215 10.1242/jcs.035246 PMC2714418

[35] Veron P, Segura E, Sugano G, Amigorena S, Thery C (2005) Accumulation of MFG-E8/lactadherin on exosomes from immature dendritic cells. Blood Cells Mol Dis 35: 81–88. 15982908 10.1016/j.bcmd.2005.05.001

[36] Segura E, Nicco C, Lombard B, Veron P, Raposo G, et al (2005) ICAM-1 on exosomes from mature dendritic cells is critical for efficient naive T-cell priming. Blood 106: 216–223. 15790784 10.1182/blood-2005-01-0220

[37] Segura E, Guerin C, Hogg N, Amigorena S, Thery C (2007) CD8+ dendritic cells use LFA-1 to capture MHC-peptide complexes from exosomes in vivo. J Immunol 179: 1489–1496. 17641014 10.4049/jimmunol.179.3.1489

[38] Blanchard N, Lankar D, Faure F, Regnault A, Dumont C, et al (2002) TCR activation of human T cells induces the production of exosomes bearing the TCR/CD3/zeta complex. J Immunol 168: 3235–3241. 11907077 10.4049/jimmunol.168.7.3235

[39] Petersen SH, Odintsova E, Haigh TA, Rickinson AB, Taylor GS, et al (2011) The role of tetraspanin CD63 in antigen presentation via MHC II. Eur J Immunol 41: 2556–2561. 21660937 10.1002/eji.201141438

[40] Nedjic J, Aichinger M, Emmerich J, Mizushima N, Klein L (2008) Autophagy in thymic epithelium shapes the T-cell repertoire and is essential for tolerance. Nature 455: 396–400. 18701890 10.1038/nature07208

[41] Kasai M, Tanida I, Ueno T, Kominami E, Seki S, et al (2009) Autophagic compartments gain access to the MHC class II compartments in thymic epithelium. J Immunol 183: 7278–7285. 19915056 10.4049/jimmunol.0804087

[42] Mathivanan S, Simpson RJ (2009) ExoCarta: A compendium of exosomal proteins and RNA. Proteomics 9: 4997–5000. 19810033 10.1002/pmic.200900351

[43] Mathivanan S, Lim JW, Tauro BJ, Ji H, Moritz RL, et al (2010) Proteomics analysis of A33 immunoaffinity-purified exosomes released from the human colon tumor cell line LIM1215 reveals a tissue-specific protein signature. Mol Cell Proteomics 9: 197–208. 19837982 10.1074/mcp.M900152-MCP200 PMC2830834

[44] Pisitkun T, Shen RF, Knepper MA (2004) Identification and proteomic profiling of exosomes in human urine. Proc Natl Acad Sci USA 101: 13368–13373. 15326289 10.1073/pnas.0403453101 PMC516573

[45] van Niel G, Raposo G, Candalh C, Boussac M, Hershberg R, et al (2001) Intestinal epithelial cells secrete exosome-like vesicles. Gastroenterology 121: 337–349. 11487543 10.1053/gast.2001.26263

[46] Kapsogeorgou EK, Abu-Helu RF, Moutsopoulos HM, Manoussakis MN (2005) Salivary gland epithelial cell exosomes: A source of autoantigenic ribonucleoproteins. Arthritis Rheum 52: 1517–1521. 15880835 10.1002/art.21005

[47] Milicevic Z, Milicevic NM, Laan M, Peterson P, Kisand K, et al (2010) Ultrastructure of medullary thymic epithelial cells of autoimmune regulator (Aire)-deficient mice. Immunol Cell Biol 88: 50–56. 19721455 10.1038/icb.2009.55

[48] Thery C, Amigorena S, Raposo G, Clayton A (2006) Isolation and characterization of exosomes from cell culture supernatants and biological fluids. Curr Protoc Cell Biol Chapter 3: Unit 3 22. 10.1002/0471143030.cb0322s30 18228490

[49] Shevchenko A, Wilm M, Vorm O, Mann M (1996) Mass spectrometric sequencing of proteins silver-stained polyacrylamide gels. Anal Chem 68: 850–858. 8779443 10.1021/ac950914h

[50] UniProt-Consortium (2012) Reorganizing the protein space at the Universal Protein Resource (UniProt). Nucleic Acids Res 40: D71–75. 22102590 10.1093/nar/gkr981 PMC3245120

