# Supplementary figures and images for: Characterization of Human Thymic Exosomes
Source: PLoS One. 2013 Jul 2;8(7):e67554. doi: 10.1371/journal.pone.0067554 (PMC3699640; doi:10.1371/journal.pone.0067554)

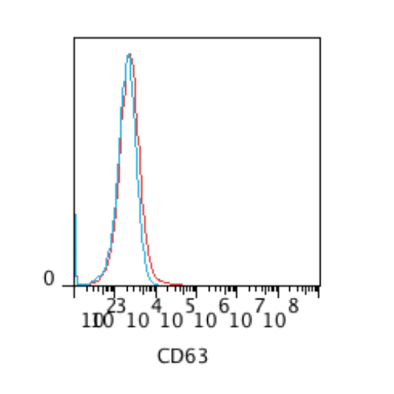

Supplement: Figure S1 — Flow cytometry staining for CD63. Red: (EVs+beads+antiCD63), blue: negative control (beads+antiCD63). (TIF) [file pone.0067554.s001.tif]
