# Supplementary material for: Characterization of Human Thymic Exosomes
Source: PLoS One. 2013 Jul 2;8(7):e67554. doi: 10.1371/journal.pone.0067554 (PMC3699640; doi:10.1371/journal.pone.0067554)
Supplement: Table S1 — All proteins found in the two thymic exosomal samples. (PDF) [file pone.0067554.s002.pdf]

Acc. Nr. (SwissProt)	Prot. Gr. (SwissProt)	Gene names
A0AVT1	A0AVT1	MOP4;UBA6;UBE1L2
A2RTX5	A2RTX5	TARSL2
A4FU69	A4FU69	EFCAB5
A5LHX3	A5LHX3	PSMB11
A5PLN9	A5PLN9	C5orf44
A5YKK6	A5YKK6	AD-005;CDC39;CNOT1;KIAA1007;NOT1
A6NDU8	A6NDU8	C5orf51
A6NHL2	A6NHL2	TUBAL3
A6NHR9	A6NHR9	KIAA0650;SMCHD1
B0I1T2	B0I1T2	MYO1G
O00139	O00139	KIF2;KIF2A;KNS2
O00148	O00148	DDX39
O00154	O00154	ACOT7;BACH
O00159	O00159	MYO1C
O00160	O00160	MYO1F
O00161	O00161	SNAP23
O00178	O00178	GTPBP1
O00186	O00186	STXBP3
O00231	O00231	PSMD11
O00232	O00232	PSMD12
O00233	O00233	PSMD9
O00255	O00255	MEN1;SCG2
O00264	O00264	HPR6.6;PGRMC;PGRMC1
O00299	O00299	CLIC1;NCC27
O00303	O00303	EIF3F;EIF3S5
O00411	O00411	POLRMT
O00422	O00422	GIG38;SAP18
O00429	O00429	DLP1;DNM1L;DRP1
O00443	O00443	PIK3C2A
O00471	O00471	EXOC5;SEC10;SEC10L1
O00487	O00487	POH1;PSMD14
O00522	O00522	CCM1;KRIT1
O00560	O00560	MDA9;SDCBP;SYCL
O00567	O00567	NOL5A;NOP56
O00629	O00629	KPNA4;QIP1
O00635	O00635	RNF15;RORET;TRIM38
O00750	O00750	PIK3C2B
O00764	O00764	C21orf124;C21orf97;PDXK;PKH;PNK;PRED79
O14579	O14579	COPE
O14638	O14638	ENPP3;PDNP3
O14672	O14672	ADAM10;KUZ;MADM
O14727	O14727	APAF1;KIAA0413
O14733	O14733	JNKK2;MAP2K7;MEK7;MKK7;PRKMK7
O14744	O14744	HRMT1L5;IBP72;JBP1;PRMT5;SKB1
O14745	O14745	NHERF;NHERF1;SLC9A3R1

O14772	O14772	FPGT;GFPP
O14773	O14773	CLN2;GIG1;TPP1;UNQ267/PRO304
O14787	O14787	TNPO2
O14802	O14802	POLR3A
O14828	O14828	C1orf3;PROPIN1;SCAMP3
O14879	O14879	IFI60;IFIT3;IFIT4
O14893	O14893	GEMIN2;SIP1
O14920	O14920	IKBKB;IKKB
O14929	O14929	HAT1
O14966	O14966	RAB7L1
O14972	O14972	DCRA;DSCR3;DSCRA
O14976	O14976	GAK
O14979	O14979	HNRPDL;JKTBP
O14980	O14980	CRM1;XPO1
O14981	O14981	BTAF1;TAF172
O15054	O15054	JMJD3;KDM6B;KIAA0346
O15111	O15111	CHUK;IKKA;TCF16
O15117	O15117	FYB;SLAP130
O15126	O15126	SCAMP;SCAMP1
O15127	O15127	SCAMP2
O15143	O15143	ARC41;ARPC1B
O15144	O15144	ARC34;ARPC2;PRO2446
O15145	O15145	ARC21;ARPC3
O15160	O15160	POLR1C;POLR1E
O15173	O15173	DG6;PGRMC2;PMBP
O15230	O15230	KIAA0533;KIAA1907;LAMA5
O15231	O15231	ZNF185
O15247	O15247	CLIC2
O15258	O15258	RER1
O15294	O15294	OGT
O15305	O15305	PMM2
O15371	O15371	EIF3D;EIF3S7
O15372	O15372	EIF3H;EIF3S3
O15397	O15397	IPO8;RANBP8
O15400	O15400	STX7
O15511	O15511	ARC16;ARPC5
O15533	O15533	NGS17;TAPA;TAPBP
O43149	O43149	KIAA0399;ZZEF1
O43156	O43156	KIAA0406
O43172	O43172	PRP4;PRPF4
O43175	O43175	PGDH3;PHGDH
O43237	O43237	DNCLI2;DYNC1LI2;LIC2
O43242	O43242	PSMD3
O43252	O43252	ATPSK1;PAPSS;PAPSS1
O43264	O43264	ZW10
O43299	O43299	KIAA0415

O43301	O43301	HSPA12A;KIAA0417
O43314	O43314	HISPPD1;KIAA0433;PPIP5K2;VIP2
O43324	O43324	AIMP3;EEF1E1;P18
O43390	O43390	HNRNPR;HNRPR
O43396	O43396	TRP32;TXL;TXNL;TXNL1
O43447	O43447	CYP20;CYPH;PPIH
O43491	O43491	EPB41L2
O43493	O43493	TGN46;TGN51;TGOLN2
O43561	O43561	LAT
O43592	O43592	XPOT
O43617	O43617	BET3;CDABP0066;TRAPPC3
O43665	O43665	RGS10
O43681	O43681	ARSA;ASNA1;TRC40
O43684	O43684	BUB3
O43747	O43747	ADTG;AP1G1;CLAPG1
O43752	O43752	STX6
O43760	O43760	SYNGR2;UNQ352/PRO615
O43765	O43765	SGT;SGT1;SGTA
O43776	O43776	ASNS;NARS
O43813	O43813	GPR69A;LANCL1
O43865	O43865	AHCYL1;DCAL;XPVKONA
O60231	O60231	DBP2;DDX16;DHX16;KIAA0577
O60256	O60256	PRPSAP2
O60306	O60306	AQR;KIAA0560
O60341	O60341	AOF2;KDM1;KDM1A;KIAA0601;LSD1
O60449	O60449	CD205;CLEC13B;LY75
O60496	O60496	DOK2
O60499	O60499	STX10;SYN10
O60506	O60506	HNRPQ;NSAP1;SYNCRIP
O60547	O60547	GMDS
O60645	O60645	EXOC3;SEC6;SEC6L1
O60701	O60701	UGDH
O60716	O60716	CTNND1;KIAA0384
O60749	O60749	SNX2;TRG9
O60763	O60763	USO1;VDP
O60826	O60826	CCDC22;CXorf37;JM1
O60880	O60880	DSHP;SAP;SH2D1A
O60884	O60884	CPR3;DNAJA2;HIRIP4
O60911	O60911	CATL2;CTSL2;CTSU;CTSV;UNQ268/PRO305
O60942	O60942	CAP1A;RNGTT
O75083	O75083	WDR1
O75116	O75116	KIAA0619;ROCK2
O75150	O75150	BRE1B;KIAA0661;RNF40
O75153	O75153	KIAA0664
O75165	O75165	DNAJC13;KIAA0678;RME8
O75191	O75191	XYLB

O75351	O75351	MIG1;SKD1;VPS42;VPS4B
O75367	O75367	H2AFY;MACROH2A1
O75368	O75368	SH3BGRL
O75369	O75369	FLN1L;FLN3;FLNB;TABP;TAP
O75396	O75396	SEC22B;SEC22L1
O75436	O75436	VPS26;VPS26A
O75475	O75475	DFS70;LEDGF;PSIP1;PSIP2
O75531	O75531	BAF;BANF1;BCRG1
O75533	O75533	SAP155;SF3B1
O75534	O75534	CSDE1;D1S155E;KIAA0885;NRU;UNR
O75569	O75569	HSD14;HSD-14;PACT;PRKRA;RAX
O75582	O75582	MSK1;RPS6KA5
O75592	O75592	KIAA0916;MYCBP2;PAM
O75607	O75607	NPM3
O75608	O75608	APT1;LPL1;LYPLA1
O75643	O75643	ASCC3L1;HELIC2;KIAA0788;SNRNP200
O75676	O75676	MSK2;RPS6KA4
O75695	O75695	RP2
O75787	O75787	ATP6AP2;ATP6IP2;CAPER;ELDF10;HT028
O75792	O75792	RNASEH2A;RNASEHI;RNHIA
O75828	O75828	CBR3
O75832	O75832	PSMD10
O75915	O75915	ARL6IP5;DERP11;HSPC127;JWA;PRA2;PRAF3
O75934	O75934	BCAS2;DAM1
O75935	O75935	DCTN22;DCTN3
O75955	O75955	FLOT1
O76021	O76021	CATX11;CSIG;L12;PBK1;RSL1D1
O76094	O76094	SRP72
O94768	O94768	DRAK2;STK17B
O94819	O94819	CMLAP;KBTBD11;KIAA0711;KLHDC7C
O94822	O94822	C21orf10;C21orf98;HSPC087;KIAA0714
O94832	O94832	KIAA0727;MYO1D
O94874	O94874	KIAA0776;UFL1
O94906	O94906	C20orf14;PRPF6
O94973	O94973	ADTAB;AP2A2;CLAPA2;HIP9;HYPJ;KIAA0899
O95155	O95155	HDNB1;KIAA0684;UBE4B;UFD2
O95163	O95163	ELP1;IKAP;IKBKAP
O95248	O95248	MTMR5;SBF1
O95249	O95249	GOSR1;GS28
O95292	O95292	UNQ484/PRO983;VAPB
O95319	O95319	BRUNOL3;CELF2;CUGBP2;ETR3;NAPOR
O95336	O95336	PGLS
O95340	O95340	ATPSK2;PAPSS2
O95347	O95347	CAPE;PRO0324;SMC2;SMC2L1
O95352	O95352	APG7L;ATG7
O95372	O95372	APT2;LYPLA2

O95396	O95396	MOCS3;UBA4
O95399	O95399	UNQ525/PRO1068;UTS2
O95400	O95400	CD2BP2;KIAA1178
O95456	O95456	C21LRP;DSCR2;PAC1;PSMG1
O95466	O95466	C17orf1;C17orf1B;FMNL;FMNL1
O95487	O95487	SEC24B
O95571	O95571	ETHE1;HSCO
O95671	O95671	ASMTL
O95721	O95721	SNAP29
O95747	O95747	KIAA1101;OSR1;OXSR1
O95757	O95757	APG1;HSPA4L;OSP94
O95782	O95782	ADTAA;AP2A1;CLAPA1
O95786	O95786	DDX58
O95832	O95832	CLD1;CLDN1;SEMP1;UNQ481/PRO944
O95865	O95865	DDAH;DDAH2;G6A;NG30
P00338	P00338	LDHA;PIG19
P00352	P00352	ALDC;ALDH1;ALDH1A1;PUMB1
P00387	P00387	CYB5R3;DIA1
P00390	P00390	GLUR;GRD1;GSR
P00441	P00441	SOD1
P00450	P00450	CP
P00488	P00488	F13A;F13A1
P00558	P00558	MIG10;OK/SW-cl.110;PGK1;PGKA
P00813	P00813	ADA
P01008	P01008	AT3;PRO0309;SERPINC1
P01009	P01009	AAT;PI;PRO0684;PRO2209;SERPINA1
P01011	P01011	AACT;GIG24;GIG25;SERPINA3
P01024	P01024	C3;CPAMD1
P01116	P01116	KRAS;KRAS2;RASK2
P01591	P01591	IGCJ;IGJ
P01621	P01621	
P01625	P01625	
P01714	P01714	
P01717	P01717	
P01730	P01730	CD4
P01732	P01732	CD8A;MAL
P01764	P01764	
P01781	P01781	
P01834	P01834	IGKC
P01848	P01848	TCRA;TRAC
P01871	P01871	IGHM
P01920	P01920	HLA-DQB;HLA-DQB1
P02452	P02452	COL1A1
P02489	P02489	CRYA1;CRYAA;HSPB4
P02511	P02511	CRYA2;CRYAB
P02647	P02647	APOA1

P02671	P02671	FGA
P02679	P02679	FGG;PRO2061
P02749	P02749	APOH;B2G1
P02751	P02751	FN;FN1
P02786	P02786	TFRC
P02787	P02787	PRO1400;TF
P02788	P02788	LF;LTF
P02790	P02790	HPX
P02792	P02792	FTL
P02794	P02794	FTH;FTH1;FTHL6;OK/SW-cl.84;PIG15
P04004	P04004	VTN
P04040	P04040	CAT
P04049	P04049	RAF;RAF1
P04053	P04053	DNTT;TDT
P04075	P04075	ALDA;ALDOA
P04080	P04080	CST6;CSTB;STFB
P04083	P04083	ANX1;ANXA1;LPC1
P04150	P04150	GRL;NR3C1
P04216	P04216	THY1
P04220	P04220	
P04233	P04233	CD74;DHLAG
P04234	P04234	CD3D;T3D
P04275	P04275	F8VWF;VWF
P04350	P04350	TUBB4;TUBB5
P04433	P04433	
P04440	P04440	HLA-DP1B;HLA-DPB1
P04632	P04632	CAPN4;CAPNS;CAPNS1
P04792	P04792	HSP27;HSP28;HSPB1
P04839	P04839	CYBB;NOX2
P04843	P04843	RPN1
P04844	P04844	RPN2
P05026	P05026	ATP1B;ATP1B1
P05164	P05164	MPO
P05198	P05198	EIF2A;EIF2S1
P05362	P05362	ICAM1
P05386	P05386	RPLP1;RRP1
P05387	P05387	D11S2243E;RPLP2;RPP2
P06126	P06126	CD1A
P06127	P06127	CD5;LEU1
P06396	P06396	GSN
P06454	P06454	PTMA;TMSA
P06703	P06703	CACY;S100A6
P06729	P06729	CD2;SRBC
P06730	P06730	EIF4E;EIF4EL1;EIF4F
P06746	P06746	POLB
P06748	P06748	NPM;NPM1

P06756	P06756	ITGAV;MSK8;VNRA
P07099	P07099	EPHX;EPHX1;EPOX
P07195	P07195	LDHB
P07203	P07203	GPX1
P07205	P07205	PGK2;PGKB
P07237	P07237	ERBA2L;P4HB;PDI;PDIA1;PO4DB
P07305	P07305	H1F0;H1FV
P07332	P07332	FES;FPS
P07339	P07339	CPSD;CTSD
P07384	P07384	CANPL1;CAPN1;PIG30
P07437	P07437	OK/SW-cl.56;TUBB;TUBB5
P07741	P07741	APRT
P07766	P07766	CD3E;T3E
P07814	P07814	EPRS;GLNS;PARS;PIG32;QARS;QPRS
P07858	P07858	CPSB;CTSB
P07947	P07947	YES;YES1
P07948	P07948	LYN
P08195	P08195	MDU1;SLC3A2
P08237	P08237	PFKM;PFKX
P08246	P08246	ELA2;ELANE
P08311	P08311	CTSG
P08473	P08473	EPN;MME
P08575	P08575	CD45;PTPRC
P08579	P08579	SNRPB2
P08621	P08621	RNPU1Z;RPU1;SNRNP70;SNRP70;U1AP1
P08631	P08631	HCK
P08648	P08648	FNRA;ITGA5
P08708	P08708	RPS17
P08754	P08754	GNAI3
P08758	P08758	ANX5;ANXA5;ENX2;PP4
P08865	P08865	LAMBR;LAMR1;RPSA
P08962	P08962	CD63;MLA1;TSPAN30
P09086	P09086	OCT2;OTF2;POU2F2
P09211	P09211	FAEES3;GST3;GSTP1
P09326	P09326	BCM1;BLAST1;CD48
P09382	P09382	LGALS1
P09417	P09417	DHPR;QDPR
P09496	P09496	CLTA
P09497	P09497	CLTB
P09525	P09525	ANX4;ANXA4
P09543	P09543	CNP
P09661	P09661	SNRPA1
P09693	P09693	CD3G;T3G
P09874	P09874	ADPRT;PARP1;PPOL
P09972	P09972	ALDC;ALDOC
P0C2W1	P0C2W1	FBX45;FBXO45

P10114	P10114	RAP2A
P10155	P10155	RO60;SSA2;TROVE2
P10301	P10301	RRAS
P10599	P10599	TRDX;TRX;TRX1;TXN
P10768	P10768	ESD
P10909	P10909	AAG4;APOJ;CLI;CLU;KUB1
P11021	P11021	GRP78;HSPA5
P11142	P11142	HSC70;HSP73;HSPA10;HSPA8
P11171	P11171	E41P;EPB41
P11172	P11172	OK/SW-cl.21;UMPS
P11215	P11215	CD11B;CR3A;ITGAM
P11217	P11217	PYGM
P11233	P11233	RAL;RALA
P11234	P11234	RALB
P11279	P11279	LAMP1
P11388	P11388	TOP2;TOP2A
P11413	P11413	G6PD
P11488	P11488	GNAT1;GNATR
P11586	P11586	MTHFC;MTHFD;MTHFD1
P11678	P11678	EPER;EPO;EPP;EPX
P11717	P11717	IGF2R;MPRI
P11766	P11766	ADH5;ADHX;FDH
P11836	P11836	CD20;MS4A1
P11908	P11908	PRPS2
P12004	P12004	PCNA
P12268	P12268	IMPD2;IMPDH2
P12429	P12429	ANX3;ANXA3
P12883	P12883	MYH7;MYHCB
P12956	P12956	G22P1;XRCC6
P13010	P13010	G22P2;XRCC5
P13473	P13473	LAMP2
P13489	P13489	PRI;RNH;RNH1
P13612	P13612	CD49D;ITGA4
P13639	P13639	EEF2;EF2
P13667	P13667	ERP70;ERP72;PDIA4
P13746	P13746	HLAA;HLA-A
P13804	P13804	ETFA
P14209	P14209	CD99;MIC2;MIC2X;MIC2Y
P14324	P14324	FDPS;FPS;KIAA1293
P14866	P14866	HNRNPL;HNRPL;P/OKcl.14
P14868	P14868	DARS;PIG40
P14923	P14923	CTNNG;DP3;JUP
P15144	P15144	ANPEP;APN;CD13;PEPN
P15311	P15311	EZR;VIL2
P15880	P15880	RPS2;RPS4
P15924	P15924	DSP

P15927	P15927	REPA2;RPA2;RPA32;RPA34
P16070	P16070	CD44;LHR;MDU2;MDU3;MIC4
P16150	P16150	CD43;SPN
P16152	P16152	CBR;CBR1;CRN
P16278	P16278	ELNR1;GLB1
P16284	P16284	PECAM1
P16401	P16401	H1F5;HIST1H1B
P16402	P16402	H1F3;HIST1H1D
P16403	P16403	H1F2;HIST1H1C
P16422	P16422	EPCAM;GA733-2;M1S2;M4S1;MIC18;TACSTD1
P16455	P16455	MGMT
P17301	P17301	CD49B;ITGA2
P17612	P17612	PKACA;PRKACA
P17655	P17655	CANPL2;CAPN2
P17706	P17706	PTPN2;PTPT
P17812	P17812	CTPS
P17844	P17844	DDX5;G17P1;HELR;HLR1
P17858	P17858	PFKL
P17980	P17980	PSMC3;TBP1
P17987	P17987	CCT1;CCTA;TCP1
P18031	P18031	PTP1B;PTPN1
P18074	P18074	ERCC2;XPD;XPDC
P18077	P18077	GIG33;RPL35A
P18085	P18085	ARF2;ARF4
P18124	P18124	RPL7
P18206	P18206	VCL
P18433	P18433	PTPA;PTPRA;PTPRL2
P18583	P18583	C21orf50;DBP5;HSPC310;HSPC312;KIAA1019
P18621	P18621	RPL17
P18754	P18754	CHC1;RCC1
P19174	P19174	PLC1;PLCG1
P19338	P19338	NCL
P19387	P19387	A-152E5.7;POLR2C
P19388	P19388	POLR2E
P19474	P19474	RNF81;RO52;SSA1;TRIM21
P19525	P19525	EIF2AK2;PKR;PRKR
P19623	P19623	SPS1;SRM;SRML1
P19784	P19784	CK2A2;CSNK2A2
P19878	P19878	NCF2;NOXA2;P67PHOX
P19971	P19971	ECGF1;TYMP
P20039	P20039	HLA-DRB1
P20073	P20073	ANX7;ANXA7;OK/SW-cl.95;SNX
P20339	P20339	RAB5;RAB5A
P20585	P20585	DUC1;DUG;MSH3
P20591	P20591	MX1
P20592	P20592	MX2

P20618	P20618	PSC5;PSMB1
P20645	P20645	M6PR;MPR46;MPRD
P20701	P20701	CD11A;ITGAL
P20839	P20839	IMPD1;IMPDH1
P20963	P20963	CD247;CD3Z;T3Z;TCRZ
P21266	P21266	GST5;GSTM3
P21359	P21359	NF1
P21589	P21589	NT5;NT5E;NTE
P21796	P21796	VDAC;VDAC1
P21926	P21926	CD9;GIG2;MIC3;TSPAN29
P21964	P21964	COMT
P21980	P21980	TGM2
P22102	P22102	GART;PGFT;PRGS
P22234	P22234	ADE2;AIRC;PAICS;PAIS
P22314	P22314	A1S9T;UBA1;UBE1
P22626	P22626	HNRNPA2B1;HNRPA2B1
P22694	P22694	PRKACB
P23229	P23229	ITGA6
P23246	P23246	PSF;SFPQ
P23284	P23284	CYPB;PPIB
P23381	P23381	IFI53;WARS;WRS
P23396	P23396	OK/SW-cl.26;RPS3
P23526	P23526	AHCY;SAHH
P23528	P23528	CFL;CFL1
P23921	P23921	RR1;RRM1
P23942	P23942	PRPH;PRPH2;RDS;TSPAN22
P24534	P24534	EEF1B;EEF1B2;EF1B
P24666	P24666	ACP1
P24928	P24928	POLR2;POLR2A
P24941	P24941	CDK2
P25205	P25205	MCM3
P25325	P25325	MPST;TST2
P25445	P25445	APT1;FAS;FAS1;TNFRSF6
P25685	P25685	DNAJ1;DNAJB1;HDJ1;HSPF1
P25705	P25705	ATP5A;ATP5A1;ATP5AL2;ATPM
P25786	P25786	HC2;NU;PROS30;PSC2;PSMA1
P25787	P25787	HC3;PSC3;PSMA2
P25788	P25788	HC8;PSC8;PSMA3
P25789	P25789	HC9;PSC9;PSMA4
P26038	P26038	MSN
P26196	P26196	DDX6;HLR2;RCK
P26232	P26232	CAPR;CTNNA2
P26373	P26373	BBC1;OK/SW-cl.46;RPL13
P26599	P26599	PTB;PTBP1
P26639	P26639	TARS
P26640	P26640	G7A;VARS;VARS2

P26641	P26641	EEF1G;EF1G;PRO1608
P27105	P27105	BND7;EPB72;STOM
P27348	P27348	YWHAQ
P27482	P27482	CALML3
P27487	P27487	ADCP2;CD26;DPP4
P27694	P27694	REPA1;RPA1;RPA70
P27701	P27701	CD82;KAI1;SAR2;ST6;TSPAN27
P27707	P27707	DCK
P27708	P27708	CAD
P27797	P27797	CALR;CRTC
P27824	P27824	CANX
P28062	P28062	LMP7;PSMB5i;PSMB8;RING10;Y2
P28065	P28065	LMP2;PSMB6i;PSMB9;RING12
P28066	P28066	PSMA5
P28070	P28070	PROS26;PSMB4
P28072	P28072	LMPY;PSMB6;Y
P28074	P28074	LMPX;MB1;PSMB5;X
P28340	P28340	POLD;POLD1
P28482	P28482	ERK2;MAPK1;PRKM1;PRKM2
P28838	P28838	LAP3;LAPEP;PEPS
P28907	P28907	CD38
P29016	P29016	CD1B
P29017	P29017	CD1C
P29144	P29144	TPP2
P29350	P29350	HCP;PTP1C;PTPN6
P29401	P29401	TKT
P29692	P29692	EEF1D;EF1D
P29728	P29728	OAS2
P29966	P29966	MACS;MARCKS;PRKCSL
P29992	P29992	GA11;GNA11
P30040	P30040	C12orf8;ERP28;ERP29
P30041	P30041	AOP2;KIAA0106;PRDX6
P30043	P30043	BLVRB;FLR
P30050	P30050	RPL12
P30086	P30086	PBP;PEBP;PEBP1
P30101	P30101	ERP57;ERP60;GRP58;PDIA3
P30153	P30153	PPP2R1A
P30154	P30154	PPP2R1B
P30203	P30203	CD6
P30466	P30466	HLAB;HLA-B
P30519	P30519	HMOX2;HO2
P30566	P30566	ADSL;AMPS
P30626	P30626	SRI
P30679	P30679	GNA15;GNA16
P30876	P30876	POLR2B
P31150	P31150	GDI1;GDIL;OPHN2;RABGDIA;XAP4

P31153	P31153	AMS2;MAT2A;MATA2
P31689	P31689	DNAJ2;DNAJA1;HDJ2;HSJ2;HSPF4
P31939	P31939	ATIC;OK/SW-cl.86;PURH
P31942	P31942	HNRNPH3;HNRPH3
P31943	P31943	HNRNPH1;HNRPH;HNRPH1
P31946	P31946	YWHAB
P31947	P31947	HME1;SFN
P31949	P31949	MLN70;S100A11;S100C
P32019	P32019	INPP5B
P32119	P32119	NKEFB;PRDX2;TDPX1
P32455	P32455	GBP1
P32942	P32942	ICAM3
P32969	P32969	OK/SW-cl.103;RPL9;RPL9P7;RPL9P8;RPL9P9
P33991	P33991	CDC21;MCM4
P33992	P33992	CDC46;MCM5
P33993	P33993	CDC47;MCM2;MCM7
P34896	P34896	SHMT1
P34897	P34897	SHMT2
P34913	P34913	EPHX2
P34932	P34932	APG2;HSPA4
P34947	P34947	GPRK5;GRK5
P35030	P35030	PRSS3;PRSS4;TRY3;TRY4
P35080	P35080	PFN2
P35221	P35221	CTNNA1
P35236	P35236	PTPN7
P35241	P35241	RDX
P35249	P35249	RFC4
P35250	P35250	RFC2
P35268	P35268	RPL22
P35270	P35270	SPR
P35580	P35580	MYH10
P35606	P35606	COPB2
P35611	P35611	ADD1;ADDA
P35613	P35613	BSG;UNQ6505/PRO21383
P35659	P35659	DEK
P35754	P35754	GLRX;GRX
P35998	P35998	MSS1;PSMC2
P36405	P36405	ARFL3;ARL3
P36507	P36507	MAP2K2;MEK2;MKK2;PRKMK2
P36578	P36578	RPL1;RPL4
P36873	P36873	PPP1CC
P36969	P36969	GPX4
P37108	P37108	SRP14
P37837	P37837	TAL;TALDO;TALDO1;TALDOR
P38606	P38606	ATP6A1;ATP6V1A;ATP6V1A1;VPP2
P38919	P38919	DDX48;EIF4A3;KIAA0111

P39023	P39023	OK/SW-cl.32;RPL3
P39656	P39656	DDOST;KIAA0115;OK/SW-cl.45;OST48
P39748	P39748	FEN1;RAD2
P40121	P40121	AFCP;CAPG;MCP
P40306	P40306	LMP10;MECL1;PSMB10
P40616	P40616	ARL1
P40692	P40692	COCA2;MLH1
P40763	P40763	APRF;STAT3
P40925	P40925	MDH1;MDHA
P40937	P40937	RFC5
P40938	P40938	RFC3
P41214	P41214	HCA56;LGTN
P41217	P41217	CD200;MOX1;MOX2;My033
P41218	P41218	MNDA
P41226	P41226	UBA7;UBE1L;UBE2
P41250	P41250	GARS
P41252	P41252	IARS
P42025	P42025	ACTR1B;CTRN2
P42224	P42224	STAT1
P42226	P42226	STAT6
P42338	P42338	PIK3C1;PIK3CB
P42345	P42345	FRAP;FRAP1;FRAP2;MTOR
P42566	P42566	AF1P;EPS15
P42574	P42574	CASP3;CPP32
P42677	P42677	MPS1;RPS27
P42858	P42858	HD;HTT;IT15
P43007	P43007	ASCT1;SATT;SLC1A4
P43034	P43034	LIS1;MDCR;MDS;PAFAH1B1;PAFAHA
P43243	P43243	KIAA0723;MATR3
P43246	P43246	MSH2
P43378	P43378	PTPN9
P43403	P43403	SRK;ZAP70
P43405	P43405	SYK
P43487	P43487	RANBP1
P43490	P43490	NAMPT;PBEF;PBEF1
P43686	P43686	MIP224;PSMC4;TBP7
P45973	P45973	CBX5;HP1A
P45974	P45974	ISOT;USP5
P45985	P45985	JNKK1;MAP2K4;MEK4;MKK4;PRKMK4;SERK1
P46019	P46019	PHKA2;PHKLA;PYK
P46020	P46020	PHKA;PHKA1
P46060	P46060	KIAA1835;RANGAP1;SD
P46063	P46063	RECQ1;RECQL;RECQL1
P46087	P46087	NOL1;NOP2
P46439	P46439	GSTM5
P46459	P46459	NSF

P46734	P46734	MAP2K3;MEK3;MKK3;PRKMK3
P46776	P46776	RPL27A
P46777	P46777	MSTP030;RPL5
P46778	P46778	RPL21
P46779	P46779	RPL28
P46781	P46781	RPS9
P46782	P46782	RPS5
P47755	P47755	CAPZA2
P47756	P47756	CAPZB
P47897	P47897	QARS
P47929	P47929	LGALS7;LGALS7B;PIG1
P48059	P48059	LIMS1;PINCH;PINCH1
P48147	P48147	PEP;PREP
P48426	P48426	PIP4K2A;PIP5K2;PIP5K2A
P48444	P48444	ARCN1;COPD
P48507	P48507	GCLM;GLCLR
P48553	P48553	EHOC1;TMEM1;TRAPPC10
P48556	P48556	PSMD8
P48643	P48643	CCT5;CCTE;KIAA0098
P48960	P48960	CD97
P49005	P49005	POLD2
P49006	P49006	MARCKSL1;MLP;MRP
P49137	P49137	MAPKAPK2
P49189	P49189	ALDH4;ALDH7;ALDH9;ALDH9A1
P49207	P49207	RPL34
P49247	P49247	RPI;RPIA
P49257	P49257	ERGIC53;F5F8D;LMAN1
P49327	P49327	FAS;FASN
P49368	P49368	CCT3;CCTG;TRIC5
P49419	P49419	ALDH7A1;ATQ1
P49441	P49441	INPP1
P49588	P49588	AARS
P49591	P49591	SARS;SERS
P49720	P49720	PSMB3
P49721	P49721	PSMB2
P49736	P49736	BM28;CCNL1;CDCL1;KIAA0030;MCM2
P49754	P49754	VPS41
P49755	P49755	TMED10;TMP21
P49770	P49770	EIF2B2;EIF2BB
P49840	P49840	GSK3A
P49841	P49841	GSK3B
P49915	P49915	GMPS
P49916	P49916	LIG3
P49959	P49959	HNGS1;MRE11;MRE11A
P50151	P50151	GNG10;GNGT10
P50224	P50224	STM;SULT1A3;SULT1A4

P50395	P50395	GDI2;RABGDIB
P50454	P50454	CBP1;CBP2;HSP47;PIG14;SERPINH1;SERPINH2
P50552	P50552	VASP
P50750	P50750	CDC2L4;CDK9
P50851	P50851	BGL;CDC4L;LBA;LRBA
P50914	P50914	RPL14
P50990	P50990	C21orf112;CCT8;CCTQ;KIAA0002
P50991	P50991	CCT4;CCTD;SRB
P51148	P51148	RAB5C;RABL
P51149	P51149	RAB7;RAB7A
P51153	P51153	GIG4;RAB13
P51159	P51159	RAB27;RAB27A
P51570	P51570	GALK;GALK1
P51572	P51572	BAP31;BCAP31;DXS1357E
P51608	P51608	MECP2
P51610	P51610	HCF1;HCFC1;HFC1
P51665	P51665	MOV34L;PSMD7
P51784	P51784	UHX1;USP11
P51809	P51809	SYBL1;VAMP7
P51991	P51991	HNRNPA3;HNRPA3
P52209	P52209	PGD;PGDH
P52272	P52272	HNRNPM;HNRPM;NAGR1
P52294	P52294	KPNA1;RCH2
P52298	P52298	CBP20;NCBP2;PIG55
P52333	P52333	JAK3
P52434	P52434	POLR2H
P52564	P52564	MAP2K6;MEK6;MKK6;PRKMK6
P52565	P52565	ARHGDIA;GDIA1
P52566	P52566	ARHGDIB;GDIA2;GDID4;RAP1GN1
P52597	P52597	HNRNPF;HNRPF
P52630	P52630	STAT2
P52701	P52701	GTBP;MSH6
P52907	P52907	CAPZA1
P53004	P53004	BLVR;BLVRA;BVR
P53396	P53396	ACLY
P53618	P53618	COPB;COPB1;MSTP026
P53621	P53621	COPA
P53680	P53680	AP17;AP2S1;CLAPS2
P53999	P53999	PC4;RPO2TC1;SUB1
P54105	P54105	CLCI;CLNS1A;ICLN
P54132	P54132	BLM;RECQ2;RECQL3
P54136	P54136	RARS
P54577	P54577	YARS
P54652	P54652	HSPA2
P54709	P54709	ATP1B3
P54710	P54710	ATP1C;ATP1G1;FXYD2

P54727	P54727	RAD23B
P55039	P55039	DRG2
P55060	P55060	CAS;CSE1L;XPO2
P55160	P55160	HEM1;NCKAP1L
P55209	P55209	NAP1L1;NRP
P55212	P55212	CASP6;MCH2
P55263	P55263	ADK
P55265	P55265	ADAR;ADAR1;DSRAD;G1P1;IFI4
P55735	P55735	D3S1231E;SEC13;SEC13L1;SEC13R
P55769	P55769	NHP2L1
P55795	P55795	FTP3;HNRNPH2;HNRPH2
P55884	P55884	EIF3B;EIF3S9
P56192	P56192	MARS
P56211	P56211	ARPP19
P56537	P56537	EIF3A;EIF6;ITGB4BP;OK/SW-cl.27
P56589	P56589	PEX3
P57678	P57678	GEMIN4
P57735	P57735	CATX8;RAB25
P57737	P57737	CORO7
P57764	P57764	DFNA5L;FKSG10;GSDMD;GSDMDC1
P57772	P57772	EEFSEC;SELB
P59768	P59768	GNG2
P59998	P59998	ARC20;ARPC4
P60033	P60033	CD81;TAPA1;TSPAN28
P60174	P60174	TPI;TPI1
P60228	P60228	EIF3E;EIF3S6;INT6
P60842	P60842	DDX2A;EIF4A;EIF4A1
P60866	P60866	RPS20
P60900	P60900	PROS27;PSMA6
P60953	P60953	CDC42
P60981	P60981	ACTDP;DSN;DSTN
P61006	P61006	MEL;RAB8;RAB8A
P61011	P61011	SRP54
P61020	P61020	RAB5B
P61026	P61026	RAB10
P61081	P61081	UBC12;UBE2M
P61106	P61106	RAB14
P61160	P61160	ACTR2;ARP2
P61163	P61163	ACTR1A;CTRN1
P61221	P61221	ABCE1;OK/SW-cl.40
P61225	P61225	RAP2B
P61247	P61247	FTE1;MFTL;RPS3A
P61289	P61289	PSME3
P61313	P61313	EC45;RPL15;TCBAP0781
P61353	P61353	RPL27
P61421	P61421	ATP6D;ATP6V0D1;VPATPD

P61769	P61769	B2M;CDABP0092;HDCMA22P
P61803	P61803	DAD1
P61923	P61923	CGI-120;COPZ;COPZ1;HSPC181
P61962	P61962	DCAF7;HAN11;WDR68
P61964	P61964	BIG3;WDR5
P61978	P61978	HNRNPK;HNRPK
P61981	P61981	YWHAG
P62070	P62070	RRAS2;TC21
P62081	P62081	RPS7
P62136	P62136	PPP1A;PPP1CA
P62140	P62140	PPP1CB
P62158	P62158	CALM;CALM1;CALM2
P62191	P62191	PSMC1
P62195	P62195	PSMC5;SUG1
P62241	P62241	OK/SW-cl.83;RPS8
P62244	P62244	OK/SW-cl.82;RPS15A
P62249	P62249	RPS16
P62258	P62258	YWHAE
P62263	P62263	PRO2640;RPS14
P62266	P62266	RPS23
P62269	P62269	D6S218E;RPS18
P62277	P62277	RPS13
P62280	P62280	RPS11
P62306	P62306	PBSCF;SNRPF
P62312	P62312	LSM6
P62318	P62318	SNRPD3
P62333	P62333	PSMC6;SUG2
P62424	P62424	RPL7A;SURF3;SURF-3
P62701	P62701	CCG2;RPS4;RPS4X;SCAR
P62714	P62714	PPP2CB
P62753	P62753	OK/SW-cl.2;RPS6
P62805	P62805	H4/A;H4/B;H4/C;H4/D;H4/E
P62826	P62826	ARA24;OK/SW-cl.81;RAN
P62829	P62829	RPL23
P62834	P62834	KREV1;RAP1A
P62847	P62847	RPS24
P62851	P62851	RPS25
P62857	P62857	RPS28
P62875	P62875	POLR2L
P62877	P62877	RBX1;RNF75;ROC1
P62888	P62888	RPL30
P62899	P62899	RPL31
P62906	P62906	NEDD6;RPL10A
P62910	P62910	PP9932;RPL32
P62913	P62913	RPL11
P62917	P62917	RPL8

P62937	P62937	CYPA;PPIA
P62942	P62942	FKBP1;FKBP12;FKBP1A
P62993	P62993	ASH;GRB2
P63000	P63000	MIG5;RAC1;TC25
P63010	P63010	ADTB2;AP2B1;CLAPB1
P63027	P63027	SYB2;VAMP2
P63096	P63096	GNAI1
P63104	P63104	YWHAZ
P63172	P63172	DYNLT1;TCTEL1;TCTEX1;TCTEX-1
P63208	P63208	EMC19;OCP2;SKP1;SKP1A;TCEB1L
P63218	P63218	GNG5;GNGT5
P63220	P63220	RPS21
P63244	P63244	GNB2L1;HLC7;PIG21
P67775	P67775	PPP2CA
P67870	P67870	CK2N;CSNK2B;G5A
P68366	P68366	TUBA1;TUBA4A
P68400	P68400	CK2A1;CSNK2A1
P68402	P68402	PAFAH1B2;PAFAHB
P69891	P69891	HBG1;PRO2979
P69892	P69892	HBG2
P69905	P69905	HBA1;HBA2
P78346	P78346	RNASEP2;RPP30
P78356	P78356	PIP4K2B;PIP5K2B
P78371	P78371	99D8.1;CCT2;CCTB
P78417	P78417	GSTO1;GSTTLP28
P78527	P78527	HYRC;HYRC1;PRKDC
P80723	P80723	BASP1;NAP22
P80748	P80748	
P82094	P82094	ARA160;TMF1
P83436	P83436	COG7;UNQ3082/PRO10013
P83731	P83731	RPL24
P83916	P83916	CBX;CBX1
P84085	P84085	ARF5
P84095	P84095	ARHG;RHOG
P84098	P84098	RPL19
P84103	P84103	SFRS3;SRP20
P98095	P98095	FBLN2
P98194	P98194	ATP2C1;HUSSY-28;KIAA1347;PMR1L
Q00005	Q00005	PPP2R2B
Q00013	Q00013	DXS552E;EMP55;MPP1
Q00341	Q00341	HBP;HDLBP;VGL
Q00403	Q00403	GTF2B;TF2B;TFIIB
Q00535	Q00535	CDK5
Q00577	Q00577	PUR1;PURA
Q00765	Q00765	C5orf18;DP1;REEP5;TB2
Q00839	Q00839	HNRNPU;HNRPU;SAFA;U21.1

Q01082	Q01082	SPTB2;SPTBN1
Q01085	Q01085	TIAL1
Q01469	Q01469	FABP5
Q01813	Q01813	PFKF;PFKP
Q01968	Q01968	INPP5F;OCRL;OCRL1
Q02388	Q02388	COL7A1
Q02539	Q02539	H1F1;HIST1H1A
Q02543	Q02543	RPL18A
Q02750	Q02750	MAP2K1;MEK1;PRKMK1
Q02790	Q02790	FKBP4;FKBP52
Q02818	Q02818	NUC;NUCB1
Q02878	Q02878	RPL6;TXREB1
Q02880	Q02880	TOP2B
Q02952	Q02952	AKAP12;AKAP250
Q02978	Q02978	SLC20A4;SLC25A11
Q03169	Q03169	TNFAIP2
Q03518	Q03518	ABCB2;PSF1;RING4;TAP1;Y3
Q04323	Q04323	SAKS1;UBXN1
Q04917	Q04917	YWHA1;YWHAH
Q04941	Q04941	A4;PLP2
Q05315	Q05315	CLC
Q05707	Q05707	COL14A1;UND
Q06187	Q06187	AGMX1;ATK;BPK;BTK
Q06203	Q06203	GPAT;PPAT
Q06323	Q06323	IFI5111;PSME1
Q06495	Q06495	NPT2;SLC17A2;SLC34A1
Q07002	Q07002	CDK18;PCTK3
Q07020	Q07020	RPL18
Q07075	Q07075	ENPEP
Q07954	Q07954	A2MR;APR;LRP1
Q07955	Q07955	ASF;OK/SW-cl.3;SF2;SF2P33;SFRS1
Q08211	Q08211	DDX9;DHX9;LKP;NDH2
Q08257	Q08257	CRYZ
Q08379	Q08379	GOLGA2
Q08380	Q08380	LGALS3BP;M2BP
Q08722	Q08722	CD47;MER6
Q08881	Q08881	EMT;ITK;LYK
Q08945	Q08945	FACT80;SSRP1
Q08AM6	Q08AM6	TAX1BP2;TRX;VAC14
Q08J23	Q08J23	NSUN2;SAKI;TRM4
Q09028	Q09028	RBAP48;RBBP4
Q09161	Q09161	CBP80;NCBP;NCBP1
Q09666	Q09666	AHNAK;PM227
Q0VGL1	Q0VGL1	C7orf59
Q10471	Q10471	GALNT2
Q10567	Q10567	ADTB1;AP1B1;BAM22;CLAPB2

Q10570	Q10570	CPSF1;CPSF160
Q10589	Q10589	BST2
Q12768	Q12768	KIAA0196
Q12769	Q12769	KIAA0197;NUP120;NUP160
Q12792	Q12792	PTK9;TWF1
Q12824	Q12824	BAF47;INI1;SMARCB1;SNF5L1
Q12846	Q12846	STX4;STX4A
Q12874	Q12874	SAP61;SF3A3
Q12904	Q12904	AIMP1;EMAP2;SCYE1
Q12905	Q12905	ILF2;NF45;PRO3063
Q12907	Q12907	C5orf8;LMAN2
Q12959	Q12959	DLG1
Q12965	Q12965	MYO1C;MYO1E
Q12996	Q12996	CSTF3
Q13045	Q13045	FLII;FLIL
Q13057	Q13057	COASY;PSEC0106
Q13077	Q13077	EBI6;TRAF1
Q13098	Q13098	COPS1;CSN1;GPS1
Q13107	Q13107	UNP;UNPH;USP4
Q13144	Q13144	EIF2B5;EIF2BE
Q13148	Q13148	TARDBP;TDP43
Q13151	Q13151	HNRNPA0;HNRPA0
Q13155	Q13155	AIMP2;JTV1;PRO0992
Q13185	Q13185	CBX3
Q13200	Q13200	PSMD2;TRAP2
Q13242	Q13242	SFRS9;SRP30C
Q13263	Q13263	KAP1;RNF96;TIF1B;TRIM28
Q13291	Q13291	SLAM;SLAMF1
Q13303	Q13303	KCNA2B;KCNAB2;KCNK2
Q13308	Q13308	CCK4;PTK7
Q13325	Q13325	IFIT5;RI58
Q13347	Q13347	EIF3I;EIF3S2;TRIP1
Q13395	Q13395	TARBP1;TRM3;TRP185
Q13409	Q13409	DNCI2;DNCIC2;DYNC1I2
Q13418	Q13418	ILK;ILK1;ILK2
Q13435	Q13435	SAP145;SF3B2
Q13451	Q13451	AIG6;FKBP5;FKBP51
Q13459	Q13459	MYO9B;MYR5
Q13464	Q13464	ROCK1
Q13501	Q13501	ORCA;OSIL;SQSTM1
Q13509	Q13509	TUBB3;TUBB4
Q13555	Q13555	CAMK2G;CAMKG
Q13561	Q13561	DCTN2;DCTN50
Q13564	Q13564	APPBP1;HPP1;NAE1
Q13576	Q13576	IQGAP2
Q13596	Q13596	SNX1

Q13601	Q13601	HRB2;KRR1
Q13618	Q13618	CUL3;KIAA0617
Q13619	Q13619	CUL4A
Q13630	Q13630	SDR4E1;TSTA3
Q13642	Q13642	FHL1;SLIM1
Q13733	Q13733	ATP1A4;ATP1AL2
Q13740	Q13740	ALCAM;MEMD
Q13765	Q13765	HSD48;NACA
Q13813	Q13813	SPTA2;SPTAN1
Q13838	Q13838	BAT1;UAP56
Q13885	Q13885	TUBB2;TUBB2A
Q14103	Q14103	AUF1;HNRNPD;HNRPD
Q14108	Q14108	CD36L2;LIMPII;SCARB2
Q14139	Q14139	KIAA0126;UBE4A
Q14141	Q14141	KIAA0128;SEP2;SEPT6
Q14149	Q14149	KIAA0136;MORC3;ZCWCC3
Q14152	Q14152	EIF3A;EIF3S10;KIAA0139
Q14166	Q14166	KIAA0153;TTLL12
Q14185	Q14185	DOCK1
Q14195	Q14195	CRMP4;DPYSL3;DRP3;ULIP;ULIP1
Q14203	Q14203	DCTN1
Q14204	Q14204	DHC1;DNCH1;DNCL;DNECL;DYHC
Q14232	Q14232	EIF2B1;EIF2BA
Q14240	Q14240	DDX2B;EIF4A2;EIF4F
Q14254	Q14254	ESA1;FLOT2;M17S1
Q14258	Q14258	EFP;RNF147;TRIM25;ZNF147
Q14289	Q14289	FAK2;PTK2B;PYK2;RAFTK
Q14376	Q14376	GALE
Q14498	Q14498	HCC1;RBM39;RNPC2
Q14558	Q14558	PRPSAP1
Q14566	Q14566	MCM6
Q14644	Q14644	RASA3
Q14677	Q14677	CLINT1;ENTH;EPN4;EPNR;KIAA0171
Q14683	Q14683	DXS423E;KIAA0178;SB1.8
Q14697	Q14697	G2AN;GANAB;KIAA0088
Q14699	Q14699	KIAA0084;MIG2;RFTN1
Q14746	Q14746	COG2;LDLC
Q14761	Q14761	LPAP;PTPRCAP
Q14764	Q14764	LRP;MVP
Q14914	Q14914	LTB4DH;PTGR1
Q14974	Q14974	KPNB1;NTF97
Q14980	Q14980	NUMA;NUMA1
Q14997	Q14997	KIAA0077;PSME4
Q15008	Q15008	KIAA0107;PFAAP4;PSMD6
Q15018	Q15018	ABRO1;FAM175B;KIAA0157
Q15019	Q15019	DIFF6;KIAA0158;NEDD5;SEPT2

Q15020	Q15020	KIAA0156;SART3;TIP110
Q15021	Q15021	CAPD2;CNAP1;KIAA0159;NCAPD2
Q15024	Q15024	EXOSC7;KIAA0116;RRP42
Q15027	Q15027	ACAP1;CENTB1;KIAA0050
Q15029	Q15029	EFTUD2;KIAA0031;SNRP116
Q15036	Q15036	KIAA0064;SNX17
Q15046	Q15046	KARS;KIAA0070
Q15052	Q15052	ARHGEF6;COOL2;KIAA0006;PIXA
Q15080	Q15080	NCF4;SH3PXD4
Q15084	Q15084	PDIA6;TXNDC7
Q15102	Q15102	PAFAH1B3;PAFAHG
Q15111	Q15111	PLCL1
Q15126	Q15126	PMKI;PMVK
Q15181	Q15181	IOPPP;PP;PPA1
Q15185	Q15185	P23;PTGES3;TEBP
Q15233	Q15233	NONO;NRB54
Q15283	Q15283	GAP1M;RASA2;RASGAP
Q15286	Q15286	RAB1C;RAB35;RAY
Q15363	Q15363	RNP24;TMED2
Q15366	Q15366	PCBP2
Q15382	Q15382	RHEB;RHEB2
Q15386	Q15386	KIAA0010;UBE3C
Q15393	Q15393	KIAA0017;SAP130;SF3B3
Q15404	Q15404	RSP1;RSU1
Q15418	Q15418	MAPKAPK1A;RPS6KA1;RSK1
Q15436	Q15436	SEC23A
Q15437	Q15437	SEC23B
Q15459	Q15459	SAP114;SF3A1
Q15477	Q15477	DDX13;SKI2W;SKIV2;SKIV2L;W
Q15582	Q15582	BIGH3;TGFBI
Q15628	Q15628	TRADD
Q15645	Q15645	TRIP13
Q15773	Q15773	MLF2
Q15785	Q15785	TOMM34;URCC3
Q15813	Q15813	TBCE
Q15833	Q15833	STXBP2;UNC18B
Q15836	Q15836	SYB3;VAMP3
Q16186	Q16186	ADRM1;GP110
Q16401	Q16401	KIAA0072;PSMD5
Q16527	Q16527	CSRP2;LMO5;SMLIM
Q16531	Q16531	DDB1;XAP1
Q16537	Q16537	PPP2R5E
Q16543	Q16543	CDC37;CDC37A
Q16555	Q16555	CRMP2;DPYSL2;ULIP2
Q16563	Q16563	SYPL;SYPL1
Q16566	Q16566	CAMK4

Q16576	Q16576	RBAP46;RBBP7
Q16629	Q16629	SFRS7
Q16637	Q16637	SMN;SMN1;SMN2;SMNC;SMNT
Q16643	Q16643	D0S117E;DBN1
Q16658	Q16658	FAN1;FSCN1;HSN;SNL
Q16666	Q16666	IFI16;IFNGIP1
Q16698	Q16698	DECR;DECR1
Q16706	Q16706	MAN2A1;MANA2
Q16851	Q16851	UGP1;UGP2
Q17RC7	Q17RC7	C14orf73
Q1KMD3	Q1KMD3	HNRNPUL2;HNRPUL2
Q29RF7	Q29RF7	KIAA0648;PDS5;PDS5A;PIG54
Q2M389	Q2M389	KIAA1033
Q2M3G4	Q2M3G4	APXL2;KIAA1960;SHROOM1
Q2NL82	Q2NL82	KIAA1401;TSR1
Q2TAY7	Q2TAY7	SMU1
Q30154	Q30154	HLA-DRB5
Q32MZ4	Q32MZ4	GCF2;LRRFIP1;TRIP
Q32P44	Q32P44	EML3
Q3ZCM7	Q3ZCM7	TUBB8
Q460N5	Q460N5	BAL2;KIAA1268;PARP14
Q49A26	Q49A26	GLYR1;HIBDL;NP60
Q4G0J3	Q4G0J3	HDCMA18P;LARP7
Q52LJ0	Q52LJ0	FAM98B
Q53EL6	Q53EL6	H731;PDCD4
Q53GL7	Q53GL7	PARP10
Q53GS9	Q53GS9	CGI-21;HSPC332;PRO2855;USP39
Q53H96	Q53H96	PYCRL
Q53HC9	Q53HC9	TSSC1
Q53QZ3	Q53QZ3	ARHGAP15;BM-024;BM-030;BM-046
Q562R1	Q562R1	ACTBL2
Q567U6	Q567U6	CCDC93
Q5BKZ1	Q5BKZ1	ZNF326
Q5EBM0	Q5EBM0	CMPK2
Q5JSH3	Q5JSH3	WDR44
Q5JSL3	Q5JSL3	DOCK11;ZIZ2
Q5JV73	Q5JV73	FRMPD3;KIAA1817
Q5K651	Q5K651	C7orf5;DRIF1;KIAA2004;OEF1;SAMD9
Q5KU26	Q5KU26	CLP1;COLEC12;NSR2;SCARA4;SRCL
Q5RKV6	Q5RKV6	EXOSC6;MTR3
Q5SSJ5	Q5SSJ5	HP1BP3
Q5SY16	Q5SY16	NOL9
Q5T447	Q5T447	HECTD3
Q5T4S7	Q5T4S7	KIAA0462;KIAA1307;RBAF600
Q5T6J7	Q5T6J7	C9orf103
Q5T6V5	Q5T6V5	C9orf64

Q5TA45	Q5TA45	CPSF3L;INTS11;RC68
Q5TEJ8	Q5TEJ8	C1orf38;ICB1;THEMIS2
Q5TZA2	Q5TZA2	CROCC;KIAA0445
Q5VIR6	Q5VIR6	PP13624;VPS53
Q5VTR2	Q5VTR2	BRE1A;RNF20
Q5VVH5	Q5VVH5	IRAK1BP1
Q5VW38	Q5VW38	GPR107;KIAA1624;LUSTR1
Q5VWZ2	Q5VWZ2	LYPLAL1
Q5VYK3	Q5VYK3	ECM29;KIAA0368
Q658Y4	Q658Y4	FAM91A1
Q66K74	Q66K74	BPY2IP1;C19orf5;MAP1S;MAP8;VCY2IP1
Q66LE6	Q66LE6	KIAA1541;PPP2R2D
Q6DD88	Q6DD88	ATL3
Q6F5E8	Q6F5E8	LRRC16C;RLTPR
Q6I9Y2	Q6I9Y2	NIF3L1BP1;THOC7
Q6IA17	Q6IA17	SIGIRR;UNQ301/PRO342
Q6IA86	Q6IA86	ELP2;STATIP1
Q6L8Q7	Q6L8Q7	PDE12
Q6N069	Q6N069	NAA16;NARG1L;NAT2
Q6NXE6	Q6NXE6	ARMC6
Q6NXR4	Q6NXR4	C8orf41
Q6P2E9	Q6P2E9	EDC4;HEDLS
Q6P2Q9	Q6P2Q9	PRPC8;PRPF8
Q6P3X3	Q6P3X3	TTC27
Q6P6C2	Q6P6C2	ABH5;ALKBH5;OFOXD1
Q6PD62	Q6PD62	CTR9;KIAA0155;SH2BP1
Q6PGP7	Q6PGP7	KIAA0372;TTC37
Q6PIZ9	Q6PIZ9	HSPC062;TCRIM;TRAT1
Q6PJ69	Q6PJ69	TRIM65
Q6PJG6	Q6PJG6	C7orf27
Q6UB35	Q6UB35	FTHFSDC1;MTHFD1L
Q6UWD8	Q6UWD8	C16orf54;UNQ9389/PRO34280
Q6UXN9	Q6UXN9	UNQ9342/PRO34047;WDR82;WDR82A
Q6VN20	Q6VN20	KIAA1464;RANBP10
Q6ZNJ1	Q6ZNJ1	KIAA0540;NBEAL2;UNQ253/PRO290
Q6ZS81	Q6ZS81	C10orf64;KIAA1607;WDFY4
Q70CQ1	Q70CQ1	USP49
Q70IA6	Q70IA6	HCCA2;MOB2
Q71UM5	Q71UM5	RPS27L
Q7KZF4	Q7KZF4	SND1;TDRD11
Q7L014	Q7L014	DDX46;KIAA0801
Q7L1Q6	Q7L1Q6	BZAP45;BZW1;KIAA0005
Q7L2E3	Q7L2E3	DDX30;DHX30;KIAA0890
Q7L2H7	Q7L2H7	EIF3M;GA17;HFLB5;PCID1;PNAS-125
Q7L576	Q7L576	CYFIP1;KIAA0068
Q7L5D6	Q7L5D6	C7orf20;CEE;CGI-20

Q7Z2T5	Q7Z2T5	C1orf25;MSTP070;TRM1L
Q7Z2W4	Q7Z2W4	PRO1677;ZC3HAV1;ZC3HDC2
Q7Z2Z2	Q7Z2Z2	EFTUD1;FAM42A
Q7Z392	Q7Z392	C4orf41
Q7Z3J2	Q7Z3J2	101F10.2;C16orf62
Q7Z3U7	Q7Z3U7	KIAA1040;MON2;SF21
Q7Z3V4	Q7Z3V4	UBE3B
Q7Z406	Q7Z406	FP17425;KIAA2034;MYH14
Q7Z408	Q7Z408	CSMD2;KIAA1884
Q7Z4L5	Q7Z4L5	KIAA1992;Nbla10696;TTC21B
Q7Z5R6	Q7Z5R6	APBB1IP;PREL1;RARP1;RIAM
Q7Z6J4	Q7Z6J4	FGD2;ZFYVE4
Q7Z7H5	Q7Z7H5	ERS25;TMED4
Q7Z7K6	Q7Z7K6	CENPV;PRR6
Q86SF2	Q86SF2	GALNT7
Q86TI0	Q86TI0	KIAA1108;TBC1D1
Q86U38	Q86U38	C14orf21;KIAA2021
Q86U86	Q86U86	BAF180;PB1;PBRM1
Q86UX7	Q86UX7	FERMT3;KIND3;MIG2B;URP2
Q86V21	Q86V21	AACS;ACSF1
Q86VB7	Q86VB7	CD163;M130
Q86VP6	Q86VP6	CAND1;KIAA0829;TIP120;TIP120A
Q86W42	Q86W42	PSEC0006;THOC6;WDR58
Q86W50	Q86W50	METT10D
Q86WJ1	Q86WJ1	ALC1;CHD1L
Q86WV1	Q86WV1	SCAP1;SKAP1;SKAP55
Q86X76	Q86X76	NIT1
Q86XI2	Q86XI2	LUZP5;NCAPG2
Q86Y56	Q86Y56	HEATR2
Q86Y82	Q86Y82	STX12
Q86YJ6	Q86YJ6	THNSL2
Q86YV0	Q86YV0	RASAL3
Q86YV9	Q86YV9	HPS6
Q8IUE6	Q8IUE6	HIST2H2AB
Q8IUI8	Q8IUI8	CREME9;CRLF3;CYTOR4;P48
Q8IUR0	Q8IUR0	TRAPPC5
Q8IUR7	Q8IUR7	ARMC8;S863-2
Q8IVG5	Q8IVG5	C7orf6;DRIF2;KIAA2005;SAMD9L;UEF
Q8IW45	Q8IW45	CARKD
Q8IWA5	Q8IWA5	CTL2;PSEC0210;SLC44A2
Q8IWB7	Q8IWB7	KIAA1435;WDF1;WDFY1;ZFYVE17
Q8IXH7	Q8IXH7	HSPC130;NELFD;TH1;TH1L
Q8IXQ6	Q8IXQ6	BAL;PARP9
Q8IY21	Q8IY21	DDX60
Q8IYI6	Q8IYI6	EXOC8
Q8IYJ3	Q8IYJ3	SB146;SLP1;SYTL1

Q8IZ83	Q8IZ83	ALDH16A1
Q8IZL8	Q8IZL8	HMX3;MNAR;PELP1
Q8IZP0	Q8IZP0	ABI1;SSH3BP1
Q8N0W3	Q8N0W3	FUK
Q8N122	Q8N122	KIAA1303;RAPTOR;RPTOR
Q8N163	Q8N163	DBC1;KIAA1967
Q8N1B4	Q8N1B4	SACM2L;VPS52
Q8N1F7	Q8N1F7	KIAA0095;NUP93
Q8N1G2	Q8N1G2	FTSJD2;KIAA0082
Q8N1G4	Q8N1G4	KIAA1185;LRRC47
Q8N1K5	Q8N1K5	C6orf190;C6orf207;THEMIS
Q8N201	Q8N201	INTS1;KIAA1440;UNQ1821/PRO3434
Q8N3C0	Q8N3C0	ASCC3;HELIC1
Q8N3P4	Q8N3P4	KIAA0804;VPS8
Q8N5D0	Q8N5D0	KIAA1037;WDTC1
Q8N684	Q8N684	CPSF7
Q8N6R0	Q8N6R0	CGI-01;KIAA0859;METTL13
Q8N7H5	Q8N7H5	PAF1;PD2
Q8N9N7	Q8N9N7	LRRC57
Q8NB90	Q8NB90	AFG2;SPAF;SPATA5
Q8NBF2	Q8NBF2	NHLRC2
Q8ND71	Q8ND71	GIMAP8;IAN9;IANT
Q8NDA8	Q8NDA8	HEATR7A;KIAA1833
Q8NE71	Q8NE71	ABC50;ABCF1
Q8NEB9	Q8NEB9	PIK3C3;VPS34
Q8NEZ5	Q8NEZ5	FBX22;FBXO22
Q8NHH9	Q8NHH9	ARL6IP2;ATL2
Q8NHV4	Q8NHV4	NEDD1
Q8NI27	Q8NI27	CXorf3;THOC2
Q8TAG9	Q8TAG9	EXOC6;SEC15A;SEC15L;SEC15L1
Q8TAQ2	Q8TAQ2	BAF170;SMARCC2
Q8TCG1	Q8TCG1	CIP2A;KIAA1524
Q8TD19	Q8TD19	KIAA1995;NEK8;NEK9;NERCC
Q8TD55	Q8TD55	PLEKHO2;PLEKHQ1;PP9099
Q8TDZ2	Q8TDZ2	MICAL;MICAL1;NICAL
Q8TEQ6	Q8TEQ6	GEMIN5
Q8TF42	Q8TF42	KIAA1959;STS1;UBASH3B
Q8WTW3	Q8WTW3	COG1;KIAA1381;LDLB
Q8WUH2	Q8WUH2	TGFBRAP1
Q8WUM4	Q8WUM4	AIP1;ALIX;KIAA1375;PDCD6IP
Q8WUW1	Q8WUW1	C3orf10;HSPC300;MDS027
Q8WVB6	Q8WVB6	C16orf41;CHTF18;CTF18
Q8WVM8	Q8WVM8	C14orf163;FKSG23;KIAA0917;SCFD1
Q8WVT3	Q8WVT3	CGI-87;TTC15
Q8WWH5	Q8WWH5	PUS4;TRUB1
Q8WWI5	Q8WWI5	CD92;CDW92;CTL1;SLC44A1

Q8WXH0	Q8WXH0	KIAA1011;NUA;SYNE2
Q8WXX0	Q8WXX0	DNAH7;KIAA0944
Q8WYJ6	Q8WYJ6	DIFF6;PNUTL3;SEPT1
Q92499	Q92499	DDX1
Q92522	Q92522	H1FX
Q92542	Q92542	KIAA0253;NCSTN;UNQ1874/PRO4317
Q92556	Q92556	ELMO1;KIAA0281
Q92597	Q92597	CAP43;DRG1;NDRG1;RTP
Q92598	Q92598	HSP105;HSP110;HSPH1;KIAA0201
Q92600	Q92600	RCD1;RQCD1
Q92608	Q92608	DOCK2;KIAA0209
Q92614	Q92614	KIAA0216;MYO18A;MYSPDZ
Q92616	Q92616	GCN1L1;KIAA0219
Q92619	Q92619	HMHA1;KIAA0223
Q92769	Q92769	HDAC2
Q92797	Q92797	SPK;SYMPK
Q92835	Q92835	INPP5D;SHIP;SHIP1
Q92839	Q92839	HAS;HAS1
Q92841	Q92841	DDX17
Q92878	Q92878	RAD50
Q92879	Q92879	BRUNOL2;CELF1;CUGBP
Q92882	Q92882	OSTF1
Q92888	Q92888	ARHGEF1
Q92896	Q92896	CFR1;ESL1;GLG1;MG160
Q92900	Q92900	KIAA0221;RENT1;UPF1
Q92905	Q92905	COPS5;CSN5;JAB1
Q92922	Q92922	BAF155;SMARCC1
Q92930	Q92930	RAB8B
Q92973	Q92973	KPNB2;MIP1;TNPO1;TRN
Q92974	Q92974	ARHGEF2;KIAA0651;LFP40
Q92979	Q92979	C2F;EMG1
Q92990	Q92990	FAP48;FAP68;GLMN;VMGLOM
Q93009	Q93009	HAUSP;USP7
Q93084	Q93084	ATP2A3
Q93100	Q93100	PHKB
Q95IE3	Q95IE3	HLA-DRB1
Q969E2	Q969E2	SCAMP4
Q969G3	Q969G3	BAF57;SMARCE1
Q969P0	Q969P0	CD81P3;EWI2;IGSF8;KCT4
Q969U7	Q969U7	HCCA3;PAC2;PSMG2;TNFSF5IP1
Q96A08	Q96A08	HIST1H2BA;TSH2B
Q96A65	Q96A65	EXOC4;KIAA1699;SEC8;SEC8L1
Q96A72	Q96A72	MAGOH2;MAGOHB
Q96AJ9	Q96AJ9	VTI1A
Q96AX1	Q96AX1	VPS33A
Q96AX2	Q96AX2	RAB37

Q96BM9	Q96BM9	ARL10B;ARL8A;GIE2
Q96CD0	Q96CD0	FBL8;FBXL8
Q96CW1	Q96CW1	AP2M1;CLAPM1;KIAA0109
Q96CW5	Q96CW5	GCP3;TUBGCP3
Q96CX2	Q96CX2	C13orf2;KCTD12;KIAA1778;PFET1
Q96D46	Q96D46	CGI-07;NMD3
Q96DH6	Q96DH6	MSI2
Q96DI7	Q96DI7	PRP8BP;SFP38;SNRNP40;WDR57
Q96EE3	Q96EE3	SEC13L;SEH1;SEH1L
Q96F07	Q96F07	CYFIP2;KIAA1168;PIR121
Q96FQ6	Q96FQ6	AAG13;S100A16;S100F
Q96FV9	Q96FV9	HPR1;THOC1
Q96FW1	Q96FW1	HSPC263;OTB1;OTU1;OTUB1
Q96FX7	Q96FX7	C14orf172;TRM61;TRMT61A
Q96FZ7	Q96FZ7	CHMP6;VPS20
Q96G46	Q96G46	DUS3L
Q96GM5	Q96GM5	BAF60A;SMARCD1
Q96H79	Q96H79	C7orf39;ZC3HAV1L
Q96HN2	Q96HN2	AHCYL2;KIAA0828
Q96I24	Q96I24	FBP3;FUBP3
Q96IJ6	Q96IJ6	GMPPA
Q96IU4	Q96IU4	ABHD14B;CIB
Q96IV0	Q96IV0	NGLY1;PNG1
Q96JB2	Q96JB2	COG3;SEC34
Q96JC1	Q96JC1	KIAA0770;VAM6;VPS39
Q96JH7	Q96JH7	KIAA1850;VCIP135;VCPIP1
Q96JI7	Q96JI7	KIAA1840;SPG11
Q96JJ3	Q96JJ3	CED12A;ELMO2;KIAA1834
Q96KP1	Q96KP1	EXOC2;SEC5;SEC5L1
Q96KP4	Q96KP4	CN2;CNDP2;CPGL;PEPA
Q96L92	Q96L92	KIAA0488;My014;SNX27
Q96LJ7	Q96LJ7	DHRS1
Q96MG7	Q96MG7	HCA4;MAGEG1;NDNL2
Q96MM6	Q96MM6	C20orf60;HSPA12B
Q96PE3	Q96PE3	INPP4A
Q96PK6	Q96PK6	RBM14;SIP
Q96PX9	Q96PX9	KIAA1909;PLEKHG4B
Q96Q05	Q96Q05	KIAA1882;NIBP;T1;TRAPPC9
Q96QK1	Q96QK1	MEM3;TCCCTA00141;VPS35
Q96QR8	Q96QR8	PURB
Q96QU8	Q96QU8	KIAA0370;RANBP20;XPO6
Q96RQ9	Q96RQ9	FIG1;IL4I1;UNQ636/PRO1265
Q96RT1	Q96RT1	ERBB2IP;ERBIN;KIAA1225;LAP2
Q96S19	Q96S19	C16orf13;JFP2
Q96S44	Q96S44	C20orf64;PRPK;TP53RK
Q96S55	Q96S55	WHIP;WRNIP1

Q96S59	Q96S59	RANBP9;RANBPM
Q96S97	Q96S97	MYADM;UNQ553/PRO1110
Q96S99	Q96S99	APPD;LAPF;PLEKHF1;ZFYVE15
Q96T60	Q96T60	PNKP
Q96T76	Q96T76	MMS19;MMS19L
Q96T88	Q96T88	ICBP90;NP95;RNF106;UHRF1
Q96TA1	Q96TA1	C9orf88;FAM129B
Q99426	Q99426	CG22;CKAP1;TBCB
Q99436	Q99436	PSMB7;Z
Q99447	Q99447	PCYT2
Q99459	Q99459	CDC5L;KIAA0432;PCDC5RP
Q99460	Q99460	PSMD1
Q99497	Q99497	PARK7
Q99536	Q99536	VAT1
Q99570	Q99570	PIK3R4
Q99613	Q99613	EIF3C;EIF3CL;EIF3S8
Q99615	Q99615	DNAJC7;TPR2;TTC2
Q99623	Q99623	BAP;PHB2;REA
Q99653	Q99653	CHP
Q99715	Q99715	COL12A1;COL12A1L
Q99719	Q99719	PNUTL1;SEPT5
Q99733	Q99733	NAP1L4;NAP2
Q99747	Q99747	NAPG;SNAPG
Q99798	Q99798	ACO2
Q99808	Q99808	ENT1;SLC29A1
Q99829	Q99829	CPN1;CPNE1
Q99832	Q99832	CCT7;CCTH;NIP7-1
Q99836	Q99836	MYD88
Q99879	Q99879	H2BFE;HIST1H2BM
Q99973	Q99973	TEP1;TLP1;TP1
Q99986	Q99986	VRK1
Q9BPX3	Q9BPX3	CAPG;NCAPG;NYMEL3
Q9BPX5	Q9BPX5	ARPC5L
Q9BQ39	Q9BQ39	DDX50
Q9BQ67	Q9BQ67	GRWD;GRWD1;KIAA1942;WDR28
Q9BQA1	Q9BQA1	HKMT1069;MEP50;Nbla10071;WDR77
Q9BQE3	Q9BQE3	TUBA1C;TUBA6
Q9BQE5	Q9BQE5	APOL2
Q9BQG0	Q9BQG0	MYBBP1A;P160
Q9BQK8	Q9BQK8	LIPN3L;LPIN3
Q9BRJ7	Q9BRJ7	NUDT16L1;SDOS
Q9BRR9	Q9BRR9	ARHGAP9
Q9BRX2	Q9BRX2	CGI-17;PELO
Q9BRX8	Q9BRX8	C10orf58;PRO2290;PSEC0139
Q9BRZ2	Q9BRZ2	RNF109;TRIM56
Q9BSD7	Q9BSD7	C1orf57

Q9BSJ2	Q9BSJ2	GCP2;TUBGCP2
Q9BSJ8	Q9BSJ8	ESYT1;FAM62A;KIAA0747;MBC2
Q9BT78	Q9BT78	COPS4;CSN4
Q9BTC8	Q9BTC8	KIAA1266;MTA3
Q9BTT0	Q9BTT0	ANP32E
Q9BTW9	Q9BTW9	KIAA0988;PP1096;SSD1;TBCD;TFCD
Q9BUF5	Q9BUF5	TUBB6
Q9BUI4	Q9BUI4	POLR3C
Q9BUJ2	Q9BUJ2	E1BAP5;HNRNPUL1;HNRPUL1
Q9BUL8	Q9BUL8	CCM3;PDCD10;TFAR15
Q9BUP3	Q9BUP3	CC3;HTATIP2;TIP30
Q9BUT1	Q9BUT1	BDH2;DHRS6;UNQ6308/PRO20933
Q9BV38	Q9BV38	WDR18
Q9BV40	Q9BV40	VAMP8
Q9BV86	Q9BV86	AD-003;C9orf32;METTL11A
Q9BVA1	Q9BVA1	TUBB2B
Q9BVC4	Q9BVC4	GBL;LST8;MLST8
Q9BVC6	Q9BVC6	TMEM109
Q9BVK6	Q9BVK6	GP25L2;TMED9
Q9BVQ7	Q9BVQ7	SPATA5L1
Q9BW19	Q9BW19	HSET;KIFC1;KNSL2
Q9BXJ9	Q9BXJ9	GA19;NAA15;NARG1;NATH;TBDN100
Q9BXN1	Q9BXN1	ASPN;PLAP1;SLRR1C;UNQ215/PRO241
Q9BXP5	Q9BXP5	ARS2;ASR2;SRRT
Q9BXR0	Q9BXR0	QTRT1;TGT;TGUT
Q9BXS5	Q9BXS5	AP1M1;CLTNM
Q9BY32	Q9BY32	C20orf37;ITPA;My049;OK/SW-cl.9
Q9BYB4	Q9BYB4	FKSG1;GNB1L;GY2;KIAA1645;WDR14
Q9BZ23	Q9BZ23	C20orf48;PANK2
Q9BZG1	Q9BZG1	RAB34;RAB39;RAH
Q9BZH6	Q9BZH6	BRWD2;KIAA1351;WDR11;WDR15
Q9BZJ0	Q9BZJ0	CGI-201;CRN;CRNKL1;MSTP021
Q9BZK7	Q9BZK7	IRA1;TBL1XR1;TBLR1
Q9BZL6	Q9BZL6	HSPC187;PKD2;PRKD2
Q9BZQ8	Q9BZQ8	C1orf24;FAM129A;GIG39;NIBAN
Q9BZZ2	Q9BZZ2	SIGLEC1;SN
Q9C0E2	Q9C0E2	KIAA1721;XPO4
Q9GZN7	Q9GZN7	ROGDI
Q9GZP4	Q9GZP4	AD039;C1orf128;HT014;PP603
Q9GZS3	Q9GZS3	WDR61
Q9GZZ1	Q9GZZ1	MAK3;NAA50;NAT13;NAT5
Q9H0A0	Q9H0A0	ALP;KIAA1709;NAT10
Q9H0C8	Q9H0C8	ILKAP
Q9H0J9	Q9H0J9	PARP12;ZC3HDC1
Q9H0N0	Q9H0N0	RAB6C;WTH3
Q9H0Q0	Q9H0Q0	FAM49A

Q9H0W9	Q9H0W9	C11orf54;LP4947;PTD012
Q9H223	Q9H223	EHD4;FKSG7;HCA10;HCA11;PAST4
Q9H267	Q9H267	VPS33B
Q9H269	Q9H269	VPS16
Q9H270	Q9H270	PP3476;RNF108;VPS11
Q9H299	Q9H299	P1725;SH3BGRL3
Q9H2K8	Q9H2K8	DPK;JIK;KDS;MAP3K18;TAOK3
Q9H2U2	Q9H2U2	HSPC124;PPA2
Q9H3G5	Q9H3G5	CPVL;PSEC0124;UNQ197/PRO223;VLP
Q9H3K6	Q9H3K6	BOLA2;BOLA2A;BOLA2B;My016
Q9H3N1	Q9H3N1	PSEC0085;TMX;TMX1;TXNDC
Q9H3P7	Q9H3P7	ACBD3;GCP60;GOCAP1;GOLPH1
Q9H3U1	Q9H3U1	SMAP1;UNC45A
Q9H400	Q9H400	LIME;LIME1;LP8067
Q9H444	Q9H444	C20orf178;CHMP4B;SHAX1
Q9H4A6	Q9H4A6	GOLPH3;GPP34
Q9H4G4	Q9H4G4	C9orf19;GAPR1;GLIPR2
Q9H4M9	Q9H4M9	CDABP0131;EHD1;PAST;PAST1
Q9H4Z3	Q9H4Z3	C20orf67;PCIF1
Q9H7D0	Q9H7D0	DOCK5
Q9H7D7	Q9H7D7	CDW2;MIP2;PRO0852;WDR26
Q9H8W4	Q9H8W4	PLEKHF2;ZFYVE18
Q9H8Y8	Q9H8Y8	GOLPH6;GORASP2
Q9H944	Q9H944	MED20;TRFP
Q9H9E3	Q9H9E3	COG4
Q9H9F9	Q9H9F9	ACTR5;ARP5
Q9H9H4	Q9H9H4	VPS37B
Q9H9T3	Q9H9T3	ELP3
Q9HA64	Q9HA64	FN3KRP
Q9HAS0	Q9HAS0	C17orf75
Q9HAV0	Q9HAV0	GNB4
Q9HAV4	Q9HAV4	KIAA1291;RANBP21;XPO5
Q9HBH5	Q9HBH5	PAN2;RDH14;UNQ529/PRO1072
Q9HBI0	Q9HBI0	PARVG
Q9HBL8	Q9HBL8	HSCARG;NMRAL1
Q9HC35	Q9HC35	C2orf2;EMAPL4;EML4
Q9HCE1	Q9HCE1	KIAA1631;MOV10
Q9HCF4	Q9HCF4	ALO17;KIAA1618
Q9HCS7	Q9HCS7	HCNP;KIAA1177;PP3898;SYF1;XAB2
Q9HCY8	Q9HCY8	S100A14;S100A15
Q9NP72	Q9NP72	RAB18
Q9NP79	Q9NP79	C6orf55;HSPC228;My012;VTA1
Q9NPD3	Q9NPD3	EXOSC4;RRP41;SKI6
Q9NPE3	Q9NPE3	NOLA3;NOP10
Q9NPF4	Q9NPF4	GCPL1;OSGEP
Q9NPF5	Q9NPF5	DMAP1;KIAA1425

Q9NQC3	Q9NQC3	KIAA0886;My043;NOGO;RTN4;SP1507
Q9NQE7	Q9NQE7	PRSS16;TSSP
Q9NQI0	Q9NQI0	DDX4;VASA
Q9NQR4	Q9NQR4	CUA002;NIT2
Q9NQT4	Q9NQT4	CML28;EXOSC5;RRP46
Q9NQT5	Q9NQT5	CGI-102;EXOSC3;RRP40
Q9NQW7	Q9NQW7	XPNPEP1;XPNPEPL;XPNPEPL1
Q9NQZ6	Q9NQZ6	HCA127;KIAA1166;ZC4H2
Q9NR30	Q9NR30	DDX21
Q9NR45	Q9NR45	NANS;SAS
Q9NR48	Q9NR48	ASH1L;KIAA1420;KMT2H
Q9NR50	Q9NR50	EIF2B3
Q9NR56	Q9NR56	EXP;KIAA0428;MBNL;MBNL1
Q9NRD1	Q9NRD1	FBG2;FBS2;FBX6;FBXO6
Q9NRF8	Q9NRF8	CTPS2
Q9NRW3	Q9NRW3	APOBEC1L;APOBEC3C;PBI
Q9NRW7	Q9NRW7	VPS45;VPS45A;VPS45B
Q9NRZ9	Q9NRZ9	HELLS;Nbla10143;PASG;SMARCA6
Q9NS87	Q9NS87	KIF15;KLP2;KNSL7
Q9NSD9	Q9NSD9	FARSB;FARSLB;FRSB;HSPC173
Q9NSK0	Q9NSK0	KLC4;KNSL8
Q9NT62	Q9NT62	APG3;APG3L;ATG3
Q9NTI5	Q9NTI5	APRIN;AS3;KIAA0979;PDS5B
Q9NTJ3	Q9NTJ3	CAPC;SMC4;SMC4L1
Q9NU22	Q9NU22	KIAA0301;MDN1
Q9NUQ8	Q9NUQ8	ABCF3
Q9NUQ9	Q9NUQ9	BM-009;FAM49B
Q9NUV9	Q9NUV9	GIMAP4;IAN1;IMAP4;MSTP062
Q9NV31	Q9NV31	C15orf12;IMP3;MRPS4
Q9NV70	Q9NV70	BM-012;EXOC1;SEC3;SEC3L1
Q9NVA2	Q9NVA2	11-sep
Q9NVE7	Q9NVE7	PANK4
Q9NVI1	Q9NVI1	FANCI;KIAA1794
Q9NVJ2	Q9NVJ2	ARL10C;ARL8B;GIE1
Q9NW08	Q9NW08	POLR3B
Q9NW64	Q9NW64	199G4;RBM22;ZC3H16
Q9NW82	Q9NW82	WDR70
Q9NWU2	Q9NWU2	C20orf11
Q9NWV8	Q9NWV8	C19orf62;HSPC142;MERIT40;NBA1
Q9NXF1	Q9NXF1	L18;Nbla10363;TEX10
Q9NXK8	Q9NXK8	FBL12;FBXL12
Q9NXR7	Q9NXR7	BRCC45;BRE
Q9NYT0	Q9NYT0	PLEK2
Q9NZ08	Q9NZ08	APPILS;ARTS1;ERAP1;KIAA0525
Q9NZB2	Q9NZB2	C9orf10;FAM120A;KIAA0183;OSSA
Q9NZD8	Q9NZD8	ACP33;BM-019;GL010;SPG21

Q9NZJ9	Q9NZJ9	DIPP2;HDCMB47P;KIAA0487;NUDT4
Q9NZL9	Q9NZL9	MAT2B;MSTP045;Nbla02999;TGR
Q9NZN4	Q9NZN4	EHD2;PAST2
Q9NZT1	Q9NZT1	CALML5;CLSP
Q9P016	Q9P016	HSPC144;MDS012;My0054;THY28;THYN1
Q9P0L0	Q9P0L0	VAP33;VAPA
Q9P0M6	Q9P0M6	H2AFY2;MACROH2A2
Q9P0V9	Q9P0V9	10-sep
Q9P241	Q9P241	ATP10D;ATPVD;KIAA1487
Q9P253	Q9P253	KIAA1475;VPS18
Q9P258	Q9P258	KIAA1470;RCC2;TD60
Q9P265	Q9P265	DIP2B;KIAA1463
Q9P2A4	Q9P2A4	ABI3;NESH
Q9P2B2	Q9P2B2	CD9P1;EWIF;FPRP;KIAA1436;PTGFRN
Q9P2J5	Q9P2J5	KIAA1352;LARS
Q9P2P6	Q9P2P6	KIAA1300;STARD9
Q9P2R3	Q9P2R3	ANKFY1;ANKHZN;KIAA1255
Q9TQE0	Q9TQE0	HLA-DRB1
Q9UBB4	Q9UBB4	ATXN10;SCA10
Q9UBF2	Q9UBF2	COPG2
Q9UBG3	Q9UBG3	C1orf10;CRNN;DRC1;PDRC1;SEP53
Q9UBI1	Q9UBI1	BUP;C10orf8;COMMD3
Q9UBI6	Q9UBI6	GNG12
Q9UBN7	Q9UBN7	HDAC6;JM21;KIAA0901
Q9UBQ0	Q9UBQ0	DC15;DC7;MDS007;VPS29
Q9UBQ5	Q9UBQ5	ARG134;EIF3K;EIF3S12;HSPC029;MSTP001
Q9UBQ7	Q9UBQ7	GLXR;GRHPR;MSTP035
Q9UBT2	Q9UBT2	HRIHFB2115;SAE2;UBA2;UBLE1B
Q9UBU9	Q9UBU9	NXF1;TAP
Q9UBV8	Q9UBV8	ABP32;PEF1;UNQ1845/PRO3573
Q9UDY8	Q9UDY8	MALT1;MLT
Q9UEU0	Q9UEU0	VTI1;VTI1B;VTI1L;VTI1L1;VTI2
Q9UEW8	Q9UEW8	SPAK;STK39
Q9UG63	Q9UG63	ABCF2;HUSSY-18
Q9UGJ1	Q9UGJ1	76P;GCP4;TUBGCP4
Q9UH65	Q9UH65	HSPC321;KIAA0640;SWAP70
Q9UHB9	Q9UHB9	SRP68
Q9UHD2	Q9UHD2	NAK;TBK1
Q9UHD8	Q9UHD8	KIAA0991;MSF;SEPT9
Q9UHD9	Q9UHD9	HRIHFB2157;N4BP4;PLIC2;UBQLN2
Q9UHQ9	Q9UHQ9	CYB5R1;NQO3A2;UNQ3049/PRO9865
Q9UI08	Q9UI08	EVL;RNB6
Q9UI10	Q9UI10	EIF2B4;EIF2BD
Q9UI12	Q9UI12	ATP6V1H;CGI-11
Q9UI30	Q9UI30	AD-001;HSPC152;HSPC170;TRMT112
Q9UIA9	Q9UIA9	KIAA0745;RANBP16;XPO7

Q9UIC8	Q9UIC8	CGI-68;LCMT;LCMT1
Q9UID3	Q9UID3	ANG2;C11orf2;C11orf3;FFR;PP5382
Q9UIQ6	Q9UIQ6	LNPEP;OTASE
Q9UIV1	Q9UIV1	CAF1;CNOT7
Q9UJ70	Q9UJ70	NAGK
Q9UJU6	Q9UJU6	CMAP;DBNL;PP5423;SH3P7
Q9UJX2	Q9UJX2	ANAPC8;CDC23
Q9UJX3	Q9UJX3	ANAPC7;APC7
Q9UK22	Q9UK22	FBX2;FBXO2
Q9UK41	Q9UK41	VPS28
Q9UKF6	Q9UKF6	CPSF3;CPSF73
Q9UKK3	Q9UKK3	ADPRTL1;KIAA0177;PARP4;PARPL
Q9UKZ1	Q9UKZ1	C2orf29;C40
Q9UL25	Q9UL25	KIAA0118;RAB21
Q9UL46	Q9UL46	PSME2
Q9ULA0	Q9ULA0	ASPEP;DAP;DNPEP
Q9ULC3	Q9ULC3	HSPC137;RAB23
Q9ULK4	Q9ULK4	ARC130;CRSP3;DRIP130;KIAA1216;MED23
Q9ULP9	Q9ULP9	KIAA1171;TBC1D24
Q9ULR0	Q9ULR0	ISY1;KIAA1160
Q9ULT0	Q9ULT0	KIAA1140;TTC7;TTC7A
Q9UM54	Q9UM54	KIAA0389;MYO6
Q9UMR2	Q9UMR2	DBP5;DDX19;DDX19B
Q9UMS4	Q9UMS4	NMP200;PRP19;PRPF19;SNEV
Q9UMZ2	Q9UMZ2	AP1GBP1;SYNG;SYNRG
Q9UNA1	Q9UNA1	ARHGAP26;GRAF;KIAA0621;OPHN1L
Q9UNE7	Q9UNE7	CHIP;PP1131;STUB1
Q9UNH7	Q9UNH7	SNX6
Q9UNM6	Q9UNM6	PSMD13
Q9UNP9	Q9UNP9	CYP33;PPIE
Q9UNQ2	Q9UNQ2	DIMT1;DIMT1L;HUSSY-05
Q9UP83	Q9UP83	COG5;GOLTC1;GTC90
Q9UPN7	Q9UPN7	KIAA1115;PP6R1;SAPS1
Q9UPT5	Q9UPT5	EXO70;EXOC7;KIAA1067
Q9UPU5	Q9UPU5	KIAA1057;USP24
Q9UQ80	Q9UQ80	EBP1;PA2G4
Q9UQE7	Q9UQE7	BAM;BMH;CSPG6;SMC3;SMC3L1
Q9Y223	Q9Y223	GLCNE;GNE
Q9Y224	Q9Y224	C14orf166;CGI-99
Q9Y230	Q9Y230	CGI-46;INO80J;RUVBL2;TIP48;TIP49B
Q9Y262	Q9Y262	EIF3EIP;EIF3L;EIF3S6IP;HSPC021;HSPC025
Q9Y263	Q9Y263	PLAA;PLAP
Q9Y265	Q9Y265	INO80H;NMP238;RUVBL1;TIP49;TIP49A
Q9Y281	Q9Y281	CFL2
Q9Y296	Q9Y296	CGI-104;HSPC172;PTD009;SBDN;TRAPPC4
Q9Y2A7	Q9Y2A7	HEM2;KIAA0587;NAP1;NCKAP1

Q9Y2H1	Q9Y2H1	KIAA0965;NDR2;STK38L
Q9Y2I8	Q9Y2I8	KIAA0982;WDR37
Q9Y2J2	Q9Y2J2	DAL1;EPB41L3;KIAA0987
Q9Y2J8	Q9Y2J8	KIAA0994;PADI2;PDI2
Q9Y2L1	Q9Y2L1	DIS3;KIAA1008;RRP44
Q9Y2L5	Q9Y2L5	KIAA1012
Q9Y2L8	Q9Y2L8	KIAA1015;ZFP95;ZKSCAN5
Q9Y2P8	Q9Y2P8	HSPC338;RCL1;RNAC;RPC2;RPCL1;RTC2
Q9Y2V2	Q9Y2V2	CARHSP1
Q9Y2V7	Q9Y2V7	COG6;KIAA1134
Q9Y2Z0	Q9Y2Z0	SUGT1
Q9Y315	Q9Y315	CGI-26;DERA
Q9Y316	Q9Y316	C2orf4;CGI-27;MEMO1;NS5ATP7
Q9Y333	Q9Y333	C6orf28;G7B;LSM2
Q9Y3B3	Q9Y3B3	CGI-109;TMED7
Q9Y3B4	Q9Y3B4	CGI-110;HSPC175;HT006;SF3B14
Q9Y3F4	Q9Y3F4	MAWD;STRAP;UNRIP
Q9Y3I0	Q9Y3I0	C22orf28;HSPC117
Q9Y3P8	Q9Y3P8	SIT;SIT1
Q9Y3U8	Q9Y3U8	RPL36
Q9Y3Z3	Q9Y3Z3	MOP5;SAMHD1
Q9Y450	Q9Y450	HBS1;HBS1L;KIAA1038
Q9Y4A5	Q9Y4A5	PAF400;TRRAP
Q9Y4C2	Q9Y4C2	FAM115A;KIAA0738
Q9Y4E6	Q9Y4E6	KIAA0541;TRAG;WDR7
Q9Y4E8	Q9Y4E8	KIAA0529;USP15
Q9Y4L1	Q9Y4L1	GRP170;HYOU1;ORP150
Q9Y4R8	Q9Y4R8	KIAA0683;TELO2
Q9Y4W2	Q9Y4W2	LAS1L;MSTP060
Q9Y530	Q9Y530	C6orf130
Q9Y5A7	Q9Y5A7	NUB1;NYREN18
Q9Y5B9	Q9Y5B9	FACT140;FACTP140;SUPT16H
Q9Y5K5	Q9Y5K5	AD-019;CGI-70;UCH37;UCHL5
Q9Y5P6	Q9Y5P6	GMPPB
Q9Y5Q8	Q9Y5Q8	CDABP0017;GTF3C5
Q9Y5X3	Q9Y5X3	SNX5
Q9Y5Y2	Q9Y5Y2	NUBP2
Q9Y5Z4	Q9Y5Z4	C6orf34;HEBP2;SOUL
Q9Y624	Q9Y624	F11R;JAM1;JCAM;UNQ264/PRO301
Q9Y678	Q9Y678	COPG;COPG1
Q9Y6G9	Q9Y6G9	DNCLI1;DYNC1LI1
Q9Y6K5	Q9Y6K5	OAS3;P/OKcl.4
Q9Y6K9	Q9Y6K9	FIP3;IKBKG;NEMO
Q9Y6V7	Q9Y6V7	DDX49
Q9Y6W5	Q9Y6W5	WASF2;WAVE2
A0A5B9	A0A5B9;P01850	TCRBC2;TRBC2;TRBC1

A6NIH7	A6NIH7;Q13432	UNC119B
A8MWD9	A8MWD9;P62308	PBSCG;SNRPG
B9A064	B9A064;P0CG04	IGLC1
O00203	O00203;Q13367	ADTB3A;AP3B1
O00410	O00410;O60518	IPO5;KPNB3;RANBP5
O00571	O00571;O15523	DBX;DDX3;DDX3X;DBY;DDX3Y
O14818	O14818;Q8TAA3	HSPC;PSMA7
O15066	O15066;Q9Y496	KIAA0359;KIF3B;KIF3;KIF3A
O43143	O43143;Q14562	DBP1;DDX15;DHX15
O43488	O43488;Q8NHP1	AFAR;AFAR1;AKR7;AKR7A2
O43795	O43795;Q9UBC5	MYO1B
O60234	O60234;P60983	GMFG
O60264	O60264;P28370	SMARCA5;SNF2H;WCRF135
O60488	O60488;O95573	ACS4;ACSL4;FACL4;LACS4
O60684	O60684;O15131	IPOA7;KPNA6;KPNA5
O60825	O60825;P16118	PFKFB2;F6PK;PFKFB1;PFRX
O75348	O75348;O95670	ATP6G;ATP6G1;ATP6J;ATP6V1G1;ATP6G2
O94776	O94776;Q13330	MTA1L1;MTA2;PID
O95197	O95197;Q16799	ASYIP;NSPL2;RTN3;NSP;RTN1
O95361	O95361;Q309B1	EBBP;TRIM16
O95758	O95758;Q9UKA9	ROD1
O96019	O96019;O94805	ACTL6A;BAF53;BAF53A;INO80K
P00374	P00374;Q86XF0	DHFR;DHFRP1
P00738	P00738;P00739	HP
P01111	P01111;P01112	HRAS1;NRAS;HRAS
P01766	P01766;P01777	
P01903	P01903;P01906	HLA-DRA;HLA-DRA1
P01912	P01912;Q5Y7A7	HLA-DRB1
P02649	P02649	APOE
P02675	P02675	FGB
P05388	P05388;Q8NHW5	RPLP0
P05556	P05556;P18564	FNRB;ITGB1;MDF2;MSK12
P06241	P06241;P12931	FYN;SRC;SRC1
P06493	P06493;Q00526	CDC2
P07355	P07355;A6NMY6	ANX2;ANX2L4;ANXA2;CAL1H;LPC2D;ANX2L2
P07737	P07737	PFN1
P07910	P07910;O60812	HNRNPC;HNRPC
P08107	P08107;P48741	HSPA1;HSPA1A;HSPA1B
P08670	P08670;P17661	VIM
P09651	P09651;Q32P51	HNRNPA1;HNRPA1;HNRNPA1L;HNRNPA1L2
P09914	P09914;Q5T764	G10P1;IFI56;IFIT1;IFNAI1
P0C0L4	P0C0L4;P0C0L5	C4A;CO4;CPAMD2;C4B;CPAMD3
P0C0S5	P0C0S5;Q71UI9	H2AFZ;H2AZ;H2AFV;H2AV
P10314	P10314;P30459	HLAA;HLA-A
P10398	P10398;P15056	ARAF;ARAF1;PKS;PKS2
P10412	P10412;P22492	H1F4;HIST1H1E

P10644	P10644;P31321	PKR1;PRKAR1;PRKAR1A;TSE1
P10966	P10966;A6NJW9	CD8B;CD8B1;CD8B2;CD8BP
P11216	P11216;P06737	PYGB
P11387	P11387;Q969P6	TOP1
P13807	P13807;P54840	GYS;GYS1
P13861	P13861;P31323	PKR2;PRKAR2;PRKAR2A
P14618	P14618;P30613	OIP3;PK2;PK3;PKM;PKM2
P14625	P14625;Q58FF3	GRP94;HSP90B1;TRA1
P14678	P14678;P63162	COD;SNRPB;SNRPB1;HCERN3;SMN;SNRPN
P15121	P15121;C9JRZ8	AKR1B1;ALDR1;AKR1B15
P15153	P15153;P60763	RAC2;RAC3
Q8IYD1	Q8IYD1;P15170	ERF3B;GSPT2;ERF3A;GSPT1
P15498	P15498;Q9UKW4	VAV;VAV1
P16104	P16104;Q96QV6	H2AFX;H2AX;H2AFR;HIST1H2AA
P16949	P16949;Q93045	LAP18;OP18;STMN1
P20340	P20340;Q9NRW1	RAB6;RAB6A;RAB6B
P20702	P20702;Q13349	CD11C;ITGAX
P21127	P21127;Q9UQ88	CDC2L1;CDK11;CDK11B;PITSLREA;PK58
P21281	P21281;P15313	ATP6B2;ATP6V1B2;VPP3
P21333	P21333;Q14315	FLN;FLN1;FLNA
P21399	P21399;O00408	ACO1;IREB1
P22087	P22087;A6NHQ2	FBL;FIB1;FLRN
P23258	P23258;Q9NRH3	TUBG;TUBG1;TUBG2
P23458	P23458;P29597	JAK1;JAK1A;JAK1B
P23743	P23743;Q9Y6T7	DAGK;DAGK1;DGKA
P25098	P25098;P35626	ADRBK1;BARK;BARK1;GRK2
P27635	P27635;Q96L21	DXS648E;QM;RPL10;RPL10L
P30419	P30419;O60551	NMT;NMT1
P31146	P31146	CORO1;CORO1A
P36543	P36543;Q96A05	ATP6E;ATP6E2;ATP6V1E1
P37802	P37802;Q9UI15	CDABP0035;KIAA0120;TAGLN2
P40227	P40227;Q92526	CCT6;CCT6A;CCTZ
P40429	P40429;Q6NVV1	RPL13A
P41091	P41091;Q2VIR3	EIF2G;EIF2S3;EIF2S3L
P41227	P41227;Q9BSU3	ARD1;ARD1A;NAA10;TE2
P42229	P42229;P51692	STAT5;STAT5A;STAT5B
P46783	P46783;Q9NQ39	RPS10
P46940	P46940;Q86VI3	IQGAP1;KIAA0051
P50148	P50148;O95837	GAQ;GNAQ
P50225	P50225;P50226	OK/SW-cl.88;STP;STP1;SULT1A1
P50749	P50749;Q9H2L5	KIAA0168;RASSF2;AD037;RASSF4
P50995	P50995;P27216	ANX11;ANXA11
P51151	P51151;Q9NP90	RAB9;RAB9A
P51178	P51178;Q4KWH8	PLCD1
P51532	P51532;P51531	BAF190A;BRG1;SMARCA4;SNF2B;SNF2L4
P51812	P51812;Q9UK32	ISPK1;MAPKAPK1B;RPS6KA3;RSK2

P54619	P54619;Q9UGJ0	PRKAG1
P54920	P54920;Q9H115	NAPA;SNAPA
P55036	P55036;A2A3N6	MCB1;PSMD4
P55072	P55072;Q8IYT4	VCP
P55786	P55786;A6NEC2	NPEPPS;PSA;NPEPPSL1
P58876	P58876;Q99880	H2BFB;HIRIP2;HIST1H2BD;H2BFC;HIST1H2BL
P60660	P60660;P14649	MYL6
P60891	P60891;P21108	PRPS1;PRPS1L1;PRPS3;PRPSL
P61018	P61018;P20338	PP1596;RAB4B;RAB4;RAB4A
P61019	P61019;Q8WUD1	RAB2;RAB2A;RAB2B
P61158	P61158;Q9P1U1	ACTR3;ARP3
P61204	P61204;P84077	ARF3;ARF1
P61224	P61224;A6NIZ1	OK/SW-cl.11;RAP1B
P62304	P62304;Q5VYJ4	SNRPE;SNRPEL1
P62330	P62330;P36406	ARF6
P62854	P62854;Q5JNZ5	RPS26;RPS26L1;RPS26P11
P62873	P62873;P16520	GNB1
P63151	P63151;Q9Y2T4	PPP2R2A
P63167	P63167;Q96FJ2	DLC1;DNCL1;DNCLC1;DYNLL1;HDLC1;DLC2
P68371	P68371;Q99867	TUBB2C
Q00610	Q00610;P53675	CLH17;CLTC;CLTCL2;KIAA0034
Q01081	Q01081;Q8WU68	U2AF1;U2AF35;U2AFBP
Q01518	Q01518;P40123	CAP;CAP1
Q01826	Q01826;Q9UPW6	SATB1
Q06210	Q06210;O94808	GFAT;GFPT;GFPT1
Q06830	Q06830;Q13162	PAGA;PAGB;PRDX1;TDPX2
Q07866	Q07866;Q9H0B6	KLC;KLC1;KNS2
Q08AF3	Q08AF3;P0C7X5	SLFN5
Q12906	Q12906;Q96SI9	DRBF;ILF3;MPHOSPH4;NF90
Q13043	Q13043;Q13188	MST1;STK4
Q13085	Q13085;O00763	ACAC;ACACA;ACC1;ACCA
Q13131	Q13131;P54646	AMPK1;PRKAA1
Q13363	Q13363;P56545	CTBP;CTBP1
Q13492	Q13492;O60641	CALM;PICALM
Q13547	Q13547;O15379	HDAC1;RPD3L1
Q13637	Q13637;P57729	RAB32
Q14160	Q14160;Q9BTT6	CRIB1;KIAA0147;LAP4;SCRB1;SCRIB;VARTUL
Q15149	Q15149;P58107	PLEC1
Q15907	Q15907;P62491	RAB11B;YPT3;RAB11;RAB11A
Q3KQV9	Q3KQV9;Q16222	UAP1L1;SPAG2;UAP1
Q5VZM2	Q5VZM2;Q7L523	RRAGB;RRAGA
Q6FI13	Q6FI13;Q16777	H2AFO;HIST2H2AA;HIST2H2AA3;HIST2H2AA4
Q6WKZ4	Q6WKZ4;Q7L804	RAB11FIP1;RCP;KIAA0941;RAB11FIP2
Q7L9L4	Q7L9L4;Q9H8S9	MOB4A;MOBKL1A;C2orf6;MOB4B;MOBK1B
Q7Z7L1	Q7Z7L1;Q68D06	SLFN11
Q8N8A2	Q8N8A2;O15084	ANKRD44

Q8NF50	Q8NF50;Q96N67	DOCK8
Q8TDX7	Q8TDX7;Q9HC98	NEK7
Q92925	Q92925;Q6STE5	BAF60B;PRO2451;SMARCD2
Q92945	Q92945;Q96AE4	FUBP2;KHSRP
Q93008	Q93008;O00507	DFFRX;FAM;USP9;USP9X
Q96BY6	Q96BY6;Q9BZ29	DOCK10;KIAA0694;ZIZ3
Q96GK7	Q96GK7;Q6P2I3	CGI-105;FAHD2A;FAHD2B
Q96J02	Q96J02;Q9H0M0	ITCH
Q96PY5	Q96PY5;Q8IVF7	FHOD2;FMNL2;KIAA1902
Q99873	Q99873;Q9NR22	HMT2;HRMT1L2;IR1B4;PRMT1
Q9BWF3	Q9BWF3;Q9BQ04	RBM4;RBM4A;RBM30;RBM4B
Q9H0U4	Q9H0U4;Q92928	RAB1B;RAB1C
Q9H4E7	Q9H4E7;Q6TDU7	DEF6;IBP
Q9NQL2	Q9NQL2;Q9HB90	RRAGD;RRAGC
Q9NNX6	Q9NNX6;Q9H2X3	CD209;CLEC4L;CD209L;CD209L1;CD299
Q9NP97	Q9NP97;Q8TF09	BITH;DNCL2A;DNLC2A;DYNLRB1;HSPC162
Q9NPQ8	Q9NPQ8;Q9NVN3	RIC8A
Q9NQT8	Q9NQT8;Q9H1H9	GAKIN;KIAA0639;KIF13B
Q9NR31	Q9NR31;Q9Y6B6	SAR1;SAR1A;SARA;SARA1
Q9NRR5	Q9NRR5;Q9UMX0	C1orf6;UBIN;UBQLN4;DA41;PLIC1;UBQLN1
Q9NUU7	Q9NUU7;Q9UHL0	DDX19A;DDX19L
Q9UKM9	Q9UKM9;Q86SE5	HNRPCL2;P542;RALY
Q9UL26	Q9UL26;Q13636	RAB22;RAB22A;RAB22B;RAB31
Q9UN37	Q9UN37;Q6PIW4	VPS4;VPS4A
Q9Y285	Q9Y285	FARS;FARSA;FARSL;FARSLA
Q9Y3L5	Q9Y3L5;Q8IXI1	RAP2C
Q9Y490	Q9Y490;Q9Y4G6	KIAA1027;TLN;TLN1
Q9Y4D7	Q9Y4D7;O60486	KIAA0620;PLXND1
Q3B8N2	Q3B8N2;Q6DKI2	LGALS9B;LGALS9C;LGALS9
O00425	O00425;Q9Y6M1	IGF2BP3;IMP3;KOC1;VICKZ3
O14950	O14950;P19105	MRLC2;MYL12B;MYLC2B;MLCB;MRLC3
O60333	O60333;Q12756	KIAA0591;KIAA1448;KIF1B;ATSV;C2orf20
P15531	P15531;P22392	NDPKA;NM23;NME1;NM23B;NME2;NME2P1
O95819	O95819;Q9UKE5	HGK;KIAA0687;MAP4K4;NIK;KIAA0551;TNIK
P00533	P00533;Q15303	EGFR;ERBB1
P01023	P01023;P20742	A2M;CPAMD5;FWP007
P05107	P05107;P18084	CD18;ITGB2;MFI7
P06313	P06313;P06312	IGKV4-1
P06733	P06733;P09104	ENO1;ENO1L1;MBPB1;MPB1
P06744	P06744;Q8N196	GPI
P08133	P08133	ANX6;ANXA6
P08134	P08134;P61586	ARH9;ARHC;RHOC;ARH12;ARHA;RHO12
P08238	P08238;Q58FF7	HSP90AB1;HSP90B;HSPC2;HSPCB
Q01628	Q01628;Q01629	IFITM3;IFITM2;CD225;IFI17;IFITM1
P13796	P13796;P13797	LCP1;PLS2
P18669	P18669;P15259	CDABP0006;PGAM1;PGAMA;PGAM2;PGAMM

P27986	P27986;O00459	GRB1;PIK3R1
P30483	P30483;P30487	HLAB;HLA-B
P33121	P33121;Q9UKU0	ACSL1;FACL1;FACL2;LACS;LACS1;LACS2
P33176	P33176;Q12840	KIF5B;KNS;KNS1
P35579	P35579;P35749	MYH9
P37235	P37235;P61601	BDR1;HPCAL1;NCALD;BDR2;HPCA
P41240	P41240;P42679	CSK
P42356	P42356;A4QPH2	PI4KA;PIK4;PIK4CA
P50502	P50502;Q8IZP2	FAM10A1;HIP;SNC6;ST13;FAM10A4;FAM10A5
P50570	P50570;Q9UQ16	DNM2;DYN2
P51114	P51114;P51116	FXR1
Q5JWF2	Q5JWF2;P63092	GNAS;GNAS1;GSP
P67809	P67809;P16989	NSEP1;YB1;YBX1
P68104	P68104;Q5VTE0	EEF1A;EEF1A1;EF1A;LENG7;EEF1AL3;EEF1A2
P68871	P68871;P02042	HBB;HBD
P78347	P78347;Q6EKJ0	BAP135;GTF2I;WBSCR6
P79483	P79483;P13761	HLA-DRB3;HLA-DRB1
Q13557	Q13557;Q13554	CAMK2D;CAMKD
Q14344	Q14344;Q03113	GNA13
Q14839	Q14839;Q8TDI0	CHD4
Q15365	Q15365;P57721	PCBP1
Q15717	Q15717;P26378	ELAVL1;HUR
Q15796	Q15796;O15198	MADH2;MADR2;SMAD2
Q16181	Q16181;Q92599	CDC10;SEPT7
Q63HN8	Q63HN8;Q70EK9	C17orf27;KIAA1554;RNF213
Q71U36	Q71U36;Q13748	TUBA1A;TUBA3;TUBA2;TUBA3C;TUBA3D
Q8NB66	Q8NB66;Q9UPW8	UNC13C;KIAA1032;UNC13A;UNC13;UNC13B
Q95365	Q95365;P30475	HLAB;HLA-B
Q9NUP9	Q9NUP9;O14910	LIN7C;MALS3;VELI3
Q12857	Q12857;P08651	KIAA1439;NFIA;NFI;NFIC;NFIX;NFIB
O75190	O75190;P25686	DNAJB6;HSJ2;MRJ;MSJ1
Q96E17	Q96E17;P20336	RAB3C;RAB3A;GOV;RAB16;RAB3D;RAB3B
P06310	P06310;P06309	
P01774	P01774;P01776	
P01857	P01857;P01861	IGHG1;IGHG4;IGHG2
P04439	P04439;P30443	HLAA;HLA-A
P04899	P04899;A8MTJ3	GNAI2;GNAI2B
P04908	P04908;Q7L7L0	H2AFA;H2AFM;HIST1H2AB;HIST1H2AE
P0CG05	P0CG05;P0CG06	IGLC2;IGLC3;IGLC7;IGLC6
P0CG48	P0CG48;P0CG47	RPS27A;UBA80;UBCEP1;UBA52;UBCEP2
P12814	P12814;O43707	ACTN1;ACTN4
P23634	P23634;P20020	ATP2B2;ATP2B4;MXRA1;ATP2B1;PMCA1
P30740	P30740;O75830	ELANH2;MNEI;PI2;SERPINB1
P42330	P42330;Q04828	AKR1C3;DDH1;HSD17B5;KIAA0119;PGFS
P68032	P68032;P62736	ACTC;ACTC1;ACTA2;ACTSA;ACTVS;GIG46
Q9H0C2	Q9H0C2;P05141	AAC4;ANT4;SFEC;SLC25A31;ANT2;SLC25A5

P05023	P05023;P13637	ATP1A1
P11940	P11940;Q9H361	PAB1;PABP1;PABPC1;PABPC2;PABP3;PABPC3
P19440	P19440;P36268	GGT;GGT1;GGT2;GGT3;GGT3P
P27361	P27361;Q16659	ERK1;MAPK3;PRKM3
P68431	P68431;Q71DI3	H3FA;H3FB;H3FC;H3FD;H3FF;H3FH;H3FI;H3FJ
P18135	P18135;P18136	
P06753	P06753;P09493	TPM3;C15orf13;TMSA;TPM1
P07900	P07900;Q14568	HSP90A;HSP90AA1;HSPC1;HSPCA
O75131	O75131;Q9UBL6	CPN3;CPNE3;KIAA0636
P01593	P01593;P01594	
P62820	P62820;P59190	RAB1;RAB1A
Q6S8J3	Q6S8J3;A5A3E0	A26C1A;POTE2;POTEE;A26C1B;POTEF;ACTBL3
P42285	P42285;O43151	KIAA0052;SKIV2L2
P06239	P06239;Q14004	LCK
