# Supplementary material for: Characterization of Human Thymic Exosomes
Source: PLoS One. 2013 Jul 2;8(7):e67554. doi: 10.1371/journal.pone.0067554 (PMC3699640; doi:10.1371/journal.pone.0067554)
Supplement: Table S2 — Shared proteins between the two individual exosomal samples. (PDF) [file pone.0067554.s003.pdf]

Uniprot	Gene	Gene syn	Cell type expression in HPA
A0AVT1	UBA6	FLJ10808	1/64
A4FU69	EFCAB5	FLJ46247	55/66
A5PLN9	C5orf44	FLJ13611	39/65
A6NHR9	SMCHD1	KIAA0650	47/64
B0I1T2	MYOG	HA-2	21/65
O00154	ACOT7	ACH1	35/65
O00159	MYO1C	myr2	59/66
O00161	SNAP23	HsT17016	47/64
O00186	STXBP3	UNC-18C	44/65
O00203	AP3B1	ADTB3A	48/64
O00231	PSMD11	MGC3844	19/62
O00232	PSMD12	p55	55/65
O00299	CLIC1	NCC27	45/64
O00410	IPO5	IMB3	45/63
O00443	PIK3C2A	PI3K-C2alpha	65/65
O00487	PSMD14	pad1	48/64
O00560	SDCBP	SYCL	64/65
O00571	DDX3X	DBX	45/66
O00764	PDXK	C21orf124	51/66
O14744	PRMT5	HRMT1L5	58/65
O14773	TPP1	CLN2	55/63
O14802	POLR3A	hRPC155	62/63
O14828	SCAMP3	C1orf3	18/66
O14920	IKBKB	IKK-beta	56/66
O14966	RAB7L1	RAB7L	61/66
O14976	GAK		53/65
O14980	XPO1	CRM1	54/65
O14981	BTAF1	MOT1	55/66
O15111	CHUK	IkBKA	66/66
O15143	ARPC1B	ARC41	23/66
O15144	ARPC2	ARC34	66/66
O15145	ARPC3	ARC21	54/65
O15258	RER1		64/65
O15294	OGT	FLJ23071	56/66
O15305	PMM2	CDG1	43/63
O15372	EIF3H	eIF3-gamma	65/65
O15397	IPO8	IMP8	40/66
O15400	STX7		27/63
O43172	PRPF4	HPRP4	64/64
O43175	PHGDH	PDG	40/63
O43242	PSMD3	P58	64/65
O43301	HSPA12A	FLJ13874	47/65
O43324	EEF1E1	AIMP3	57/66
O43390	HNRNPR	hnRNP-R	66/66
O43396	TXNL1	TRP32	66/66

O43488	AKR7A2	AFAR	55/65
O43491	EPB41L2	4.1-G	55/64
O43493	TGOLN2	TGN38	64/64
O43561	LAT	LAT1	8/64
O43617	TRAPPC3	BET3	48/63
O43684	BUB3	BUB3L	62/64
O43865	AHCYL1	IRBIT	22/65
O60256	PRPSAP2	PAP41	50/64
O60264	SMARCA5	hISWI	64/66
O60449	LY75	CD205	28/63
O60488	ACSL4	ACS4	44/63
O60496	DOK2	Dok-2	9/64
O60547	GMDS	GMD	62/64
O60645	EXOC3	SEC6L1	10/65
O60701	UGDH		31/64
O60716	CTNND1	CTNND	50/65
O60942	RNGTT	hCAP	41/64
O75083	WDR1		54/62
O75131	CPNE3		52/65
O75165	DNAJC13	KIAA0678	61/64
O75190	DNAJB6	MRJ	21/65
O75369	FLNB	ABP-278	46/66
O75475	PSIP1	LEDGF	64/64
O75531	BANF1	BAF	65/65
O75534	CSDE1	D1S155E	55/66
O75582	RPS6KA5	MSK1	55/66
O75592	MYCBP2	FLJ10106	24/64
O75643	SNRNP200	ASCC3L1	51/65
O75676	RPS6KA4	MSK2	35/65
O75695	RP2	NME10	41/65
O75792	RNASEH2A	AGS4	49/65
O75828	CBR3	SDR21C2	63/64
O75955	FLOT1		51/64
O76094	SRP72		64/64
O94776	MTA2	MTA1-L1	65/65
O94973	AP2A2	ADTAB	59/62
O95155	UBE4B	E4	52/66
O95163	IKBKAP	DYS	20/64
O95197	RTN3	ASYIP	13/66
O95352	ATG7	APG7L	64/64
O95399	UTS2	PRO1068	48/65
O95466	FMNL1	C17orf1	10/66
Q96E17	RAB3C		16/65
O95747	OXSR1	KIAA1101	62/65
O95782	AP2A1	ADTAA	59/66
O95786	DDX58	DKFZp434J1111	52/66

O95865	DDAH2		63/63
O96019	ACTL6A	Actl6	65/65
P00338	LDHA		55/65
P00352	ALDH1A1	ALDH1	30/66
P00374	DHFR		59/66
P00387	CYB5R3	DIA1	58/65
P00450	CP		35/66
P00558	PGK1		52/64
P00813	ADA		58/66
P01009	SERPINA1	A1A	12/66
P01011	SERPINA3	AACT	32/65
P01023	A2M	CPAMD5	4/64
P01111	NRAS	N-ras	62/62
P01591	IGJ	IGCJ	22/62
P01730	CD4		6/66
P01732	CD8A	CD8	9/65
P02511	CRYAB	CRYA2	17/66
P02647	APOA1		2/64
P02649	APOE	AD2	48/64
P02671	FGA		21/64
P02675	FGB		30/66
P02679	FGG		7/65
P02751	FN1	CIG	9/66
P02786	TFRC	CD71	49/66
P02787	TF	PRO1557	58/66
P02788	LTF	HLF2	40/65
P02790	HPX		14/64
P02792	FTL	MGC71996	40/64
P02794	FTH1	FHC	61/64
P04004	AC002094.1		
P04075	ALDOA		48/65
P04083	ANXA1	ANX1	29/65
P04216	THY1	CD90	7/64
P04233	CD74	DHLAG	45/66
P04234	CD3D	T3D	18/65
P04275	VWF	F8VWF	1/66
P04350	TUBB4	beta-5	55/63
P04440	HLA-DP1B	HLA-DP1B	43/65
P04632	CAPNS1	30K	63/65
P04792	HSPB1	Hs.76067	45/66
P04839	CYBB	CGD	59/66
P04843	RPN1	OST1	47/66
P04899	GNAI2	GIP	60/60
P04908	HIST1H2AB	H2A/m	65/65
P05023	ATP1A1		39/66
P05026	ATP1B1		56/66

P05107	ITGB2	CD18	15/66
P05164	MPO		7/66
P05198	EIF2S1	EIF-2alpha	65/66
P05362	ICAM1	BB2	16/65
P05388	RPLP0	L10E	39/63
P05556	ITGB1	CD29	65/65
P06126	CD1A	CD1	4 of 65
P06127	CD5	LEU1	8 of 65
P06239	LCK		9 of 65
P06241	FYN	MGC45350	20/66
P06396	GSN	DKFZp313L0718	35/64
P06493	CDK1	CDC2	38/66
P06729	CD2	SRBC	7/65
P06730	EIF4E	EIF4E1	65/65
P06733	ENO1	ENO1L1	60/65
P06744	GPI	AMF	59/66
P06753	TPM3	NEM1	5/65
P07099	EPHX1	EPHX	38/66
P07195	LDHB		57/64
P07203	GPX1		25/63
P07237	P4HB	DSI	53/66
P07305	H1F0	H10	64/64
P07339	CTSD	CLN10	58/66
P07355	ANXA2	ANX2	48/65
P07437	TUBB	M40	65/65
P07737	PFN1		61/65
P07741	APRT		31/66
P07766	CD3E		6/66
P07814	EPRS	EARS	62/65
P07858	CTSB		64/66
P07900	HSP90AA1	FLJ31884	17/66
P07910	HNRNPC	hnRNPC	62/62
P07947	YES1	c-yes	66/66
P08107	HSPA1A	HSP70-1	62/64
P08133	ANXA6	ANX6	44/66
P08134	RHOC	ARH9	6/65
P08237	PFKM	PFK-1	64/65
P08238	HSP90AB1	HSPC2	63/63
P08246	ELANE	ELA2	1/66
P08311	CTSG	CG	5/65
P08575	PTPRC	CD45	9/66
P08631	HCK	JTK9	45/62
P08670	VIM		35/65
P08754	GNAI3	87U6	24/63
P08758	ANXA5	ANX5	42/63
P08865	RPSA	37LRP	63/64

P09211	GSTP1	FAEES3	52/66
P09326	CD48	BCM1	3/65
P09496	CLTA	Lca	45/57
P09497	CLTB	Lcb	58/63
P09525	ANXA4	ANX4	40/65
P09543	CNP		4/66
P09874	PARP1	ADPRT	65/66
P0C0S5	H2AFZ	H2A.Z	61/61
P0CG48	UBC		64/64
P10114	RAP2A	K-REV	65/65
P10301	RRAS		63/63
P10412	HIST1H1E	H1.4	64/64
P10644	PRKAR1A	PRKAR1	47/66
P10768	ESD		43/64
P10909	CLU	APOJ	21/65
P10966	CD8B	CD8B1	64/65
P11021	HSPA5	BiP	66/66
P11142	HSPA8	HSC70	40/64
P11171	EPB41	4.1R	0/66
P11172	UMPS		60/65
P11234	RALB		31/63
P11279	LAMP1	CD107a	65/65
P11387	TOP1		65/66
P11388	TOP2A	TOP2	41/66
P11413	G6PD	G6PD1	35/66
P11586	MTHFD1	MTHFC	60/66
P11717	IGF2R	CD222	47/64
P11940	PABPC1	PAB1	60/66
P12004	PCNA		45/65
P12268	IMPDH2		65/66
P12956	XRCC6	D22S671	61/62
P13010	XRCC5	KARP-1	65/65
Q01628	IFITM3	1-8U	34/65
P13639	EEF2	EEF-2	63/63
P13667	PDIA4	ERP70	63/65
P13796	LCP1	CP64	10/66
P13807	GYS1	GSY	63/65
P13861	PRKAR2A	PRKAR2	63/66
P14209	CD99	MIC2	9/66
P14618	PKM2	OIP3	52/66
P14625	HSP90B1	GP96	60/60
P14866	HNRNPL	HNRPL	66/66
P14868	DARS		63/66
P14923	JUP	CTNNG	34/66
P15121	AKR1B1	ALDR1	66/66
P15144	ANPEP	CD13	16/64

P15153	RAC2	EN-7	30/64
P15311	EZR	VIL2	56/66
P15498	VAV1	VAV	54/65
P15927	RPA2		65/65
P16070	CD44	CD44R	33/66
P16104	H2AFX	H2AX	65/65
P16152	CBR1	CBR	55/66
P16401	HIST1H1B	H1.5	45/66
P16403	HIST1H1C	H1.2	66/66
P16455	MGMT		46/62
P17301	ITGA2	CD49B	41/65
P17655	CAPN2	CANPL2	27/66
P17812	CTPS		63/63
P17844	DDX5	G17P1	66/66
P17858	PFKL		32/64
P17980	PSMC3	TBP-1	62/62
P18031	PTPN1	PTP1B	54/66
P18754	RCC1	CHC1	66/66
P19338	NCL	C23	66/66
P19387	POLR2C	RPB3	59/63
P19623	SRM	SPS1	59/65
P19971	TYMP	ECGF1	28/65
P20073	ANXA7	ANX7	58/64
P20339	RAB5A	RAB5	65/65
P20340	RAB6A	RAB6	59/64
P20591	MX1	IFI-78K	64/65
P20592	MX2	MXB	42/65
P20618	PSMB1	HC5	63/66
P20645	M6PR		64/64
P20701	ITGAL	CD11A	10/65
P20839	IMPDH1	LCA11	61/64
P21281	ATP6V1B2	ATP6B2	51/65
P21333	FLNA	ABP-280	26/66
P21359	NF1		59/65
P21399	ACO1	IREB1	38/66
P21589	NT5E	CD73	64/64
P21926	CD9	BA2	45/62
P22087	FBL	FIB	48/63
P22102	GART	PGFT	59/64
P22234	PAICS	ADE2H1	62/63
P22314	UBA1	A1S9T	66/66
P22626	HNRNPA2B1	HNRPA2B1	64/64
P22694	PRKACB	PKACb	66/66
P23246	SFPQ	PSF	61/63
P23258	TUBG1	TUBG	62/62
P23284	PPIB	CYPB	47/62

P23458	JAK1	JAK1A	50/61
P23528	CFL1	CFL	54/63
P23634	ATP2B4	ATP2B2	54/64
P23921	RRM1		43/65
P24666	ACP1		50/65
P24928	LC35G6	AMAC1L3	66/66
P24941	CDK2		34/65
P25205	MCM3		26/66
P25705	ATP5A1	ATP5A	66/66
P25786	PSMA1	HC2	65/65
P25787	PSMA2	HC3	59/65
P25788	PSMA3	HC8	37/64
P25789	PSMA4	HC9	52/64
P26038	MSN		44/66
P26196	DDX6	HLR2	65/66
P26599	PTBP1	HNRNP-I	54/63
P26641	EEF1G	EF1G	53/66
P27105	STOM	BND7	12/65
P27348	YWHAQ	HS1	57/64
P27361	MAPK3	ERK1	66/66
P27482	CALML3	CLP	52/64
P27635	RPL10	DXS648	66/66
P27694	RPA1	HSSB	65/65
P27701	CD82	IA4	9/65
P27708	CAD		52/62
P27797	CALR	cC1qR	48/66
P27824	CANX	CNX	65/65
P27986	PIK3R1	GRB1	62/64
P28065	PSMB9	beta1i	16/64
P28066	PSMA5	ZETA	65/65
P28070	PSMB4	HN3	51/63
P28072	PSMB6	DELTA	65/65
P28340	POLD1	CDC2	55/64
P28482	MAPK1	ERK	66/66
P28838	LAP3	LAP	43/65
P28907	CD38		9/66
P29016	CD1B	CD1	41/63
P29144	TPP2		62/63
P29728	OAS2		66/66
P29966	MARCKS	80K-L	53/63
P30040	ERP29	64/64	
P30041	PRDX6	1-CYS	65/65
P30043	BLVRB	FLR	64/64
P30050	RPL12	L12	49/64
P30101	PDIA3	ERp57	62/65
P30153	PPP2R1A	PP2A-Aalpha	51/63

P30740	SERPINB1	anti-elastase	16/61
P31150	GDI1	FLJ41411	57/65
P31689	DNAJA1	dj-2	52/64
P31942	HNRNPH3	2H9	10/63
P31943	HNRNPH1	hnRNPH	65/65
P31946	YWHAB	YWHAA	64/65
P31947	SFN	YWHAS	14/66
P32119	PRDX2	MGC4104	61/66
P32942	ICAM3	CD50	11/64
P32969	RPL9	L9	50/64
P33121	ACSL1	ACS1	50/65
P33176	KIF5B	KNS	55/60
P33991	MCM4	CDC21	65/65
P33992	MCM5	CDC46	30/66
P33993	MCM7	CDC47	39/66
P34896	SHMT1	CSHMT	54/65
P35221	CTNNA1	CAP102	49/61
P35241	RDX	DFNB24	37/66
P35249	RFC4	A1	48/59
P35579	MYH9	DFNA17	45/66
P35580	MYH10	NMMHCB	48/65
P35606	COPB2	beta'-COP	53/64
P35613	BSG	CD147	37/61
P36405	ARL3	ARFL3	60/64
P36507	MAP2K2	MEK2	57/64
P36543	ATP6V1E1	ATP6E	57/66
P36578	RPL4	L4	41/65
P36873	PPP1CC	PP1gamma	49/63
P36969	GPX4	MCSP	50/65
P37802	TAGLN2	HA1756	4/61
P37837	TALDO1		63/64
P38606	ATP6V1A	ATP6A1	58/61
P38919	EIF4A3	DDX48	65/65
P39023	RPL3	L3	58/64
P40306	PSMB10	beta2i	36/65
P40763	STAT3	APRF	53/65
P40925	MDH1		22/66
P40937	RFC5	RFC36	51/63
P40938	RFC3	MGC5276	60/66
P41091	EIF2S3	EIF2	57/63
P41218	MNDA	PYHIN3	7/65
P41226	UBA7	D8	53/66
P41240	CSK		16/66
P41250	GARS	CMT2D	65/66
P41252	IARS	IARS1	29/65
P42224	STAT1	ISGF-3	37/66

P42226	STAT6	D12S1644	49/63
P42229	STAT5A	MGF	21/66
P42338	PIK3CB	PIK3C1	62/65
P42345	MTOR	FLJ44809	9/64
P42356	PI4KA	PI4K-ALPHA	35/66
P43034	PAFAH1B1	LIS1	66/66
P43243	MATR3	KIAA0723	66/66
P43246	MSH2	COCA1	58/64
P43403	ZAP70	SRK	8/66
P43405	SYK		43/66
P43490	NAMPT	PBEF	48/63
P43686	PSMC4	MGC13687	63/64
P45973	CBX5	HP1	56/65
P45974	USP5	IsoT	56/65
P46020	PHKA1	PHKA	33/65
P46063	RECQL	RecQ1	60/64
P46459	NSF	SKD2	66/66
P46734	MAP2K3	MAPKK3	51/66
P46940	IQGAP1	HUMORFA01	54/65
P47755	CAPZA2	CAPPA2	46/61
P47756	CAPZB		57/66
P47897	QARS		50/65
P48444	ARCN1	COPD	59/64
P48556	PSMD8	HIP6	59/66
P48643	CCT5	KIAA0098	53/65
P49005	POLD2		47/66
P49006	MARCKSL1	F52	41/65
P49189	ALDH9A1	ALDH4	63/64
P49207	RPL34	L34	54/65
P49257	LMAN1	ERGIC-53	50/62
P49327	FASN	FAS	48/64
P49368	CCT3	Cctg	64/64
P49588	AARS		58/65
P49591	SARS	SERS	55/65
P49721	PSMB2	HC7-I	61/65
P49736	MCM2	BM28	39/65
P49755	TMED10	P24(DELTA)	60/65
P49841	GSK3B		10/66
P50148	GNAQ	G-ALPHA-q	62/62
P50224	SULT1A3	STM	63/63
P50552	VASP		63/65
P50914	RPL14	CTG-B33	65/65
P50990	CCT8	C21orf112	62/66
P50991	CCT4	Cctd	32/66
P50995	ANXA11	ANX11	65/65
P51114	FXR1		63/66

P51148	RAB5C	RAB5CL	57/62
P51149	RAB7A	RAB7	65/65
P51151	RAB9A	RAB9	58/65
P51153	RAB13		31/63
P51178	PLCD1		53/65
P51532	SMARCA4	BAF190	62/63
P51570	GALK1	GALK	58/65
P51572	BCAP31	6C6-Ag	66/66
P51665	PSMD7	MOV34	64/65
P51812	RPS6KA3	CLS	62/62
P51991	HNRNPA3	HNRPA3	63/63
P52272	HNRNPM	CEAR	65/65
P52564	MAP2K6	MAPKK6	58/66
P52565	ARHGDIA	GDIA1	66/66
P52566	ARHGDIB	GDIA2	20/66
P52597	HNRNPF	HNRPF	62/64
P52630	STAT2	STAT113	37/66
P52701	MSH6	GTBP	63/66
P53004	BLVRA	BLVR	60/65
P53396	ACLY	ACL	66/66
P53618	COPB1	COPB	62/62
P53621	COPA	HEP-COP	61/65
P54136	RARS	DALRD1	40/65
P54652	HSPA2		62/65
P54709	ATP1B3	CD298	62/63
P54920	NAPA		66/66
P55036	PSMD4	AF	58/65
P55039	DRG2		60/63
P55060	CSE1L	CAS	34/66
P55072	VCP	IBMPFD	65/65
P55209	NAP1L1	MGC23410	61/64
P55263	ADK	AK	49/63
P55265	ADAR	ADAR1	32/62
P55795	HNRNPH2	FTP3	65/65
P55884	EIF3B	eIF3b	64/64
P56192	MARS	MetRS	66/66
P56589	PEX3		59/65
P57735	RAB25	CATX-8	49/65
P57737	CORO7	FLJ22021	37/64
P60033	CD81	TAPA-1	17/66
P60174	TPI1		57/65
P60228	EIF3E	eIF3-p48	57/64
P60842	EIF4A1	DDX2A	32/63
P60866	RPS20	S20	61/65
P60900	PSMA6	IOTA	59/65
P61018	RAB4B	CD-RAP	36/65

P61019	RAB2A	RAB2	63/63
P61020	RAB5B		63/64
P61081	UBE2M	hUbc12	58/63
P61106	RAB14	FBP	47/65
P61158	ACTR3	ARP3	62/64
P61160	ACTR2	ARP2	48/65
P61163	ACTR1A	ARP1	42/61
P61313	RPL15	EC45	22/65
P61353	RPL27	L27	59/63
P61769	B2M		65/66
P61978	HNRNPK	CSBP	64/64
P61981	YWHAG		66/66
P62070	RRAS2	TC21	64/64
P62136	PPP1CA	PP-1A	42/62
P62140	PPP1CB	PP-1B	34/62
P62158	CALM1	CALML2	64/64
P62191	PSMC1	p56	61/66
P62195	PSMC5	p45	64/65
P62241	RPS8	S8	23/66
P62258	YWHAE	FLJ45465	66/66
P62263	RPS14	EMTB	64/66
P62330	ARF6		51/66
P62701	RPS4X	CCG2	61/66
P62714	PPP2CB	PP2Abeta	64/64
P62753	RPS6	S6	65/66
P62820	RAB1A	RAB1	65/65
P62829	RPL23	L23	62/65
P62834	RAP1A	KREV-1	52/64
P62847	RPS24	S24	28/64
P62913	RPL11	L11	51/65
P62937	PPIA	CYPA	50/65
P63000	RAC1	p21-Rac1	27/65
P63010	AP2B1	ADTB2	48/62
Q5JWF2	GNAS	GNAS1	6/66
P63096	GNAI1		50/64
P63104	YWHAZ	14-3-3-zeta	64/64
P63208	SKP1	EMC19	60/66
P63244	GNB2L1	Gnb2-rs1	54/65
P67775	PPP2CA	PP2Calpha	60/62
P67809	YBX1	BP-8	65/65
P67870	CSNK2B		66/66
P68032	ACTC1	ACTC	15/66
P68366	TUBA4A	FLJ30169	66/66
P68400	CSNK2A1		59/62
P68871	HBB	beta-globin	19/66
P69892	HBG2		5/66

P69905	HBA1		11/66
P78347	GTF2I	BAP-135	65/65
P78371	CCT2	Cctb	51/65
P78417	GSTO1	GSTTLp28	14/65
P83916	CBX1	CBX	64/64
P84095	RHOG	ARHG	20/64
P84103	SRSF3	SFRS3	66/66
P98194	ATP2C1	ATP2C1A	63/65
Q00341	HDLBP	HBP	51/66
Q00535	CDK5	PSSALRE	66/66
Q00610	CLTC	CLTCL2	61/65
Q00765	REEP5	C5orf18	60/63
Q00839	HNRNPU	hnRNPU	64/64
Q01082	SPTBN1		65/65
Q01518	CAP1	CAP	51/65
Q01826	SATB1		13/61
Q02750	MAP2K1	MAPKK1	61/66
Q02790	FKBP4	FKBP52	56/64
Q02818	NUCB1	Calnuc	53/63
Q02880	TOP2B		66/66
Q03169	TNFAIP2	B94	29/62
Q04917	YWHAH	YWHA1	50/65
Q05707	COL14A1	UND	34/65
Q06323	PSME1	IFI5111	59/66
Q06830	PRDX1	NKEFA	61/65
Q07075	ENPEP	CD249	35/64
Q07954	LRP1	A2MR	26/66
Q07955	SRSF1	ASF	65/65
Q08211	DHX9	DDX9	65/65
Q08257	CRYZ		58/65
Q08380	LGALS3BP	90K	29/66
Q08722	CD47	IAP	28/66
Q08945	SSRP1	FACT80	34/64
Q08AF3	SLFN5	MGC19764	55/65
Q08AM6	VAC14	ArPIKfyve	42/66
Q08J23	NSUN2	FLJ20303	58/63
Q09028	RBBP4	NURF55	54/62
Q09666	AHNAK	MGC5395	59/66
Q10589	BST2	CD317	51/61
Q12846	STX4	p35-2	55/64
Q12874	SF3A3	PRP9	66/66
Q12904	AIMP1	EMAP-2	65/65
Q12905	ILF2	NF45	63/63
Q12906	ILF3	DRBP76	65/65
Q12907	LMAN2	C5orf8	22/64
Q13043	STK4	KRS2	65/65

Q13045	FLII	FLI	59/63
Q13057	COASY	CoASY	63/66
Q13077	TRAF1	EBI6	12/65
Q13131	AC008810.1		60/63
Q13148	TARDBP	ALS10	65/65
Q13151	HNRNPA0	hnRNPA0	42/64
Q13155	AIMP2	JTV-1	58/65
Q13185	CBX3	HP1Hs-gamma	66/66
Q13263	TRIM28	KAP1	59/63
Q13303	KCNAB2	AKR6A5	23/66
Q13308	PTK7	CCK4	46/64
Q13347	EIF3I	eIF3-beta	66/66
Q13363	CTBP1	BARS	66/66
Q13395	TARBP1	TRM3	38/65
Q13409	DYNC1I2	DNCI2	60/65
Q13418	ILK		44/63
Q13451	FKBP5	FKBP51	60/64
Q13464	ROCK1	p160ROCK	52/66
Q13501	SQSTM1	A170	61/64
Q13509	TUBB3	beta-4	13/66
Q13547	HDAC1	GON-10	65/65
Q13561	DCTN2	DCTN-50	64/64
Q13576	IQGAP2		29/65
Q13630	TSTA3	FX	23/65
Q13637	RAB32		49/65
Q13733	ATP1A4	ATP1AL2	40/64
Q13813	SPTAN1		65/65
Q13885	TUBB2A	dJ40E16.7	42/64
Q14103	HNRNPD	AUF1	59/64
Q14141		KIAA0128	41/61
Q14152	EIF3A	EIF3	63/65
Q14195	DPYSL3	CRMP4	25/64
Q14203	DCTN1		57/64
Q14204	DYNC1H1	DHC1	53/66
Q14240	EIF4A2	BM-010	64/64
Q14258	TRIM25	EFP	47/61
Q14344	GNA13	G13	55/65
Q14376	GALE	SDR1E1	61/64
Q14498	RBM39	CAPER	62/65
Q14566	MCM6	Mis5	48/65
Q14683	SMC1A	DXS423E	62/65
Q14697	GANAB	G2AN	53/66
Q14764	MVP	LRP	45/66
Q14839	CHD4	Mi-2b	65/66
Q14974	KPNB1	IMB1	65/65
Q14980	NUMA1		65/65

Q15008	PSMD6	KIAA0107	58/65
Q15019		DIFF6	61/66
Q15021	NCAPD2	CAP-D2	66/66
Q15029	EFTUD2	Snrp116	65/65
Q15046	KARS	KARS2	64/64
Q15080	NCF4	p40phox	7/64
Q15084	PDIA6	ERp5	57/64
Q15102	PAFAH1B3		25/65
Q15111	PLCL1	PLC-L	63/65
Q15126	PMVK	HUMPMKI	63/65
Q15149	PLEC	EBS1	66/66
Q15181	PPA1	IOPPP	62/66
Q15233	NONO	NMT55	64/64
Q15283	RASA2	GAP1M	36/66
Q15363	TMED	P24A	60/64
Q15365	PCBP1	hnRNP-E1	63/64
Q15366	PCBP2	hnRNP-E2	64/65
Q15382	RHEB	RHEB2	43/65
Q15386	UBE3C	KIAA0010	48/64
Q15404	RSU1	FLJ31034	26/65
Q15418	RPS6KA1	HU-1	60/65
Q15459	SF3A1	Prp21	65/65
Q15582	TGFBI	BIGH3	12/65
Q15628	TRADD	Hs.89862	42/63
Q15645	TRIP13	16E1BP	28/65
Q15717	ELAVL1	Hua	61/65
Q15773	MLF2	NTN4	65/66
Q15796	SMAD2	JV18-1	65/65
Q16181		CDC10	59/64
Q16186	ADRM1	ARM1	60/63
Q16401	PSMD5	KIAA0072	61/63
Q16531	DDB1	XPE	56/66
Q16555	DPYSL2	CRMP2	66/66
Q16563	SYPL1	SYPL	49/65
Q16566	CAMK4	CaMK-GR	34/66
Q16576	RBBP7	RbAp46	64/64
Q16637	SMN1	BCD541	46/66
Q16643	DBN1	D0S117E	24/66
Q16658	FSCN1	FLJ38511	37/65
Q16666	IFI16	IFNGIP1	24/65
Q16698	DECR1	DECR	66/66
Q16706	MAN2A1	GOLIM7	52/66
Q16851	UGP2		13/65
Q29RF7	PDS5A	KIAA0648	65/65
Q2NL82	TSR1	FLJ10534	42/64
Q460N5	PARP14	KIAA1268	66/66

Q5K651	SAMD9	C7orf5	66/66
Q5SSJ5	HP1BP3	HP1-BP74	62/66
Q5T447	HECTD3	FLJ21156	58/64
Q5T4S7	UBR4	KIAA0462	46/64
Q5TZA2	CROCC	ROLT	5/66
Q5VIR6	VPS53	FLJ10979	34/66
Q5VW38	GPR107	FLJ20998	63/64
Q5VZM2	RRAGB		49/64
Q63HN8	RNF213	C17orf27	58/66
Q658Y4	FAM91A1	FLJ23790	45/65
Q6F5E8	RLTPR	CARMIL2	45/62
Q6I9Y2	THOC7	FLJ23445	59/63
Q6N069	NAA16	FLJ22054	43/63
Q6NXE6	ARMC6	MGC19595	46/64
Q6P2Q9	PRPF8	Prp8	58/65
Q6PGP7	TTC37	KIAA0372	51/64
Q6PIZ9	TRAT1	HSPC062	4/66
Q6UB35	MTHFD1L	DKFZP586G1517	57/64
Q6ZS81	WDFY4	C10orf64	62/66
Q71U36	TUBA1A	B-ALPHA-1	57/65
Q7KZF4	SND1	p100	64/64
Q7L014	DDX46	FLJ25329	63/64
Q7L2E3	DHX30	DDX30	46/64
Q7L2H7	EIF3M	eIF3m	52/65
Q7L5D6	GET4	C7orf20	63/66
Q7Z408	CSMD2	KIAA1884	54/63
Q7Z7H5	TMED4	HNLF	59/63
Q7Z7K6	CENPV	CENP-V	53/65
Q7Z7L1	SLFN11	FLJ34922	31/64
Q86TI0	TBC1D1	KIAA1108	43/63
Q86V21	AACS	ACSF1	13/65
Q86Y56	HEATR2	FLJ20397	12/65
Q86Y82	STX12	STX13	54/64
Q8IUR7	ARMC8	DKFZP434A043	55/64
Q8N163	KIAA1967	DBC-1	60/64
Q8N1F7	NUP93	KIAA0095	45/66
Q8N1G4	LRRC47	KIAA1185	64/65
Q8N1K5	THEMIS	bA325O24.3	54/64
Q8N3C0	ASCC3	ASC1p200	66/66
Q8N5D0	WDTC1	ADP	54/66
Q8N684	CPSF7	FLJ12529	64/64
Q8N8A2	ANKRD44	PP6-ARS-B	24/65
Q8NB90	SPATA5	AFG2	43/63
Q8ND71	GIMAP8	DKFZp667I133	66/66
Q8NF50	DOCK8	FLJ00026	39/61
Q8TAQ2	SMARCC2	BAF170	66/66

Q8TCG1	KIAA1524		58/63
Q8TD19	NEK9	DKFZp434D0935	30/61
Q8TDX7	NEK7		55/66
Q8TDZ2	MICAL1	DKFZp434B1517	15/65
Q8TEQ6	GEMIN5		63/64
Q8TF42	UBASH3B	KIAA1959	57/64
Q8WUM4	PDCD6IP	AIP1	51/66
Q8WVT3	TTC15	CGI-87	66/66
Q8WXX0	DNAH7	KIAA0944	44/63
Q8WYJ6		DIFF6	13/64
Q92499	DDX1	DBP-RB	64/64
Q92522	H1FX	H1X	62/63
Q92556	ELMO1	CED-12	27/66
Q92597	NDRG1	CAP43	61/65
Q92608	DOCK2	KIAA0209	25/68
Q92614	MYO18A	KIAA0216	59/66
Q92616	GCN1L1	GCN1	61/66
Q92769	HDAC2	RPD3	65/65
Q92841	DDX17	P72	41/64
Q92878	RAD50	hRad50	65/65
Q92879	CELF1	BRUNOL2	66/66
Q92888	ARHGEF1	LBCL2	36/66
Q92896	GLG1	CFR-1	64/65
Q92900	UPF1	HUPF1	59/66
Q92922	SMARCC1	BAF155	65/65
Q92945	KHSRP	FBP2	63/64
Q92973	TNPO1	IPO2	63/63
Q92974	ARHGEF2	GEF-H1	51/66
Q93008	USP9X	DFFRX	57/65
Q93009	USP7	HAUSP	65/66
Q969G3	SMARCE1	BAF57	64/65
Q969P0	IGSF8	CD316	7/62
Q96A08	HIST1H2BA	bA317E16.3	24/65
Q96A65	EXOC4	KIAA1699	53/65
Q96BY6	DOCK10	KIAA0694	5/64
Q96CW1	AP2M1	AP50	42/65
Q96D46	NMD3	CGI-07	24/64
Q96DI7	SNRNP40	HPRP8BP	66/66
Q96FV9	THOC1	HPR1	49/65
Q96FW1	OTUB1	FLJ20113	53/64
Q96FZ7	CHMP6	FLJ11749	65/65
Q96IJ6	GMPPA		25/66
Q96IV0	NGLY1	FLJ11005	53/61
Q96JI7	SPG11	FLJ21439	63/65
Q96JJ3	ELMO2	CED-12	31/65
Q96PK6	RBM14	COAA	63/64

Q96PY5	FMNL2	FHOD2	64/64
Q96S44	TP53RK	BUD32	66/66
Q96S59	RANBP9	RanBPM	44/66
Q96S99	PLEKHF1	APPD	17/65
Q99426	TBCB	CG22	45/65
Q99447	PCYT2	ET	58/66
Q99460	PSMD1	P112	62/62
Q99613	EIF3C	eIF3-p110	47/65
Q99715	COL12A1	COL12A1L	43/66
Q99733	NAP1L4	NAP2	60/65
Q99832	CCT7	Ccth	65/65
Q99836	MYD88		65/65
Q99873	PRMT1	ANM1	45/63
Q99973	TEP1	p240	46/65
Q9BPX3	NCAPG	CAP-G	47/66
Q9BQE5	APOL2	APOL-II	58/65
Q9BRX2	PELO		52/65
Q9BRX8	C10orf58	MGC4248	61/65
Q9BSD7	NTPCR	C1orf57	33/65
Q9BSJ2	TUBGCP2	GCP2	56/63
Q9BT78	COPS4	CSN4	62/64
Q9BTC8	MTA3	KIAA1266	57/64
Q9BUL8	PDCD10	CCM3	64/66
Q9BUP3	HTATIP2	CC3	66/66
Q9BUT1	BDH2	DHRS6	47/64
Q9BV86	METTL11A	AD-003	50/66
Q9BVC4	MLST8	GbetaL	35/65
Q9BVC6	TMEM109	MGC5508	63/64
Q9BVK6	TMED9	HSGP25L2G	59/63
Q9BXJ9	NAA15	FLJ13340	52/66
Q9BY32	ITPA	C20orf37	47/63
Q9BZH6	WDR11	BRWD2	50/64
Q9BZQ8	FAM129A	C1orf24	32/66
Q9GZN7	ROGDI	FLJ22386	55/64
Q9GZP4	PITHD1	C1orf128	31/61
Q9GZS3	WDR61	REC14	65/66
Q9H0J9	PARP12	FLJ22693	53/66
Q9H0Q0	FAM49A	DKFZP566A1524	65/65
Q9H0U4	RAB1B		59/64
Q9H2U2	PPA2	FLJ20459	59/64
Q9H400	LIME1	dJ583P15.4	47/64
Q9H4E7	DEF6	IBP	13/63
Q9H4G4	GLIPR2	C9orf19	58/66
Q9H7D0	DOCK5	FLJ21034	14/63
Q9H8W4	PLEKHF2	FLJ13187	45/65
Q9HAV0	GNB4		66/66

Q9HAV4	XPO5	KIAA1291	66/66
Q9HBI0	PARVG		45/64
Q9HC35	EML4	C2orf2	56/65
Q9HCF4	RNF213	C17orf27	58/66
Q9NP72	RAB18		51/65
Q9NPD3	EXOSC4	FLJ20591	60/65
Q9NPF4	OSGEP	GCPL1	35/63
Q9NQC3	RTN4	ASY	32/64
Q9NQE7	PRSS16	TSSP	22/65
Q9NQT8			
Q9NR31	SAR1A	SAR1	66/66
Q9NR45	NANS	SAS	48/63
Q9NRF8	CTPS2		64/64
Q9NSD9	FARSB	FARSLB	28/65
Q9NTI5	PDS5B	APRIN	64/64
Q9NTJ3	SMC4	CAP-C	54/66
Q9NU22	MDN1	KIAA0301	36/66
Q9NUQ9	FAM49B	BM-009	53/64
Q9NUV9	GIMAP4	FLJ11110	11/66
Q9NVA2		FLJ10849	41/61
Q9NVE7	PANK4	FLJ10782	65/65
Q9NVI1	FANCI	FLJ10719	57/63
Q9NW64	RBM22	Cwc2	62/62
Q9NZB2	FAM120A	C9orf10	66/66
Q9NZL9	MAT2B	MATIIbeta	31/65
Q9P016	THYN1	THY28	65/65
Q9P0M6	H2AFY2	macroH2A2	66/66
Q9P253	VPS18	KIAA1475	37/63
Q9P2B2	PTGFRN	CD315	41/64
Q9P2J5	LARS	FLJ10595	48/66
Q9P2R3	ANKFY1	ANKHZN	31/65
Q9UBN7	HDAC6	FLJ16239	52/64
Q9UBQ0	VPS29	DC15	62/65
Q9UBQ7	GRHPR	GLXR	44/65
Q9UBU9	NXF1	DKFZp667O0311	64/64
Q9UG63	ABCF2	ABC28	62/66
Q9UHB9	SRP68		51/65
Q9UI08	EVL	RNB6	21/66
Q9UI12	ATP6V1H	CGI-11	66/66
Q9UID3	C11orf2	ANG2	62/62
Q9UJ70	NAGK	GNK	47/64
Q9UJU6	DBNL	HIP-55	55/66
Q9UKK3	PARP4	ADPRTL1	56/65
Q9UL25	RAB21	KIAA0118	58/64
Q9UMR2	DDX19A	DDX19L	47/65
Q9UMS4	PRPF19	hPSO4	62/65

Q9UN37	VPS4A	FLJ22197	63/65
Q9UNM6	PSMD13	p40.5	46/63
Q9UNP9	PPIE	CyP-33	57/64
Q9UPU5	USP24	KIAA1057	50/66
Q9UQ80	PA2G4		66/66
Q9UQE7	SMC3		53/64
Q9Y224	C14orf166	CGI-99	63/63
Q9Y230	RUVBL2	ECP51	63/63
Q9Y263	PLAA	DOA1	48/66
Q9Y265	RUVBL1	ECP54	45/66
Q9Y281	CFL2		62/62
Q9Y285	FARSA	CML33	61/62
Q9Y2I8	WDR37	KIAA0982	64/64
Q9Y2L1	DIS3	dis3p	63/64
Q9Y2L5	TRAPPC8	GSG1	53/63
Q9Y2V7	COG6	COD2	60/64
Q9Y3F4	STRAP	MAWD	65/65
Q9Y3I0	C22orf28	HSPC117	64/65
Q9Y3Z3	SAMHD1	AGS5	28/65
Q9Y490	TLN1	ILWEQ	60/66
Q9Y4D7	PLXND1	KIAA0620	49/64
Q9Y5B9	SUPT16H	CDC68	50/64
Q9Y5P6	GMPPB	IAA1851	58/65
Q9Y5Y2	NUBP2	CFD1	51/62
Q9Y624	F11R	CD321	59/65
Q9Y678	COPG	COPG1	49/64
Q9Y6K5	OAS3		28/65
Q9Y6W5	WASF2	SCAR2	60/65
