# Supplementary material for: Characterization of Human Thymic Exosomes
Source: PLoS One. 2013 Jul 2;8(7):e67554. doi: 10.1371/journal.pone.0067554 (PMC3699640; doi:10.1371/journal.pone.0067554)
Supplement: Table S3 — Proteins found in one or the other of the two induvidual exosomal samples. (PDF) [file pone.0067554.s004.pdf]

Uniprot	Gene	Gene syn	Cell type expression in HPA
O00522	KRIT1	CAM	38/66
P12814	ACTN1		21/66
P62942	FKBP1A	FKBP-12	23/64
P09382	LGALS1	GBP	30/64
Q8TD55	PLEKHO2	DKFZp761K2312	25/63
Q9H299	SH3BGRL3		21/64
P40121	CAPG	AFCP	12/65
Q02388	COL7A1	EBD1	16/66
Q15052	ARHGEF6	alpha-PIX	24/63
P16150	SPN	CD43	21/63
P19878	NCT2	NOXA2	6/65
Q86VB7	CD163	M130	8/66
P11215	ITGAM	CD11B	15/65
Q92619	HMHA1	ARHGAP45	15/62
Q9H3G5	CPVL		21/63
P09972	ALDOC		8/66
P51784	USP11	UHX1	12/66
P98095	FBLN2		12/65
P20585	MSH3	DUP	5/66
P12883	MYH7	CMD1S	2/66
Q86U38	C14orf21		6/65
O95819	MAP4K4	FLH21957	18/65
P01024	C3	CPAMD1	16/66
O43149	ZZEF1	FLJ10821	15/66
O14638	ENPP3	B10	10/66
Q96Q05	TRAPPC9	IKBKBBP	16/64
P02749	APOH	B2G1	6/65
O60826	CCDC22	CXorf37	3/66
P00488	F13A1	F13A	1/66
Q9BXN1	ASPN	FLJ20129	1/66
Q13325	IFIT5	RI58	10/66
Q05315	CLC	LGALS10	2/65
Q13291	SLAMF1	CD150	5/64
Q5JV73	FRMPD3	KIAA1817	5/65
Q9NNX6	CD209	CDSIGN	4/65
Q9UDY8	MALT1	MLT	3/66
P62877	RBX1	BA554C12.1	14/62
O95757	HSPA4L	APG-1	7/64
Q99719		H5	13/65
P15924	DSP	DPI	16/65
Q9NZT1	CALML5	CLSP	8/63
P47929	LGALS7	GAL7	
Q9UBG3	CRNN	C1orf10	7/64
Q96KP1	EXOC2	FLJ11026	9/64
Q9H0W9	C11orf54	PTD012	10/66

P59768	GNG2		32/63
Q13642	FHL1	bA535K18.1	13/65
P45985	MAP2K4	JNKK1	32/66
Q53GL7	PARP10	FLJ14464	33/65
Q9H267	VPS33B	FLJ14848	19/64
P29350	PTPN6	HCP	13/66
P20963	CD247	CD3H	9/65
P30203	CD6	Tp120	9/65
P11836	MS4A1	B1	9/65
Q06187	BTK	AGMX1	10/66
Q86WV1	SKAP1	SCAP1	17/65
Q8IUI8	CRLF3	CREME9	35/62
P42574	CASP3	apopain	27/66
Q9NRZ9	HELLS	LSH	30/64
P48960	CD97	TM7LN1	41/66
P35236	PTPN7	HEPTP	24/66
Q92835	INPP5D	hp51CN	22/65
P09693	CD3G		27/64
Q96H79	ZC3HAV1L	C7orf39	34/66
O94768	STK17B	DRAK2	43/64
Q9BZL6	PRKD2	DKFZP586E0820	46/66
Q9BQ67	GRWD1	GRWD	33/66
Q9NR56	MBNL1	EXP	39/66
O15160	POLR1C	RPA39	22/64
P04040	CAT		29/66
P49419	ALDH7A1	ATQ1	40/65
Q7Z3U7	MON2	KIAA1040	22/65
Q02952	AKAP12	AKAP250	28/64
P04080	CSTB	CST6	9/66
Q9UH65	SWAP70	KIAA0640	35/63
O75116	ROCK2		49/65
Q96EE3	SEH1L	SEC13L	27/63
P13489	RNH1	RAI	44/65
P04049	RAF1	c-Raf	66/66
P34947	GRK5	GPRK5	50/65
P51610	HCFC1	CFF	35/65
Q9NR48	ASH1L	ASH1	29/63
Q00005	AC011357.1		34/64
P55769	NHP2L1	15.5K	36/65
Q9BQ39	DDX50	GU2	27/64
Q9NPF5	DMAP1	DNMAP1	36/66
O14672	ADAM10	CD156c	29/64
P23229	ITGA6	CD49f	35/65
P61421	ATP6V0D1	ATP6D	37/65
Q8WWH5	TRUB1	PUS4	35/65
P35611	ADD1		41/66

P34913	EPHX2		41/66
Q12792	TWF1	A6	41/65
O94874	KIAA0776	NLBP	43/66
Q9H3N1	TMX1	PDIA11	41/65
P54105	CLNS1A	CLCI	38/63
Q9Y262	EIF3L	EIF3EIP	40/65
P57772	EEFSEC	EFSEC	31/66
Q9NP79	VTA1	C6orf55	38/66
Q15833	STXBP2	Hunc18b	30/65
Q96PX9	PLEKHG4B	KIAA1909	27/66
Q15785	TOMM34	HTOM34P	37/66
P14324	FDPS		45/65
Q9NYT0	PLEK2		40/64
P08962	CD63	ME491	35/66
Q86W50	METTL16	METT10D	41/65
Q8WUH2	TGFBRAP1	TRAP-1	32/62
Q9BTT0	ANP32E	LANP-L	42/65
O43314	PPIP5K2	HISPPD1	36/63
Q13107	USP4	UNP	39/66
Q9H944	MED20	DKFZp586D2223	31/64
O00255	MEN1		36/65
Q7Z2T5	TRMT1L	c1orf25	39/66
Q14914	PTGR1	LTB4DH	37/65
Q96QU8	XPO6	FLJ22519	37/63
Q6PJG6	BRAT1	BAAT1	45/65
Q15813	TBCE	HRD	47/64
Q9BUI4	POLR3C	RPC3	53/66
P16422	EPCAM	17-1A	22/66
P25445	FAS	APO-1	28/64
Q9Y2J2	EPB41L3	4.1B	21/65
Q00013	MPP1	DXS552E	25/65
Q8IWA5	SLC44A2	CTL2	15/64
Q06203	PPAT	GPAT	35/65
Q8NHH9	ATL2	ARL6IP2	27/64
Q96MM6	HSPA12B	C20orf60	12/65
P55212	CASP6	MCH2	31/66
Q86YJ6	THNSL2	FLJ10916	26/64
Q7Z6J4	FGD2	ZFYVE4	15/64
Q7Z3J2	C16orf62	MGC16824	19/64
Q9NQW7	XPNPEP1	XPNPEP	25/66
P20702	ITGAX	CD11c	29/65
Q92598	HSPH1	HSP105A	37/64
Q53GS9	USP39	CGI-21	38/65
Q9H0C2	SLC25A31	ANT4	27/66
Q9Y530	C6orf130	dJ34B21.3	32/66
O60341	KDM1A	AOF2	56/63

O75569	PRKRA	DYT16	54/63
O00139	KIF2A	HK2	40/64
P16949	STMN1	C1orf215	50/65
P18074	ERCC2	EM9	51/64
O43665	RGS10		54/65
P26232	CTNNA2	CAP-R	61/66
Q99986	VRK1		42/66
P08473	MME	CALLA	25/64
O60749	SNX2		41/64
Q9BSJ8	ESYT1	FAM62A	42/62
Q9NRW3	APOBEC3C	APOBEC1L	44/66
P06756	ITGAV	CD51	45/66
Q5TEJ8	C1orf38	ICB-1	45/65
Q8WXH0	SYNE2	DKFZP434H2235	42/64
O94822	LTN1	C21orf10	48/64
P43378	PTPN9	MEG2	54/65
Q9UM54	MYO6	DFNA22	43/64
P12429	ANXA3	ANX3	55/66
Q9BV40	VAMP8	EDB	43/65
Q13740	ALCAM	CD166	35/65
O00750	PIK3C2B	C2-PI3K	27/65
Q6IA17	SIGIRR	TIR8	39/64
Q9H9T3	ELP3	FLJ10422	40/62
P00533	EGFR	ERBB	32/65
Q9Y316	MEMO1	C2orf4	42/64
O43813	LANCL1	GPR69A	49/64
P02452	COL1A1	OI4	53/64
P35250	RFC2	A1	51/66
P49916	LIG3	LIG2	58/65
Q8WVM8	SCFD1	C14orf163	60/63
O00425	IGF2BP3	CT98	55/66
P19525	EIF2AK2	EIF2AK1	52/64
Q08379	GOLGA2	GM130	53/66
Q9NZJ9	NUDT4	DIPP2	53/64
P34932	HSPA4	HS24/P52	53/66
Q4G0J3	LARP7	DKFZP564K112	52/65
Q8N1G2	FTSJD2	KIAA0082	55/66
Q8IYI6	EXOC8	EXO84	52/66
P62888	RPL30	L30	48/65
P63220	RPS21	S21	54/63
Q99653	AC012652.1		57/65
Q9Y2A7	NCKAP1	HEM2	45/65
O43264	ZW10	KNTC1AP	54/66
Q53HC9	TSSC1		46/65
P35080	PFN2		37/64
Q9BQA1	WDR77	MEP50	42/66

O15511	ARPC5	ARC16	47/65
Q9BPX5	ARPC5L	ARC16-2	47/65
Q86X76	NIT1		40/63
Q10471	GALNT2	GalNAc-T2	50/65
Q9Y3B3	TMED7	CGI-109	61/66
Q04941	PLP2	A4	43/64
P16278	GLB1	EBP	53/62
Q9UI30	TRMT112	HSPC152	45/65
P30086	PEBP1	HCNP	43/65
Q5T6J7	C9orf103	bA522I20.2	43/65
Q9BYB4	GNB1L	GY2	50/66
O43795	MYO1B	myr1	45/64
O60234	GMFG		48/64
P08621	SNRNP70	RNPU1Z	58/62
P10155	TROVE2	SSA2	42/65
P49137	MAPKAPK2		52/64
P25325	MPST	MST	47/62
Q9NP97	DYNLRB1	DNCL2A	59/66
P41217	CD200	MOX1	59/66
Q99747	NAPG		55/65
Q9BZG1	RAB34	RAB39	54/65
P17987	TCP1	CCT1	64/66
P32019	INPP5B		58/65
P42858	HTT	HD	57/66
O75150	RNF40	BRE1B	56/65
Q6PJ69	TRIM65		61/64
Q5JSH3	WDR44	DKFZp686L20145	57/65
O14733	MAP2K7	Jnkk2	58/66
P19474	TRIM21	RNF81	54/65
P48147	PREP		61/66
Q99615	DNAJC7	TPR2	56/64
Q9Y2Z0	SUGT1	SGT1	52/64
Q14289	PTK2B	CADTK	45/64
Q9BZ23	PANK2	C20orf48	55/63
P57764	GSDMD	DF5L	47/66
Q9Y4E8	USP15	KIAA0529	46/64
P06703	S100A6	2A9	61/66
P18206	VCL		54/66
Q9Y6K9	IKBKG	FIP-3	57/66
P30154	PPP2R1B	PP2A-Abeta	60/66
Q04323	UBXN1	2B28	51/64
Q15185	PTGES3	cPGES	54/63
P00390	GSR		53/66
Q9UJX2	CDC23	ANAPC8	57/65
Q07866	KLC1	hKLC1B	52/63
Q8N3P4	VPS8	FLJ32099	40/65

Q9Y5Z4	HEBP2	C6orf34	52/62
P41214	EIF2D	LGTN	42/65
P52209	PGD		48/66
Q96AX1	VPS33A		46/64
Q8N0W3	FUK	FLJ39408	46/64
Q9NSK0	KLC4	bA387M24.3	49/66
P30519	HMOX2	HO-2	66/66
Q99808	SLC29A1	ENT1	56/65
Q86U86	PBRM1	BAF180	52/62
O60333	KIF1B	CMT2	49/66
P48507	GCLM	GLCLR	58/65
Q9Y223	GNE	IBM2	53/65
Q14232	EIF2B1	EIF-2B	53/65
Q5EBM0	CMPK2	TYKi	48/66
Q9UGJ1	TUBGCP4	76P	52/65
P06746	POLB		56/65
Q8N122	RPTOR	KIAA1303	59/65
Q9NZD8	SPG21	ACP33	53/66
Q5BKZ1	ZNF326	FLJ20403	56/65
Q9UKF6	CPSF3	CPSF-73	54/64
P54710	FXYD2	ATP1G1	46/65
Q15018	FAM175B	ABRO1	49/63
Q9H8Y8	GORASP2	GOLPH6	46/66
Q9H4A6	GOLPH3	GOPP1	45/66
Q9ULP9	TBC1D24	KIAA1171	46/66
Q8IYJ3	SYTL1	exophilin-7	50/64
Q9P2P6	STARD9	KIAA1300	52/65
Q9UI10	EIF2B4	DKFZP586J0119	48/64
Q01469	FABP5	E-FABP	27/64
O14929	HAT1	KAT1	43/65
Q13144	EIF2B5	EIF-2B	46/66
P49247	RPIA		51/66
Q96CD0	FBXL8	Fbl8	50/65
Q9NR30	DDX21	GURDB	46/65
P10599	TXN	TRX	43/61
Q7Z4L5	TTC21B	FLJ11457	30/63
Q9HCY8	S100A14	BCMP84	34/66
Q96TA1	FAM129B	bA356B19.6	40/66
Q9HAS0	C17orf75	NJMU-R1	39/61
P42330	AKR1C3	DDX	39/66
Q9NQR4	NIT2		52/65
P15531	NME1	NM23	56/62
O95361	TRIM16	EBBP	66/66
P30566	ADSL		66/66
Q9ULR0	ISY1	fSAP33	65/65
Q969E2	SCAMP4	FLJ33847	54/64

Q99623	PHB2	Bap37	59/61
P51608	MECP2	MRX16	64/64
P06748	NPM1	B23	64/64
P35659	DEK	D6S231E	62/62
P50750	CDK9	C-2k	65/65
P09651	HNRNPA1	hnRNP-A1	64/64
Q12824	SMARCB1	BAF47	66/66
P49959	MRE11A	ATLD	66/66
P61289	PSME3	Ki	64/64
Q49A26	GLYR1	BM045	64/64
Q92905	COPS5	CSN5	64/64
P07384	CAPN1	CANP	64/64
Q9UHQ9	CYB5R1	humb5R2	65/65
P21127	CDK11A	CDC2L2	65/65
Q6P3X3	TTC27	FLJ20272	63/65
Q9Y5X3	SNX5		66/66
Q99798	ACO2	ACONM	61/61
O60506	SYNCRIP	dJ3J17.2	63/64
P54577	YARS	tyrRS	66/66
Q96T60	PNKP	PNK	61/63
Q01968	OCRL	OCRL1	65/66
P35270	SPR	SDR38C1	64/64
P46060	RANGAP1	Fug1	62/62
P63218	GNG5		65/65
Q14149	MORC3	KIAA0136	65/65
Q92542	NCSTN	APH2	63/63
O14972	DSCR3	DCRA	64/64
P62851	RPS25	S25	65/65
O43747	AP1G1	ADTG	65/65
Q96S55	WRNIP1	bA420G6.2	63/65
O95671	ASMTL		64/65
Q9BZK7	TBL1XR1	C21	64/66
Q86WJ1	CHD1L	ALC1	66/66
P31939	ATIC	AICARFT	66/66
Q5VTR2	RNF20	BRE1	65/66
Q12996	CSTF3	CstF-77	64/64
Q9NT62	ATG3	APG3L	64/65
Q9NXR7	BRE	BRCC4	63/65
P49840	GSK3A		66/66
P51809	VAMP7	SYBL1	63/64
P61221	ABCE1	OABP	65/66
P30419	NMT1	NMT	66/66
Q8IVG5	SAMD9L	C7orf6	66/66
P19174	PLCG1	NCKAP3	66/66
Q99459	CDC5L	CDC5	66/66
Q8NHV4	NEDD1	GCP-WD	64/64

Q9UHD9	UBQLN2	Chap1	66/66
Q96GM5	SMARCD1	BAF60A	62/63
Q9UP83	COG5	GOLTC1	63/65
Q6P6C2	ALKBH5	FLJ20308	58/58
Q9H0C8	ILKAP	DKFZP434J2031	62/64
O75787	ATP6AP2	APT6M8-9	59/62
O75915	ARL6IP5	DERP11	66/66
P42566	EPS15	AF-1P	66/66
P14678	SNRPB	COD	65/65
Q16543	CDC37	P50CDC37	64/66
Q70CQ1	USP49	MGC20741	61/66
P17612	PRKACA	PKACa	62/65
Q9Y6G9	DYNC1LI1	DNCLI1	58/66
P00441	SOD1	ALS	66/66
P61962	DCAF7	HAN11	60/62
P50502	ST13	FAM10A1	59/63
O00429	DNM1L	DRP1	61/64
Q9NRW7	VPS45	h-vps45	56/66
Q9ULC3	RAB23		63/65
P35754	GLRX	GRX	64/64
P49770	EIF2B2	EIF-2Bbeta	58/64
P62318	SNRPD3	Sm-D3	60/63
Q0VGL1	C7orf59		53/65
P24534	EEF1B2		57/63
Q8N201	INTS1	DKFZp586J0619	59/65
Q8NBF2	NHLRC2	DKFZp779F115	58/65
Q9H3P7	ACBD3	GCP60	58/63
Q9NS87	KIF15	HKLP2	57/65
Q9Y3B4	AC008073.5		60/63
P25685	DNAJB1	Hsp40	58/66
P29401	TKT		64/65
Q96KP4	CNDP2	CN2	56/66
Q96LJ7	DHRS1	FLJ25430	60/62
O75436	VPS26A	Hbeta58	57/66
Q53EL6	PDCD4	H731	58/65
Q8N9N7	LRRC57	FLJ36812	52/64
Q9HBL8	NMRAL1	FLJ25918	57/64
O14727	APAF1	APAF-1	58/66
P43007	SLC1A4	ASCT1	63/63
Q99497	PARK7	DJ-1	64/64
Q9UBI1	COMMD3	BUP	59/63
Q9NUQ8	ABCF3	EST201864	60/64
Q9NRR5	UBQLN4	A1U	57/66
Q9UJX3	ANAPC7	APC7	57/63
Q13618	CUL3		59/63
O75348	ATP6V1G1	ATP6G	55/64

P26639	TARS		61/64
O60763	USO1	p115	52/65
Q9Y2J8	PADI2	KIAA0994	63/66
P04844	RPN2	RIBIIR	66/66
Q8NB66	UNC13C	DKFZp547H074	62/63
Q5VVH5	IRAK1BP1	AIP70	58/64
P50851	LRBA	BGL	66/66
Q9UK41	VPS28		66/66
Q6IA86	ELP2	FLJ10879	61/66
O00178	GTPBP1	GP-1	57/64
P18433	PTPRA	HLPR	58/66
Q9BRZ2	TRIM56	RNF109	52/65
Q14746	COG2	LDLC	53/65
Q9H7D7	WDR26	FLJ21016	56/66
Q6WKZ4	RAB11FIP1	FLJ22524	57/64
Q9H2K8	TAOK3	DPK	60/65
Q9NV70	EXOC1	BM-102	57/64
Q16537	PPP2R5E		58/64
Q8NEB9	PIK3C3	Vps34	56/65
P40692	MLH1	COCA2	52/59
O00635	TRIM38	RNF15	53/65
P39748	FEN1	FEN-1	59/66
P34897	SHMT2	SHMT	58/66
Q13459	MYO9B	CELIAC4	62/66
Q32MZ4	LRRFIP1	FLAP-1	54/63
O95487	SEC24B		58/64
Q8NE71	ABCF1	ABC50	60/65
Q93084	ATP2A3	SERCA3	63/66
Q9H9H4	VPS37B	FLJ12750	58/63
Q9UNA1	ARHGAP26	GRAF	56/64
O60684	KPNA6	FLJ11249	64/66
Q9NR50	EIF2B3	EIF-2B	64/66
P42285	SKIV2L2	Dob1	64/64
Q53QZ3	ARHGAP15	BM046	63/64
Q9NW08	POLR3B	FLJ10388	64/65
O60911	CTSL2	CTSU	63/65
O00422	SAP18	2HOR0202	63/64
Q9UPT5	EXOC7	EXO70	64/64
P61803	DAD1	OST2	63/65
Q9P0L0	VAPA	hVAP-33	64/64
Q5TA45	CPSF3L	CPSF73L	64/64
Q86XI2	NCAPG2	CAP-G2	64/65
Q8WTW3	COG1	KIAA1381	64/66
Q8TAG9	EXOC6	DKFZp761I2124	58/65
Q6L8Q7	PDE12	2'-PDE	58/65
Q96DH6	MSI2		62/64

Q96IU4	ABHD14B	CIB	64/64
Q9Y2L8	ZKSCAN5	ZFP95	62/64
Q9Y4A5	TRRAP	PAF400	63/64
P07332	FES	FPS	59/64
Q12959	DLG1	dJ1061C18.1.1	59/66
Q99570	PIK3R4	p150	58/65
Q9Y5K5	UCHL5	CGI-70	62/65
Q8IZP0	ABI1	ABI-1	57/63
Q14254	FLOT2	ECS-1	64/66
Q13492	PICALM	CALM	62/66
P49754	VPS41	HVSP41	66/66
Q9UMZ2	SYNRG	AP1GBP1	66/66
Q9ULT0	TTC7A	KIAA1140	65/65
