# Supplementary material for: Characterization of Human Thymic Exosomes
Source: PLoS One. 2013 Jul 2;8(7):e67554. doi: 10.1371/journal.pone.0067554 (PMC3699640; doi:10.1371/journal.pone.0067554)
Supplement: Table S4 — miRNAs in human thymic exosomes. (PDF) [file pone.0067554.s005.pdf]

systematic_name
SCorner3
dmr_285
dmr_3
dmr_31a
dmr_6
ebv-miR-BART12
ebv-miR-BART13
ebv-miR-BART16
hcmv-miR-US33-5p
hsa-let-7b
hsa-miR-1182
hsa-miR-1183
hsa-miR-1202
hsa-miR-1207-5p
hsa-miR-1224-5p
hsa-miR-1225-3p
hsa-miR-1225-5p
hsa-miR-1226*
hsa-miR-1228
hsa-miR-1234
hsa-miR-1237
hsa-miR-1238
hsa-miR-1246
hsa-miR-1249
hsa-miR-125a-3p
hsa-miR-125b-2*
hsa-miR-1268
hsa-miR-1274b
hsa-miR-1275
hsa-miR-1280
hsa-miR-1281
hsa-miR-1290
hsa-miR-1299
hsa-miR-1308
hsa-miR-134
hsa-miR-1471
hsa-miR-149*
hsa-miR-150*
hsa-miR-181b
hsa-miR-1825
hsa-miR-188-5p
hsa-miR-191*
hsa-miR-1915
hsa-miR-198
hsa-miR-23a*

hsa-miR-30b*
hsa-miR-30c-1*
hsa-miR-32*
hsa-miR-320b
hsa-miR-320c
hsa-miR-33b*
hsa-miR-371-5p
hsa-miR-422a
hsa-miR-423-5p
hsa-miR-425*
hsa-miR-483-5p
hsa-miR-557
hsa-miR-572
hsa-miR-574-5p
hsa-miR-575
hsa-miR-583
hsa-miR-595
hsa-miR-601
hsa-miR-610
hsa-miR-622
hsa-miR-623
hsa-miR-630
hsa-miR-638
hsa-miR-720
hsa-miR-760
hsa-miR-765
hsa-miR-766
hsa-miR-877*
hsa-miR-923_v12.0
hsa-miR-933
hsa-miR-939
hsa-miR-940
hsv1-miR-H1_v14.0
hur_1
hur_2
hur_4
kshv-miR-K12-3
miRNABrightCorner30
